# Neutrophil Transcriptional Deregulation by the Periodontal Pathogen Fusobacterium nucleatum in Gastric Cancer: A Bioinformatic Study

**DOI:** 10.1155/2022/9584507

**Published:** 2022-08-18

**Authors:** Ting Zhou, Xianhong Meng, Daxiu Wang, Weiran Fu, Xinrui Li

**Affiliations:** The Ward No. 2, Department of Gastroenterology, The Fourth Affiliated Hospital of Harbin Medical University, No. 37 Yiyuan Street, Nangang District, Harbin, 150001 Heilongjiang, China

## Abstract

**Background:**

Infection with the periodontal pathogen *Fusobacterium nucleatum* (*F. nucleatum*) has been associated with gastric cancer. The present study is aimed at uncovering the putative biological mechanisms underlying effects of *F. nucleatum*–mediated neutrophil transcriptional deregulation in gastric cancer.

**Materials and Methods:**

A gene expression dataset pertaining to *F. nucleatum*-infected human neutrophils was utilized to identify differentially expressed genes (DEGs) using the GEO2R tool. Candidate genes associated with gastric cancer were sourced from the “Candidate Cancer Gene Database” (CCGD). Overlapping genes among these were identified as link genes. Functional profiling of the link genes was performed using “g:Profiler” tool to identify enriched Gene Ontology (GO) terms, pathways, miRNAs, transcription factors, and human phenotype ontology terms. Protein-protein interaction (PPI) network was constructed for the link genes using the “STRING” tool, hub nodes were identified as key candidate genes, and functionally enriched terms were determined.

**Results:**

The gene expression dataset GEO20151 was downloaded, and 589 DEGs were identified through differential analysis. 886 candidate gastric cancer genes were identified in the CGGD database. Among these, 36 overlapping genes were identified as the link genes. Enriched GO terms included molecular function “enzyme building,” biological process “protein folding,'” cellular components related to membrane-bound organelles, transcription factors ER71 and Sp1, miRNAs miR580 and miR155, and several human phenotype ontology terms including squamous epithelium of esophagus. The PPI network contained 36 nodes and 53 edges, where the top nodes included PH4 and CANX, and functional terms related to intracellular membrane trafficking were enriched.

**Conclusion:**

*F nucleatum*-induced neutrophil transcriptional activation may be implicated in gastric cancer via several candidate genes including DNAJB1, EHD1, IER2, CANX, and PH4B. Functional analysis revealed membrane-bound organelle dysfunction, intracellular trafficking, transcription factors ER71 and Sp1, and miRNAs miR580 and miR155 as other candidate mechanisms, which should be investigated in experimental studies.

## 1. Introduction

Gastric cancer is considered the sixth most common cancer globally [1]. A majority of gastric cancer cases occur in developing nations, and it is one of the chief causes of cancer-related morbidity and mortality [2]. Microbial factors are understood to play a central role in gastric cancer pathogenesis, and the best established among these is *Helicobacter pylori* (*H. pylori*) infection [3, 4]. An increasing number of studies have shown an association of several specific microbial species and the gastric microbial community or microbiome's composition with gastric cancer [5–8].

Recently, a meta-analysis of gastric mucosa and associated microbiota demonstrated the periodontal pathogens *Fusobacterium nucleatum* (*F. nucleatum*), *Parvimonas micra*, and *Peptostreptococcus stomatis* as interacting and hub nodes associated with other gastric cancer-associated species and tumor status [9]. The periodontal pathogen *F. nucleatum* has been most strongly implicated in colorectal cancer (CRC) and is known to induce inflammation and suppress anticancer immune responses in CRC. *F. nucleatum* infection of neutrophils is known to induce NETosis [10]. In CRC, the circulatory transmission of *F. nucleatum* is the dominant mechanism [11], which suggests that systemic *F. nucleatum* and its immune signatures may be similarly relevant in other associated cancers. In particular, some *F. nucleatum* strains are shown to impede neutrophil-mediated oxidative killing [12], which could be implicated in its role in gastric cancer pathogenesis. In case of *H. pylori*, also a gram-negative pathogen, infection is also shown to promote N1 neutrophil subtype marked by nuclear hypersegmentation [13] but such mechanisms in case of *F. nucleatum* stimulated neutrophils are not yet investigated. As neutrophils play a central role in the tumor microenvironment [14], the role of *F. nucleatum*-induced neutrophil deregulation in gastric cancer merits further investigation. Tumor-activated neutrophils infiltrate the lesion and play a key role in the progression of gastric cancer via STAT3-related mechanisms [15], and the interaction of gastric cancer cells with tumor neutrophils promotes their migration, epithelial-mesenchymal transformation (EMT), and invasion [16]. Considering the paucity of research in this domain, bioinformatic approaches may reveal neutrophil transcriptional mechanisms relevant to gastric cancer. Therefore, the present study focused on uncovering neutrophil-related genes and molecular factors, which could be considered candidate mechanisms in gastric cancer via bioinformatic investigation.

## 2. Methods

### 2.1. Data Procurement and Link Gene Identification

Gene expression data for *F. nucleatum*-mediated regulation of neutrophil genes was sourced; the gene expression dataset GEO20151 [17] describing *F. nucleatum*-mediated regulation of neutrophil genes was downloaded from the Gene expression omnibus (GEO). Differential gene expression (DEG) analysis was performed using the GEO2R tool. Data were log transformed and normalized, and limma precision weights were applied. A significance level cut-off of *p* = 0.05 with Benjamini and Hochberg (false discovery rate) correction was used to screen DEGs. Candidate human genes associated with gastric cancer from all available studies in the database were downloaded from the “Candidate Cancer Gene Database (CCGD)” [18]. The DEGs and candidate gastric cancer genes identified in the earlier step were overlapped using a Venn diagram, and shared genes were identified as “link” genes between *F. nucleatum*-mediated neutrophil transcriptome alteration and gastric cancer.

### 2.2. Functional Profiling of Link Genes

The link genes list was subjected to functional profiling analysis using the web-based tool “Gprofiler” [19]. Here, the organism of interest was selected as “Human,” only annotated genes were used as input, and the customized algorithm g:SCS significance threshold set at 0.05 was used for identification of enriched terms that was used.

### 2.3. Protein-Protein Interaction (PPI) Network and Functional Enrichment Analysis

PPI network construction with the link gene list as input was done using the STRING webtool [20]. A full STRING network with interaction sources including text mining, experiments, databases, coexpression, neighborhood, gene fusion, and co-occurrence was constructed. A minimum required interaction score was set as 0.15, and network edges represented the confidence measure. Network characteristics, “hub” genes, and functionally enriched terms in the network were determined.

## 3. Results

### 3.1. Link Gene Identification

The analysis of the gene expression dataset GEO20151 identified 589 annotated DEGs (Table S1).  Table 1 displays the top 20 DEGs ranked by the adjusted *p* value.

Using the CCGD database, 886 annotated candidate gastric cancer human genes were identified (Table S2).  Table 2 shows the top 20 candidate gastric cancer genes ranked by the number of supporting studies.

**Figure 1:**
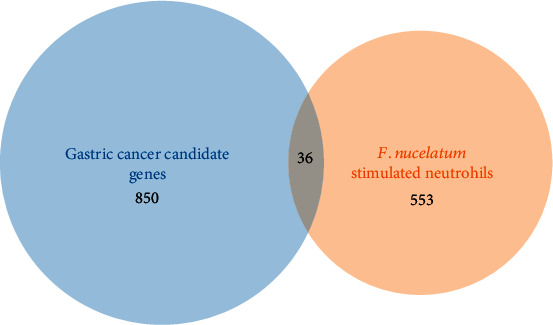
A Venn diagram depicting 589 annotated *F. nucleatum*-stimulated netrophil DEGs, 886 annotated candidate gastric cancer genes, and 36 common “link” genes.

A Venn diagram was constructed, and the overlapping genes were identified, which showed 36 link genes (Figure 1). The 36 link genes are listed in Table 3.

### 3.2. Functional Profiling of the Link Genes

**Figure 2:**
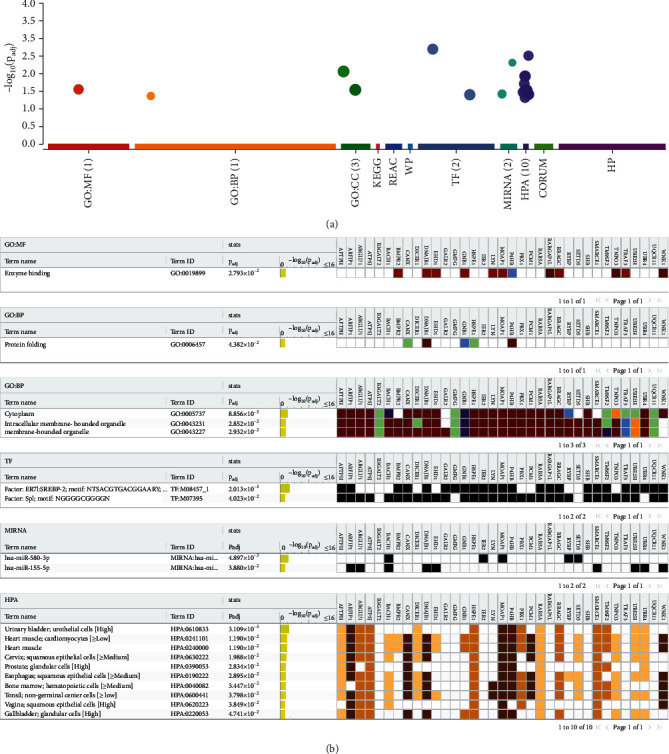
Functional enrichment analysis of the link genes. (a) Bubble plot depicting -log 10 *p* adjusted values of enriched terms. (b) Detailed results depicting 19 enriched terms.

The functional enrichment analysis results from “G:profiler” are depicted in Figure 2. These included 1 GO molecular function term (enzyme binding), 1 GO biological process term (protein folding), 3 GO cellular component terms (cytoplasm, intracellular membrane-bounded organelle, membrane-bounded organelle), 2 transcription factors (ER71 and Sp1), 2 miRNAs (miR 580, miR 155), and 10 human phenotype ontology terms (Table S3).

### 3.3. PPI Network and Functional Enrichment Analysis

**Figure 3:**
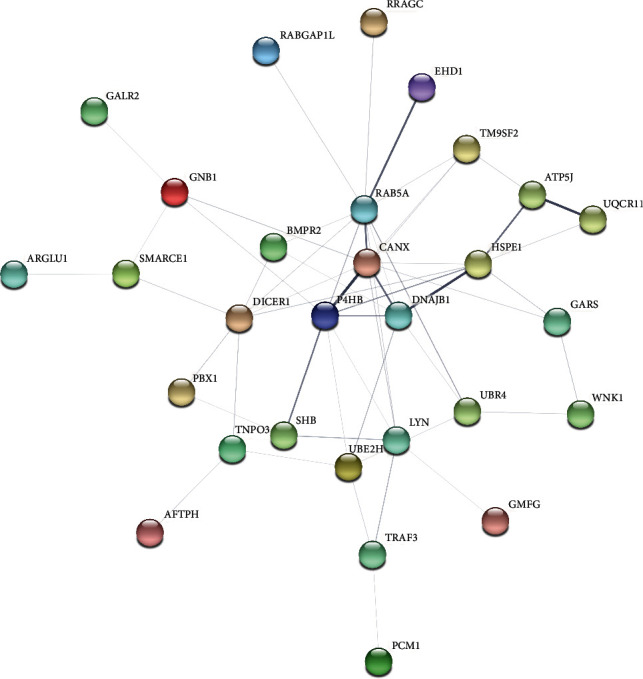
PPI network analysis of the 36 link genes. The top 5 enriched nodes included CANX, PH4B, ATP5J, DNAJB1, and EHD1.

The PPI network had 36 nodes and 54 edges with an average node degree of 3 and an average local clustering coefficient of 0.386 (Figure 3). The top 5 enriched nodes included CANX, PH4B, ATP5J, DNAJB1, and EHD1. 32 enriched functional terms in 3 categories were identified (Table 4).

Functional enrichment analysis depicted multiple terms related to Extracellular exosomes, extracellular organelle, extracellular vesicle and membrane protein complex and tissues including blood cells and digestive glands (Table 4).

## 4. Discussion

The present identified key molecular mechanisms, which may link *F. nucleatum*-stimulated neutrophil transcriptomic alterations with the development of gastric cancer. Among the DEGs in *F. nucleatum*-stimulated neutrophils, 36 genes were documented as gastric cancer candidate genes. The most significant genes among these included DNAJB1, EHD1, and IER2. DnaJ/Hsp40 (heat shock protein 40) proteins are key proteins for protein biology via stimulation of ATPase and are shown to play a role in p53 ubinquination to promote cancer cells in vitro [21]. EHD1 (Eps15 homology (EH) domain-containing protein 1) plays an important role in receptor-mediated endocytic recycyling [22], shows to promote tumor growth, and is implicated in resistance to cisplatin in case of non-small-cell lung cancer [23]. Human immediate early response 2 (IER2) is a nuclear protein that is implicated in cancer via transcriptional regulation of endothelial motility and adhesion via a FAK-dependent mechanism [24], thereby regulating tumor angiogenesis. Apart from DNAJB1 and EHD1, the PPI network analysis showed CANX and PH4B as the top hub genes. Calnexin or CANX is an ER stress chaperone transmembrane protein involved in glycoprotein folding, is considered a prognostic indicator and therapeutic target in CRC [25], and is found to restrict antitumor CD4+ and CD8+ T cells [26] in oral cancer. The protein disulfide-isomerase P4HB also acts as a chaperone protein involved in protein folding and the ER stress response and is shown to be a prognostic marker of glioma [27]. In gastric cancer, HIF-1 is found to suppress P4HB and promote cancer cell proliferation [28]. PH4B is also linked to chemoresistance [29–31]. Emerging evidence indicates that neutrophil NETosis is a central contributor to cancer proliferation and chemoresistance [32]. Overall, the identified candidate genes may serve as molecular mechanisms underlying *F. nucleatum* neutrophil-stimulated NETosis with gastric cancer. Of note, NETosis has been documented in relation to *Helicobacter pylori* via NADPH oxidase activation through several kinases [33], which is well established in its association with gastric cancer [34]. Inflammatory mechanisms leading to NETosis activation via *F. nucleatum* in gastric cancer should be investigated. In addition, emerging evidence shows *F. nucleatum* as a factor increasing the chemoresistance in CRC by modulating the tumor microenvironment and autophagy [35, 36]. The plausible role of *F. nucleatum* infection in the chemoresistance of gastric cancer remains to be investigated.

Functional enrichment analysis of the link genes and PPI network was conducted, and consistency in the findings was evident. Several extracellular processes including exosome, membrane protein complex, vesicles, and intracellular membrane-bound organelle were seen as enriched components in the PPI network. Protein folding and associated cellular components were evident as enriched, underscoring the potential relevance of the ER stress response as a linkage mechanism [37]. The 2 enriched transcription factors included ER71 and Sp1. The Ets transcription factor Er71 is a key regulator in endothelial and hematopoietic stem cell development [38] and recently has been reported as a valuable target to block tumor angiogenesis [39]. SP1 is shown to transcriptionally regulate oncostatin M receptor in gastric cancer and thereby contribute to cancer progression [40]. SP1 is also implicated in neutrophil elastase-mediated increase in mucin gene receptors [41] and thus may play a role in stimulated neutrophil-mediated deregulation of the mucous barrier [42].

The role of *F. nucleatum* in CRC is well studied. It has multiple adhesins, and Fap2-mediated adhesion of *F. nucleatum* to epithelial cells is shown to induce a proinflammatory cascade, whereas Fap2-independent mechanisms are demonstrated in CRC neutrophils and macrophages, which together increase proinflammatory signaling to increase tumor invasion, seeding, and metastatsis [43]. In the colon, *F. nucleatum* is shown to disrupt epithelial barrier integrity by damage to tight junctions and induction of cytokines of helper T cells [44]. Pathogenic strains of *F. nucleatum* are shown to induce MUC2 and TNF secretion from colonic cells [45]. The interaction of *F. nucleatum* with mucins warrants further investigation in the context of gastric cancer. The 2 enriched miRNAs included miR 580 and miR 155. miR 580 has been shown to inhibit chemokine ligand 2 (CCL2) production in the hepatocellular carcinoma tumor microenvironment [46]. miR-155 is involved in neutrophil NETosis [47] and is considered a key factor interlinking inflammation with cancer [48]. miR-155 was found to play a tumor suppressor role in gastric cancer [49]. The enriched GO terms and compartments in the PPI network supported the role of intracellular membrane trafficking as a key cancer mechanism harnessed by *F. nucleatum* stimulation of neutrophils [50].

Taken together, the findings of this bioinformatic analysis revealed several possible molecular mechanisms by *F. nucleatum-*induced neutrophil gene deregulation that may promote gastric carcinogenesis. At the same time, these findings are limited by the small sample number in the analyzed gene expression dataset and the lack of validation experiments to support the relevance of the highlighted candidate genes, transcription factors, cellular processes, and miRNAs. Furthermore, the effects of *F. nucleatum* are likely to be subspecies or strain-specific and should be investigated in future research. *F. nucleatum* strains with higher invasive capacity have been identified in inflamed colonic tissues as compared to those from healthy tissues [51], which raises the need for phylotype and functional characterization in context of its role gastric cancer. The present findings should be verified in experimental research models that investigate the candidate link genes and functional mechanisms involved in *F. nucleatum*-mediated neutrophil plasticity relevant to gastric cancer pathogenesis. Cell model experiments, animal experiments, and clinical examination of the theoretical premises established in this study are warranted. The present investigation focused on the role of *F. nucleatum*-stimulated neutrophils alone in gastric cancer but the tumor microenvironment constitutes of varied immune cell populations that may be deregulated by *F. nucleatum* and also warrant deeper investigation.

## 5. Conclusion

*F nucleatum*-induced neutrophil transcriptional activation may be implicated in gastric cancer via several candidate genes including DNAJB1, EHD1, IER2, CANX, and PH4B among the top genes of interest. Putative key functional mechanisms included membrane-bound organelle dysfunction and intracellular trafficking along with the modulation of transcription factors ER71 and Sp1 and miRNAs miR580 and miR155.

## Figures and Tables

**Table 1:** Top 20 DEGs ranked by the adjusted *p* value.

Gene ID	Gene name	Adjusted *p* value	Log fold change
DNAJB1	DnaJ heat shock protein family (Hsp40) member B1	0.003	-2.13
CXCL3	C-X-C motif chemokine ligand 3	0.003	-4.13
FOS	Fos protooncogene, AP-1 transcription factor subunit	0.003	-2.44
HMOX1	Heme oxygenase 1	0.003	-2.29
HSPA1B///HSPA1A	Heat shock protein family A (Hsp70) member 1B///heat shock protein family A (Hsp70) member 1A	0.003	-2.28
HSPA1L///HSPA1B///HSPA1A	Heat shock protein family A (Hsp70) member 1-like///heat shock protein family A (Hsp70) member 1B///heat shock protein family A (Hsp70) member 1A	0.004	-2.04
OSM	Oncostatin M	0.004	-2.34
VEGFA	Vascular endothelial growth factor A	0.004	-1.55
MIR612///NEAT1	MicroRNA 612///nuclear paraspeckle assembly transcript 1 (nonprotein coding)	0.005	-1.51
HSP90AA1	Heat shock protein 90 alpha family class A member 1	0.006	-1.2
CKS2	CDC28 protein kinase regulatory subunit 2	0.006	-2.33
CXCL2	C-X-C motif chemokine ligand 2	0.008	-1.1
BTG2	BTG antiproliferation factor 2	0.008	-1.4
CSF1	Colony-stimulating factor 1	0.008	-2.12
FFAR2	Free fatty acid receptor 2	0.008	1.48
HILPDA	Hypoxia inducible lipid droplet associated	0.008	1.95
IL1RN	Interleukin 1 receptor antagonist	0.008	1.03
LOC100129518///SOD2	Uncharacterized LOC100129518///superoxide dismutase 2, mitochondrial	0.008	-1.09
MARCKS	Myristoylated alanine rich protein kinase C substrate	0.008	1.28
MT1X	Metallothionein 1X	0.008	-3.13

**Table 2:** Top 20 candidate gastric cancer genes in the CCGD database ranked by the number of supporting studies.

Gene ID	Number of studies
PTEN	45
CREBBP	32
DYRK1A	28
GSK3B	28
KDM6A	27
WAC	26
ZMIZ1	26
NF1	25
SETD5	25
PICALM	24
RAF1	24
PPP1R12A	23
SFI1	23
ERBB2IP	22
PPP6R3	22
ANKRD11	21
CTNNA1	21
TAOK1	21
KANSL1	20
PUM1	20

**Table 3:** 36 link genes shared by *F. nucleatum*-stimulated DEGs in neutrophils and gastric cancer candidate genes.

Gene ID	Gene	Adjusted *p* value^∗^	Log fold change^∗^
DNAJB1	DnaJ heat shock protein family (Hsp40) member B1	0.003	-2.13
EHD1	EH domain-containing 1	0.011	1.16
IER2	Immediate early response 2	0.016	-0.75
SMARCE1	SWI/SNF-related, matrix-associated, actin-dependent regulator of chromatin, subfamily e, member 1	0.016	-3.25
GRIK1-AS2///BACH1	GRIK1 antisense RNA 2///BTB domain and CNC homolog 1	0.016	1.17
RAB5A	RAB5A, member RAS oncogene family	0.018	0.76
RYBP	RING1 and YY1 binding protein	0.022	-0.81
P4HB	Prolyl 4-hydroxylase subunit beta	0.022	-0.99
UQCR11	Ubiquinol-cytochrome c reductase, complex III subunit XI	0.022	-0.96
HSPE1	Heat shock protein family E (Hsp10) member 1	0.026	-1.42
ATP5J	ATP synthase, H+ transporting, mitochondrial Fo complex subunit F6	0.027	-1.07
RRAGC	Ras-related GTP binding C	0.027	-0.67
ARFIP1	ADP ribosylation factor interacting protein 1	0.028	1.06
B3GALT2	Beta-1,3-galactosyltransferase 2	0.028	-3.68
UBE2H	Ubiquitin conjugating enzyme E2 H	0.030	0.90
GNB1	G protein subunit beta 1	0.034	0.62
SETD5	SET domain-containing 5	0.037	0.74
GALR2	Galanin receptor 2	0.039	-2.76
TNPO3	Transportin 3	0.039	-2.70
TM9SF2	Transmembrane 9 superfamily member 2	0.039	-0.73
UBR4	Ubiquitin protein ligase E3 component n-recognin 4	0.040	0.69
CANX	Calnexin	0.041	0.69
WNK1	WNK lysine deficient protein kinase 1	0.042	-0.83
BMPR2	Bone morphogenetic protein receptor type 2	0.043	-3.06
DICER1	Dicer 1, ribonuclease III	0.043	-0.71
ARGLU1	Arginine and glutamate rich 1	0.046	-0.84
MOAP1	Modulator of apoptosis 1	0.046	-1.43
AFTPH	Aftiphilin	0.046	0.62
GARS	Glycyl-tRNA synthetase	0.047	-0.75
RABGAP1L	RAB GTPase activating protein 1-like	0.049	-0.95
SHB	SH2 domain-containing adaptor protein B	0.049	2.33
PBX1	PBX homeobox 1	0.049	-2.33
PCM1	Pericentriolar material 1	0.050	-2.29
GMFG	Glia maturation factor gamma	0.050	-0.45
TRAF3	TNF receptor-associated factor 3	0.050	0.93
LYN	LYN protooncogene, Src family tyrosine kinase	0.050	0.46

^∗^Genes are ranked by adjusted *p* values for *F. nucleatum*-stimulated DEGs in neutrophils.

**Table 4:** STRING functional enrichment analysis of 36 link gene PPI network^∗^.

Category	Term ID	Term description	Strength	False discovery rate
Compartments	GOCC:0070062	Extracellular exosome	0.95	0.012
	GOCC:0043230	Extracellular organelle	0.93	0.005
	GOCC:1903561	Extracellular vesicle	0.93	0.005
	GOCC:0098796	Membrane protein complex	0.61	0.018
	GOCC:0031982	Vesicle	0.54	0.010
	GOCC:0016020	Membrane	0.33	0.012
	GOCC:0043231	Intracellular membrane-bounded organelle	0.31	0.002
	GOCC:0043227	Membrane-bounded organelle	0.27	0.002
	GOCC:0005737	Cytoplasm	0.27	0.006
	GOCC:0043226	Organelle	0.25	0.002
	GOCC:0043229	Intracellular organelle	0.25	0.003
	GOCC:0005622	Intracellular	0.22	0.002
	GOCC:0110165	Cellular anatomical entity	0.15	0.002
	GO:0098805	Whole membrane	0.54	0.048
	GO:0031982	Vesicle	0.38	0.048
GO component	GO:0043231	Intracellular membrane-bounded organelle	0.21	0.020
	GO:0005737	Cytoplasm	0.2	0.020
	GO:0043227	Membrane-bounded organelle	0.17	0.020
	GO:0043229	Intracellular organelle	0.17	0.020
	GO:0043226	Organelle	0.15	0.020
	GO:0005622	Intracellular	0.12	0.048
Tissues	BTO:0000132	Blood platelet	0.91	0.048
	BTO:0000580	Blood cancer cell	0.77	0.001
	BTO:0001271	Leukemia cell	0.76	0.004
	BTO:0000345	Digestive gland	0.46	0.021
	BTO:0000142	Brain	0.36	0.004
	BTO:0001491	Viscus	0.34	0.021
	BTO:0000282	Head	0.31	0.007
	BTO:0000083	Female reproductive system	0.31	0.016
	BTO:0003091	Urogenital system	0.29	0.015
	BTO:0001489	Whole body	0.18	0.003
	BTO:0000042	Animal	0.12	0.013

^∗^The functional terms in each category are ranked by strength of enrichment.

## Data Availability

The datasets used and/or analyzed during the current study are available from the corresponding author on reasonable request.

## Supplementary Materials

Supplementary Materials Table S1: list of significant DEGs in the gene expression dataset GEO20151. Table S2: 886 annotated candidate gastric cancer human genes identified in the CGGD database. Table S3: functional enrichment analysis results from “G:profiler.”
