# Supplementary material for: Neutrophil Transcriptional Deregulation by the Periodontal Pathogen Fusobacterium nucleatum in Gastric Cancer: A Bioinformatic Study
Source: Dis Markers. 2022 Aug 18;2022:9584507. doi: 10.1155/2022/9584507 (PMC9410804; doi:10.1155/2022/9584507)
Supplement: Supplementary Materials — Table S1: list of significant DEGs in the gene expression dataset GEO20151. Table S2: 886 annotated candidate gastric cancer human genes identified in the CGGD database. Table S3: functional enrichment analysis results from “G:profiler.” [file 9584507.f1.docx]

Table S1: List of Significant DEGs in the gene expression dataset GEO20151

| ID | adj.P.Val | P.Value | t | B | logFC | Gene.symbol | Gene.title |  |
| --- | --- | --- | --- | --- | --- | --- | --- | --- |
| 210678_s_at | 0.031 | 0.000 | -6.530 | -0.151 | -3.020 | AGPAT2 | 1-acylglycerol-3-phosphate O-acyltransferase 2 | |
| 219664_s_at | 0.028 | 0.000 | -6.750 | -0.135 | -2.730 | DECR2 | 2,4-dienoyl-CoA reductase 2, peroxisomal | |
| 217753_s_at | 0.016 | 0.000 | -8.610 | 2.057 | -0.946 | LOC101929876///LOC100996747///RPS26P11///RPS26 | 40S ribosomal protein S26///40S ribosomal protein S26///ribosomal protein S26 pseudogene 11///ribosomal protein S26 | |
| 202464_s_at | 0.011 | 0.000 | -11.000 | 3.504 | -1.060 | PFKFB3 | 6-phosphofructo-2-kinase/fructose-2,6-biphosphatase 3 | |
| 212543_at | 0.041 | 0.001 | 5.870 | -0.176 | 0.966 | AIM1 | absent in melanoma 1 | |
| 208636_at | 0.028 | 0.000 | -6.780 | 0.481 | -0.610 | ACTN1 | actinin alpha 1 | |
| 200779_at | 0.035 | 0.001 | 6.280 | 0.052 | 1.080 | ATF4 | activating transcription factor 4 | |
| 207275_s_at | 0.016 | 0.000 | 8.900 | 2.105 | 0.793 | ACSL1 | acyl-CoA synthetase long-chain family member 1 | |
| 207992_s_at | 0.024 | 0.000 | 7.310 | 1.091 | 0.897 | AMPD3 | adenosine monophosphate deaminase 3 | |
| 207111_at | 0.050 | 0.001 | 5.420 | -0.680 | 0.658 | ADGRE1 | adhesion G protein-coupled receptor E1 | |
| 214483_s_at | 0.028 | 0.000 | 6.780 | 0.692 | 1.060 | ARFIP1 | ADP ribosylation factor interacting protein 1 | |
| 205020_s_at | 0.039 | 0.001 | -6.000 | -0.537 | -3.850 | ARL4A | ADP ribosylation factor like GTPase 4A | |
| 202912_at | 0.012 | 0.000 | 10.200 | 3.017 | 0.854 | ADM | adrenomedullin | |
| 217939_s_at | 0.046 | 0.001 | 5.580 | -0.589 | 0.615 | AFTPH | aftiphilin |  |
| 212609_s_at | 0.049 | 0.001 | 5.430 | -0.992 | 2.670 | AKT3 | AKT serine/threonine kinase 3 | |
| 212286_at | 0.040 | 0.001 | 5.920 | -0.078 | 1.040 | ANKRD12 | ankyrin repeat domain 12 | |
| 206200_s_at | 0.043 | 0.001 | -5.740 | -0.469 | -0.588 | ANXA11 | annexin A11 |  |
| 209584_x_at | 0.037 | 0.001 | -6.170 | -0.473 | -2.720 | APOBEC3C | apolipoprotein B mRNA editing enzyme catalytic subunit 3C | |
| 220023_at | 0.038 | 0.001 | 6.090 | 0.069 | 0.861 | APOBR | apolipoprotein B receptor | |
| 34206_at | 0.035 | 0.001 | 6.270 | 0.188 | 0.869 | ARAP1 | ArfGAP with RhoGAP domain, ankyrin repeat and PH domain 1 | |
| 218067_s_at | 0.046 | 0.001 | -5.600 | -0.405 | -0.842 | ARGLU1 | arginine and glutamate rich 1 | |
| 209007_s_at | 0.034 | 0.001 | 6.350 | 0.096 | 0.635 | RSRP1 | arginine and serine rich protein 1 | |
| 212723_at | 0.016 | 0.000 | -9.010 | 2.316 | -1.370 | JMJD6 | arginine demethylase and lysine hydroxylase | |
| 212722_s_at | 0.020 | 0.000 | -7.930 | 1.602 | -1.150 | JMJD6 | arginine demethylase and lysine hydroxylase | |
| 217858_s_at | 0.023 | 0.000 | -7.490 | 1.281 | -1.240 | ARMCX3 | armadillo repeat containing, X-linked 3 | |
| 217801_at | 0.047 | 0.001 | -5.540 | -0.789 | -0.653 | ATP5E | ATP synthase, H+ transporting, mitochondrial F1 complex, epsilon subunit | |
| 202325_s_at | 0.027 | 0.000 | -6.950 | 0.853 | -1.070 | ATP5J | ATP synthase, H+ transporting, mitochondrial Fo complex subunit F6 | |
| 208746_x_at | 0.016 | 0.000 | -8.680 | 2.085 | -0.994 | ATP5L | ATP synthase, H+ transporting, mitochondrial Fo complex subunit G | |
| 210453_x_at | 0.021 | 0.000 | -7.750 | 1.378 | -0.804 | ATP5L | ATP synthase, H+ transporting, mitochondrial Fo complex subunit G | |
| 207573_x_at | 0.031 | 0.000 | -6.530 | 0.357 | -0.785 | ATP5L | ATP synthase, H+ transporting, mitochondrial Fo complex subunit G | |
| 219558_at | 0.037 | 0.001 | 6.170 | 0.205 | 1.580 | ATP13A3 | ATPase 13A3 | |
| 200954_at | 0.038 | 0.001 | -6.070 | -0.102 | -0.799 | ATP6V0C | ATPase H+ transporting V0 subunit c | |
| 220199_s_at | 0.027 | 0.000 | -6.900 | 0.083 | -3.740 | AIDA | axin interactor, dorsalization associated | |
| 205084_at | 0.020 | 0.000 | -7.870 | 0.116 | -3.420 | BCAP29 | B-cell receptor-associated protein 29 | |
| 200796_s_at | 0.047 | 0.001 | 5.530 | -0.463 | 0.780 | MCL1 | BCL2 family apoptosis regulator | |
| 217452_s_at | 0.028 | 0.000 | -6.750 | -0.263 | -3.680 | B3GALT2 | beta-1,3-galactosyltransferase 2 | |
| 221484_at | 0.011 | 0.000 | 10.900 | 3.479 | 1.090 | B4GALT5 | beta-1,4-galactosyltransferase 5 | |
| 221485_at | 0.032 | 0.001 | 6.460 | 0.185 | 0.705 | B4GALT5 | beta-1,4-galactosyltransferase 5 | |
| 220507_s_at | 0.046 | 0.001 | 5.600 | -0.346 | 0.933 | UPB1 | beta-ureidopropionase 1 | |
| 211725_s_at | 0.016 | 0.000 | 8.490 | 1.818 | 0.675 | BID | BH3 interacting domain death agonist | |
| 204493_at | 0.024 | 0.000 | 7.290 | 0.948 | 1.040 | BID | BH3 interacting domain death agonist | |
| 210214_s_at | 0.043 | 0.001 | -5.730 | -0.751 | -3.060 | BMPR2 | bone morphogenetic protein receptor type 2 | |
| 203053_at | 0.026 | 0.000 | -7.030 | 0.834 | -0.836 | BCAS2 | breast carcinoma amplified sequence 2 | |
| 221623_at | 0.030 | 0.000 | 6.620 | -0.095 | 3.740 | BCAN | brevican |  |
| 217986_s_at | 0.044 | 0.001 | 5.700 | -0.466 | 0.763 | BAZ1A | bromodomain adjacent to zinc finger domain 1A | |
| 214911_s_at | 0.046 | 0.001 | 5.560 | -0.608 | 0.678 | BRD2 | bromodomain containing 2 | |
| 208686_s_at | 0.047 | 0.001 | 5.530 | -0.570 | 0.796 | BRD2 | bromodomain containing 2 | |
| 200920_s_at | 0.043 | 0.001 | 5.730 | -0.689 | 0.445 | BTG1 | BTG anti-proliferation factor 1 | |
| 201235_s_at | 0.008 | 0.000 | -14.000 | 4.967 | -1.400 | BTG2 | BTG anti-proliferation factor 2 | |
| 220421_at | 0.016 | 0.000 | 8.690 | 2.116 | 1.280 | BTNL8 | butyrophilin like 8 | |
| 205476_at | 0.015 | 0.000 | -9.600 | 2.664 | -1.760 | CCL20 | C-C motif chemokine ligand 20 | |
| 211434_s_at | 0.016 | 0.000 | -8.720 | 2.067 | -0.839 | CCRL2 | C-C motif chemokine receptor like 2 | |
| 202329_at | 0.040 | 0.001 | 5.900 | -0.200 | 0.709 | CSK | c-src tyrosine kinase | |
| 204470_at | 0.011 | 0.000 | -11.900 | 3.925 | -1.020 | CXCL1 | C-X-C motif chemokine ligand 1 | |
| 209774_x_at | 0.008 | 0.000 | -14.200 | 5.071 | -1.100 | CXCL2 | C-X-C motif chemokine ligand 2 | |
| 207850_at | 0.003 | 0.000 | -19.700 | 4.904 | -4.130 | CXCL3 | C-X-C motif chemokine ligand 3 | |
| 207094_at | 0.024 | 0.000 | -7.330 | 1.029 | -0.815 | CXCR1 | C-X-C motif chemokine receptor 1 | |
| 217028_at | 0.040 | 0.001 | -5.900 | -0.624 | -0.439 | CXCR4 | C-X-C motif chemokine receptor 4 | |
| 211469_s_at | 0.012 | 0.000 | 10.300 | 1.380 | 3.640 | CXCR6 | C-X-C motif chemokine receptor 6 | |
| 218610_s_at | 0.028 | 0.000 | -6.800 | 0.701 | -0.931 | CPPED1 | calcineurin like phosphoesterase domain containing 1 | |
| 206612_at | 0.047 | 0.001 | -5.530 | -0.884 | -2.440 | CACNG1 | calcium voltage-gated channel auxiliary subunit gamma 1 | |
| 212252_at | 0.027 | 0.000 | 6.900 | 0.736 | 0.821 | CAMKK2 | calcium/calmodulin dependent protein kinase kinase 2 | |
| 200068_s_at | 0.041 | 0.001 | 5.840 | -0.230 | 0.694 | CANX | calnexin |  |
| 212586_at | 0.032 | 0.000 | -6.500 | 0.464 | -1.100 | CAST | calpastatin |  |
| 206756_at | 0.040 | 0.001 | 5.910 | -0.084 | 1.040 | CHST7 | carbohydrate sulfotransferase 7 | |
| 206209_s_at | 0.047 | 0.001 | -5.510 | -0.408 | -1.040 | CA4 | carbonic anhydrase 4 | |
| 201942_s_at | 0.039 | 0.001 | 5.990 | -0.131 | 0.739 | CPD | carboxypeptidase D | |
| 217209_at | 0.024 | 0.000 | 7.310 | 1.139 | 1.230 | CEACAM3 | carcinoembryonic antigen related cell adhesion molecule 3 | |
| 210563_x_at | 0.028 | 0.000 | 6.860 | 0.541 | 0.726 | CFLAR | CASP8 and FADD like apoptosis regulator | |
| 211862_x_at | 0.037 | 0.001 | 6.150 | -0.062 | 0.739 | CFLAR | CASP8 and FADD like apoptosis regulator | |
| 210564_x_at | 0.039 | 0.001 | 5.950 | -0.180 | 0.894 | CFLAR | CASP8 and FADD like apoptosis regulator | |
| 213596_at | 0.016 | 0.000 | 9.010 | 2.339 | 1.240 | CASP4 | caspase 4 |  |
| 202901_x_at | 0.039 | 0.001 | -6.000 | -0.110 | -0.952 | CTSS | cathepsin S |  |
| 203065_s_at | 0.016 | 0.000 | -8.920 | 0.800 | -3.680 | CAV1 | caveolin 1 |  |
| 203973_s_at | 0.026 | 0.000 | -7.000 | 0.717 | -0.747 | CEBPD | CCAAT/enhancer binding protein delta | |
| 209835_x_at | 0.024 | 0.000 | 7.280 | 1.014 | 1.250 | CD44 | CD44 molecule (Indian blood group) | |
| 204490_s_at | 0.030 | 0.000 | 6.580 | 0.452 | 0.839 | CD44 | CD44 molecule (Indian blood group) | |
| 212063_at | 0.037 | 0.001 | 6.170 | -0.019 | 0.654 | CD44 | CD44 molecule (Indian blood group) | |
| 212014_x_at | 0.040 | 0.001 | 5.920 | -0.216 | 0.703 | CD44 | CD44 molecule (Indian blood group) | |
| 204118_at | 0.022 | 0.000 | 7.610 | 1.186 | 0.868 | CD48 | CD48 molecule | |
| 222061_at | 0.016 | 0.000 | 9.150 | 2.430 | 1.230 | CD58 | CD58 molecule | |
| 211744_s_at | 0.022 | 0.000 | 7.660 | 1.343 | 0.874 | CD58 | CD58 molecule | |
| 203904_x_at | 0.045 | 0.001 | 5.630 | -0.482 | 1.220 | CD82 | CD82 molecule | |
| 204440_at | 0.011 | 0.000 | -10.800 | 3.397 | -0.931 | CD83 | CD83 molecule | |
| 202878_s_at | 0.040 | 0.001 | 5.900 | -0.310 | 0.638 | CD93 | CD93 molecule | |
| 204170_s_at | 0.006 | 0.000 | -14.900 | 4.752 | -2.330 | CKS2 | CDC28 protein kinase regulatory subunit 2 | |
| 209850_s_at | 0.044 | 0.001 | 5.660 | -0.405 | 0.979 | CDC42EP2 | CDC42 effector protein 2 | |
| 209286_at | 0.043 | 0.001 | -5.720 | -0.509 | -0.546 | CDC42EP3 | CDC42 effector protein 3 | |
| 209288_s_at | 0.044 | 0.001 | -5.680 | -0.686 | -0.559 | CDC42EP3 | CDC42 effector protein 3 | |
| 208727_s_at | 0.039 | 0.001 | 6.060 | -0.193 | 0.820 | CDC42 | cell division cycle 42 | |
| 219082_at | 0.044 | 0.001 | -5.660 | -0.371 | -1.730 | CEMP1///AMDHD2 | cementum protein 1///amidohydrolase domain containing 2 | |
| 204074_s_at | 0.036 | 0.001 | -6.230 | -0.408 | -2.880 | CEP104 | centrosomal protein 104 | |
| 219242_at | 0.046 | 0.001 | -5.560 | -0.414 | -0.808 | CEP63 | centrosomal protein 63 | |
| 202536_at | 0.025 | 0.000 | -7.220 | 0.996 | -0.808 | CHMP2B | charged multivesicular body protein 2B | |
| 218571_s_at | 0.043 | 0.001 | -5.740 | -0.493 | -2.210 | CHMP4A///TM9SF1 | charged multivesicular body protein 4A///transmembrane 9 superfamily member 1 | |
| 218085_at | 0.039 | 0.001 | -6.040 | 0.021 | -0.882 | CHMP5 | charged multivesicular body protein 5 | |
| 221058_s_at | 0.026 | 0.000 | -6.990 | 0.824 | -0.934 | CKLF | chemokine like factor | |
| 210532_s_at | 0.036 | 0.001 | -6.240 | 0.132 | -0.721 | C14orf2 | chromosome 14 open reading frame 2 | |
| 220941_s_at | 0.016 | 0.000 | 8.570 | 1.988 | 1.510 | C21orf91 | chromosome 21 open reading frame 91 | |
| 222309_at | 0.030 | 0.000 | -6.600 | 0.581 | -1.580 | C6orf62 | chromosome 6 open reading frame 62 | |
| 218541_s_at | 0.040 | 0.001 | -5.880 | -0.293 | -2.250 | C8orf4 | chromosome 8 open reading frame 4 | |
| 217957_at | 0.012 | 0.000 | -10.100 | 2.923 | -1.710 | CFAP20 | cilia and flagella associated protein 20 | |
| 220469_at | 0.026 | 0.000 | -7.100 | 0.127 | -3.020 | COPE | coatomer protein complex subunit epsilon | |
| 209716_at | 0.008 | 0.000 | -13.500 | 4.634 | -2.120 | CSF1 | colony stimulating factor 1 | |
| 209674_at | 0.016 | 0.000 | -8.510 | 2.011 | -1.200 | CRY1 | cryptochrome circadian clock 1 | |
| 220277_at | 0.028 | 0.000 | -6.850 | -0.050 | -2.900 | CXXC4 | CXXC finger protein 4 | |
| 220046_s_at | 0.039 | 0.001 | -5.970 | -0.435 | -0.454 | CCNL1 | cyclin L1 |  |
| 221903_s_at | 0.011 | 0.000 | 11.000 | 3.504 | 1.090 | CYLD | CYLD lysine 63 deubiquitinase | |
| 213295_at | 0.046 | 0.001 | 5.570 | -0.502 | 0.666 | CYLD | CYLD lysine 63 deubiquitinase | |
| 39582_at | 0.047 | 0.001 | 5.550 | -0.418 | 0.805 | CYLD | CYLD lysine 63 deubiquitinase | |
| 212971_at | 0.047 | 0.001 | 5.530 | -0.609 | 0.694 | CARS | cysteinyl-tRNA synthetase | |
| 203922_s_at | 0.020 | 0.000 | 7.940 | 1.612 | 1.130 | CYBB | cytochrome b-245 beta chain | |
| 203923_s_at | 0.037 | 0.001 | 6.160 | 0.141 | 0.875 | CYBB | cytochrome b-245 beta chain | |
| 202343_x_at | 0.016 | 0.000 | -8.510 | 2.005 | -1.100 | COX5B | cytochrome c oxidase subunit 5B | |
| 200925_at | 0.034 | 0.001 | -6.320 | 0.148 | -0.728 | COX6A1 | cytochrome c oxidase subunit 6A1 | |
| 201441_at | 0.032 | 0.001 | -6.440 | 0.331 | -0.866 | COX6B1 | cytochrome c oxidase subunit 6B1 | |
| 200998_s_at | 0.020 | 0.000 | 7.890 | 1.474 | 0.796 | CKAP4 | cytoskeleton associated protein 4 | |
| 205000_at | 0.018 | 0.000 | -8.130 | 1.674 | -1.100 | DDX3Y | DEAD-box helicase 3, Y-linked | |
| 205001_s_at | 0.043 | 0.001 | -5.760 | -0.368 | -0.781 | DDX3Y | DEAD-box helicase 3, Y-linked | |
| 212561_at | 0.011 | 0.000 | 11.500 | 3.770 | 1.050 | DENND5A | DENN domain containing 5A | |
| 218854_at | 0.040 | 0.001 | 5.880 | -0.227 | 0.713 | DSE | dermatan sulfate epimerase | |
| 213229_at | 0.043 | 0.001 | -5.730 | -0.373 | -0.709 | DICER1 | dicer 1, ribonuclease III | |
| 202887_s_at | 0.016 | 0.000 | 8.910 | 2.193 | 0.890 | DDIT4 | DNA damage inducible transcript 4 | |
| 200881_s_at | 0.016 | 0.000 | 8.420 | 1.793 | 0.730 | DNAJA1 | DnaJ heat shock protein family (Hsp40) member A1 | |
| 200666_s_at | 0.003 | 0.000 | -22.900 | 7.606 | -2.190 | DNAJB1 | DnaJ heat shock protein family (Hsp40) member B1 | |
| 200664_s_at | 0.003 | 0.000 | -18.800 | 6.565 | -2.130 | DNAJB1 | DnaJ heat shock protein family (Hsp40) member B1 | |
| 202843_at | 0.016 | 0.000 | -8.630 | 1.999 | -1.610 | DNAJB9 | DnaJ heat shock protein family (Hsp40) member B9 | |
| 202842_s_at | 0.025 | 0.000 | -7.240 | 0.940 | -0.704 | DNAJB9 | DnaJ heat shock protein family (Hsp40) member B9 | |
| 213092_x_at | 0.024 | 0.000 | -7.400 | -0.149 | -3.490 | DNAJC9 | DnaJ heat shock protein family (Hsp40) member C9 | |
| 201044_x_at | 0.011 | 0.000 | -11.300 | 3.648 | -1.360 | DUSP1 | dual specificity phosphatase 1 | |
| 201041_s_at | 0.028 | 0.000 | -6.740 | 0.157 | -0.411 | DUSP1 | dual specificity phosphatase 1 | |
| 201536_at | 0.026 | 0.000 | -7.020 | 0.918 | -1.140 | DUSP3 | dual specificity phosphatase 3 | |
| 208893_s_at | 0.012 | 0.000 | 10.200 | 3.043 | 1.490 | DUSP6 | dual specificity phosphatase 6 | |
| 208892_s_at | 0.016 | 0.000 | 8.450 | 1.909 | 0.869 | DUSP6 | dual specificity phosphatase 6 | |
| 208891_at | 0.023 | 0.000 | 7.470 | 1.139 | 1.170 | DUSP6 | dual specificity phosphatase 6 | |
| 203590_at | 0.045 | 0.001 | -5.620 | -0.507 | -1.940 | DYNC1LI2 | dynein cytoplasmic 1 light intermediate chain 2 | |
| 200703_at | 0.040 | 0.001 | -5.940 | 0.008 | -1.550 | DYNLL1 | dynein light chain LC8-type 1 | |
| 201999_s_at | 0.024 | 0.000 | -7.390 | 1.174 | -0.975 | DYNLT1 | dynein light chain Tctex-type 1 | |
| 201693_s_at | 0.042 | 0.001 | -5.820 | -0.179 | -0.896 | EGR1 | early growth response 1 | |
| 206115_at | 0.020 | 0.000 | -7.950 | 1.533 | -0.851 | EGR3 | early growth response 3 | |
| 208298_at | 0.034 | 0.001 | 6.360 | -0.366 | 2.670 | EVI5 | ecotropic viral integration site 5 | |
| 201775_s_at | 0.039 | 0.001 | 5.980 | 0.024 | 1.050 | EFCAB14 | EF-hand calcium binding domain 14 | |
| 217992_s_at | 0.016 | 0.000 | 8.920 | 2.220 | 0.866 | EFHD2 | EF-hand domain family member D2 | |
| 221497_x_at | 0.025 | 0.000 | -7.180 | 1.034 | -0.998 | EGLN1 | egl-9 family hypoxia inducible factor 1 | |
| 222221_x_at | 0.011 | 0.000 | 11.500 | 3.808 | 1.160 | EHD1 | EH domain containing 1 | |
| 209038_s_at | 0.028 | 0.000 | 6.830 | 0.620 | 0.743 | EHD1 | EH domain containing 1 | |
| 208112_x_at | 0.049 | 0.001 | 5.450 | -0.855 | 1.000 | EHD1 | EH domain containing 1 | |
| 200043_at | 0.037 | 0.001 | -6.180 | 0.132 | -0.803 | ERH | enhancer of rudimentary homolog (Drosophila) | |
| 204143_s_at | 0.016 | 0.000 | -8.520 | 0.739 | -3.310 | ENOSF1 | enolase superfamily member 1 | |
| 202909_at | 0.015 | 0.000 | 9.490 | 2.651 | 1.560 | EPM2AIP1 | EPM2A interacting protein 1 | |
| 1487_at | 0.048 | 0.001 | 5.500 | -0.449 | 0.838 | ESRRA | estrogen related receptor alpha | |
| 212225_at | 0.016 | 0.000 | -8.440 | 1.899 | -2.440 | EIF1 | eukaryotic translation initiation factor 1 | |
| 204409_s_at | 0.025 | 0.000 | -7.140 | 0.971 | -1.140 | EIF1AY | eukaryotic translation initiation factor 1A, Y-linked | |
| 201303_at | 0.011 | 0.000 | -10.700 | 3.329 | -0.924 | EIF4A3 | eukaryotic translation initiation factor 4A3 | |
| 208775_at | 0.047 | 0.001 | -5.520 | -0.462 | -0.828 | XPO1 | exportin 1 |  |
| 221813_at | 0.042 | 0.001 | 5.800 | -0.128 | 1.010 | FBXO42 | F-box protein 42 | |
| 213506_at | 0.045 | 0.001 | -5.620 | -0.287 | -1.320 | F2RL1 | F2R like trypsin receptor 1 | |
| 221804_s_at | 0.016 | 0.000 | -8.950 | 2.301 | -1.290 | FAM45A///FAM45BP | family with sequence similarity 45 member A///family with sequence similarity 45, member A pseudogene | |
| 212106_at | 0.028 | 0.000 | -6.720 | -0.014 | -3.190 | FAF2 | Fas associated factor family member 2 | |
| 216252_x_at | 0.032 | 0.000 | 6.500 | 0.342 | 0.715 | FAS | Fas cell surface death receptor | |
| 204780_s_at | 0.035 | 0.001 | 6.280 | 0.039 | 0.823 | FAS | Fas cell surface death receptor | |
| 215719_x_at | 0.049 | 0.001 | 5.440 | -0.731 | 0.673 | FAS | Fas cell surface death receptor | |
| 211307_s_at | 0.043 | 0.001 | 5.760 | -0.369 | 0.638 | FCAR | Fc fragment of IgA receptor | |
| 211306_s_at | 0.048 | 0.001 | -5.490 | -0.397 | -1.060 | FCAR | Fc fragment of IgA receptor | |
| 218831_s_at | 0.037 | 0.001 | -6.120 | -0.003 | -0.691 | FCGRT | Fc fragment of IgG receptor and transporter | |
| 215567_at | 0.033 | 0.001 | -6.390 | 0.318 | -1.620 | FCF1 | FCF1 rRNA-processing protein | |
| 208438_s_at | 0.024 | 0.000 | 7.360 | 0.935 | 0.657 | FGR | FGR proto-oncogene, Src family tyrosine kinase | |
| 210142_x_at | 0.015 | 0.000 | 9.310 | 2.459 | 0.873 | FLOT1 | flotillin 1 |  |
| 208749_x_at | 0.032 | 0.000 | 6.510 | 0.218 | 0.727 | FLOT1 | flotillin 1 |  |
| 218530_at | 0.028 | 0.000 | 6.840 | 0.777 | 1.180 | FHOD1 | formin homology 2 domain containing 1 | |
| 210773_s_at | 0.012 | 0.000 | 10.300 | 3.080 | 0.992 | FPR2 | formyl peptide receptor 2 | |
| 218881_s_at | 0.025 | 0.000 | -7.140 | 0.981 | -0.928 | FOSL2 | FOS like 2, AP-1 transcription factor subunit | |
| 209189_at | 0.003 | 0.000 | -20.700 | 7.211 | -2.440 | FOS | Fos proto-oncogene, AP-1 transcription factor subunit | |
| 202768_at | 0.020 | 0.000 | -7.850 | 1.537 | -1.690 | FOSB | FosB proto-oncogene, AP-1 transcription factor subunit | |
| 221345_at | 0.008 | 0.000 | 13.300 | 4.696 | 1.480 | FFAR2 | free fatty acid receptor 2 | |
| 209892_at | 0.030 | 0.000 | 6.590 | 0.537 | 1.110 | FUT4 | fucosyltransferase 4 | |
| 212486_s_at | 0.038 | 0.001 | 6.110 | -0.495 | 3.130 | FYN | FYN proto-oncogene, Src family tyrosine kinase | |
| 200746_s_at | 0.034 | 0.001 | 6.340 | 0.090 | 0.621 | GNB1 | G protein subunit beta 1 | |
| 200744_s_at | 0.043 | 0.001 | 5.750 | -0.437 | 0.607 | GNB1 | G protein subunit beta 1 | |
| 210600_s_at | 0.046 | 0.001 | -5.610 | -0.568 | -2.460 | GRK4 | G protein-coupled receptor kinase 4 | |
| 208840_s_at | 0.024 | 0.000 | -7.280 | 1.112 | -1.040 | G3BP2 | G3BP stress granule assembly factor 2 | |
| 208841_s_at | 0.027 | 0.000 | -6.880 | 0.724 | -0.852 | G3BP2 | G3BP stress granule assembly factor 2 | |
| 211226_at | 0.039 | 0.001 | -6.060 | -0.651 | -2.760 | GALR2 | galanin receptor 2 | |
| 213705_at | 0.016 | 0.000 | -8.460 | 0.485 | -3.630 | GGCX | gamma-glutamyl carboxylase | |
| 213142_x_at | 0.026 | 0.000 | 7.080 | 0.960 | 1.170 | GSAP | gamma-secretase activating protein | |
| 204220_at | 0.050 | 0.001 | -5.410 | -0.958 | -0.447 | GMFG | glia maturation factor gamma | |
| 202382_s_at | 0.021 | 0.000 | -7.710 | 1.394 | -1.190 | GNPDA1 | glucosamine-6-phosphate deaminase 1 | |
| 214966_at | 0.042 | 0.001 | -5.820 | -0.870 | -2.840 | GRIK5 | glutamate ionotropic receptor kainate type subunit 5 | |
| 200648_s_at | 0.037 | 0.001 | -6.190 | -0.358 | -0.444 | GLUL | glutamate-ammonia ligase | |
| 217202_s_at | 0.048 | 0.001 | -5.470 | -1.058 | -0.383 | GLUL | glutamate-ammonia ligase | |
| 215977_x_at | 0.045 | 0.001 | 5.640 | -0.442 | 0.759 | GK | glycerol kinase | |
| 216316_x_at | 0.015 | 0.000 | 9.550 | 2.687 | 1.340 | GK6P | glycerol kinase 6 pseudogene | |
| 208693_s_at | 0.047 | 0.001 | -5.550 | -0.528 | -0.746 | GARS | glycyl-tRNA synthetase | |
| 210818_s_at | 0.016 | 0.000 | 8.690 | 2.131 | 1.170 | GRIK1-AS2///BACH1 | GRIK1 antisense RNA 2///BTB domain and CNC homolog 1 | |
| 204224_s_at | 0.040 | 0.001 | 5.890 | -0.358 | 0.794 | GCH1 | GTP cyclohydrolase 1 | |
| 202269_x_at | 0.028 | 0.000 | 6.840 | 0.598 | 0.741 | GBP1 | guanylate binding protein 1 | |
| 213911_s_at | 0.043 | 0.001 | -5.750 | -0.436 | -0.630 | H2AFZ | H2A histone family member Z | |
| 208579_x_at | 0.018 | 0.000 | -8.110 | 1.605 | -0.902 | H2BFS | H2B histone family member S | |
| 208018_s_at | 0.012 | 0.000 | 10.200 | 2.958 | 0.808 | HCK | HCK proto-oncogene, Src family tyrosine kinase | |
| 201145_at | 0.047 | 0.001 | -5.520 | -0.434 | -0.892 | HAX1 | HCLS1 associated protein X-1 | |
| 211969_at | 0.006 | 0.000 | -15.400 | 5.577 | -1.200 | HSP90AA1 | heat shock protein 90 alpha family class A member 1 | |
| 210211_s_at | 0.015 | 0.000 | -9.330 | 2.421 | -0.854 | HSP90AA1 | heat shock protein 90 alpha family class A member 1 | |
| 211968_s_at | 0.016 | 0.000 | -8.960 | 2.193 | -1.180 | HSP90AA1 | heat shock protein 90 alpha family class A member 1 | |
| 214328_s_at | 0.034 | 0.001 | -6.320 | -0.108 | -0.964 | HSP90AA1 | heat shock protein 90 alpha family class A member 1 | |
| 200064_at | 0.015 | 0.000 | -9.240 | 2.470 | -0.995 | HSP90AB1 | heat shock protein 90 alpha family class B member 1 | |
| 214359_s_at | 0.022 | 0.000 | -7.680 | 1.417 | -1.070 | HSP90AB1 | heat shock protein 90 alpha family class B member 1 | |
| 200800_s_at | 0.004 | 0.000 | -17.500 | 6.288 | -2.040 | HSPA1L///HSPA1B///HSPA1A | heat shock protein family A (Hsp70) member 1 like///heat shock protein family A (Hsp70) member 1B///heat shock protein family A (Hsp70) member 1A | |
| 202581_at | 0.003 | 0.000 | -21.000 | 7.121 | -2.280 | HSPA1B///HSPA1A | heat shock protein family A (Hsp70) member 1B///heat shock protein family A (Hsp70) member 1A | |
| 200799_at | 0.038 | 0.001 | -6.100 | -0.342 | -0.671 | HSPA1B///HSPA1A | heat shock protein family A (Hsp70) member 1B///heat shock protein family A (Hsp70) member 1A | |
| 117_at | 0.012 | 0.000 | -10.100 | 2.928 | -1.530 | HSPA6 | heat shock protein family A (Hsp70) member 6 | |
| 200691_s_at | 0.046 | 0.001 | -5.580 | -0.468 | -0.818 | HSPA9 | heat shock protein family A (Hsp70) member 9 | |
| 205133_s_at | 0.026 | 0.000 | -7.020 | 0.898 | -1.420 | HSPE1 | heat shock protein family E (Hsp10) member 1 | |
| 206976_s_at | 0.015 | 0.000 | -9.400 | 2.595 | -2.140 | HSPH1 | heat shock protein family H (Hsp110) member 1 | |
| 208744_x_at | 0.032 | 0.000 | -6.500 | -0.084 | -2.470 | HSPH1 | heat shock protein family H (Hsp110) member 1 | |
| 203665_at | 0.003 | 0.000 | -19.100 | 6.366 | -2.290 | HMOX1 | heme oxygenase 1 | |
| 217232_x_at | 0.023 | 0.000 | -7.530 | 1.082 | -0.633 | HBB | hemoglobin subunit beta | |
| 209116_x_at | 0.040 | 0.001 | -5.920 | -0.362 | -0.654 | HBB | hemoglobin subunit beta | |
| 211696_x_at | 0.050 | 0.001 | -5.420 | -0.988 | -0.553 | HBB | hemoglobin subunit beta | |
| 200896_x_at | 0.025 | 0.000 | 7.210 | 0.995 | 0.919 | HDGF | hepatoma-derived growth factor | |
| 203394_s_at | 0.018 | 0.000 | -8.140 | 1.752 | -1.240 | HES1 | hes family bHLH transcription factor 1 | |
| 211929_at | 0.034 | 0.001 | -6.360 | 0.262 | -0.884 | HNRNPA3 | heterogeneous nuclear ribonucleoprotein A3 | |
| 211930_at | 0.040 | 0.001 | -5.940 | -0.108 | -0.819 | HNRNPA3 | heterogeneous nuclear ribonucleoprotein A3 | |
| 221767_x_at | 0.044 | 0.001 | -5.660 | -0.234 | -1.550 | HDLBP | high density lipoprotein binding protein | |
| 214938_x_at | 0.046 | 0.001 | -5.570 | -0.789 | -0.456 | HMGB1 | high mobility group box 1 | |
| 208808_s_at | 0.028 | 0.000 | -6.770 | 0.548 | -0.916 | HMGB2 | high mobility group box 2 | |
| 209787_s_at | 0.042 | 0.001 | -5.830 | -0.162 | -0.836 | HMGN4 | high mobility group nucleosomal binding domain 4 | |
| 200943_at | 0.042 | 0.001 | -5.790 | -0.343 | -0.688 | HMGN1 | high mobility group nucleosome binding domain 1 | |
| 204111_at | 0.038 | 0.001 | 6.100 | -0.647 | 2.980 | HNMT | histamine N-methyltransferase | |
| 207982_at | 0.047 | 0.001 | -5.530 | -0.882 | -2.430 | HIST1H1T | histone cluster 1, H1t | |
| 215071_s_at | 0.026 | 0.000 | -7.100 | 0.635 | -0.556 | HIST1H2AC | histone cluster 1, H2ac | |
| 214469_at | 0.043 | 0.001 | -5.760 | -0.171 | -1.170 | HIST1H2AE | histone cluster 1, H2ae | |
| 214542_x_at | 0.039 | 0.001 | -5.980 | -0.087 | -1.990 | HIST1H2AH///HIST1H2AG///HIST1H2AM///HIST1H2AL///HIST1H2AK///HIST1H2AI | histone cluster 1, H2ah///histone cluster 1, H2ag///histone cluster 1, H2am///histone cluster 1, H2al///histone cluster 1, H2ak///histone cluster 1, H2ai | |
| 214481_at | 0.025 | 0.000 | -7.150 | 0.226 | -3.420 | HIST1H2AM | histone cluster 1, H2am | |
| 208547_at | 0.015 | 0.000 | -9.440 | 1.146 | -3.610 | HIST1H2BB | histone cluster 1, H2bb | |
| 208523_x_at | 0.026 | 0.000 | -6.980 | 0.804 | -0.808 | HIST1H2BC///HIST1H2BI///HIST1H2BE///HIST1H2BF///HIST1H2BG | histone cluster 1, H2bc///histone cluster 1, H2bi///histone cluster 1, H2be///histone cluster 1, H2bf///histone cluster 1, H2bg | |
| 208490_x_at | 0.028 | 0.000 | -6.770 | 0.512 | -0.735 | HIST1H2BC///HIST1H2BI///HIST1H2BE///HIST1H2BF///HIST1H2BG | histone cluster 1, H2bc///histone cluster 1, H2bi///histone cluster 1, H2be///histone cluster 1, H2bf///histone cluster 1, H2bg | |
| 209911_x_at | 0.039 | 0.001 | -6.000 | -0.134 | -0.852 | HIST1H2BD | histone cluster 1, H2bd | |
| 208527_x_at | 0.022 | 0.000 | -7.670 | 1.294 | -0.989 | HIST1H2BE | histone cluster 1, H2be | |
| 210387_at | 0.012 | 0.000 | -10.400 | 2.369 | -2.920 | HIST1H2BJ///HIST1H2BG | histone cluster 1, H2bj///histone cluster 1, H2bg | |
| 215779_s_at | 0.029 | 0.000 | -6.700 | 0.642 | -1.160 | HIST1H2BJ///HIST1H2BG | histone cluster 1, H2bj///histone cluster 1, H2bg | |
| 214522_x_at | 0.013 | 0.000 | -9.840 | 2.845 | -1.350 | HIST1H3D///HIST1H2AD | histone cluster 1, H3d///histone cluster 1, H2ad | |
| 214472_at | 0.020 | 0.000 | -7.900 | 1.570 | -1.170 | HIST1H3F///HIST1H3B///HIST1H3H///HIST1H3J///HIST1H3G///HIST1H3I///HIST1H3E///HIST1H3C///HIST1H3D///HIST1H3A///HIST1H2AD | histone cluster 1, H3f///histone cluster 1, H3b///histone cluster 1, H3h///histone cluster 1, H3j///histone cluster 1, H3g///histone cluster 1, H3i///histone cluster 1, H3e///histone cluster 1, H3c///histone cluster 1, H3d///histone cluster 1, H3a///histone cluster 1, H2ad | |
| 208496_x_at | 0.050 | 0.001 | -5.400 | -0.570 | -0.953 | HIST1H3G | histone cluster 1, H3g | |
| 212998_x_at | 0.034 | 0.001 | -6.300 | 0.321 | -1.270 | LOC101060835///HLA-DQB1 | HLA class II histocompatibility antigen, DQ beta 1 chain-like///major histocompatibility complex, class II, DQ beta 1 | |
| 204544_at | 0.039 | 0.001 | -5.990 | -0.003 | -0.907 | HPS5 | HPS5, biogenesis of lysosomal organelles complex 2 subunit 2 | |
| 204512_at | 0.045 | 0.001 | 5.630 | -0.439 | 0.744 | HIVEP1 | human immunodeficiency virus type I enhancer binding protein 1 | |
| 212642_s_at | 0.024 | 0.000 | 7.380 | 1.188 | 1.200 | HIVEP2 | human immunodeficiency virus type I enhancer binding protein 2 | |
| 218507_at | 0.008 | 0.000 | 12.900 | 4.259 | 1.950 | HILPDA | hypoxia inducible lipid droplet associated | |
| 210666_at | 0.027 | 0.000 | -6.890 | -0.059 | -2.830 | IDS | iduronate 2-sulfatase | |
| 202439_s_at | 0.042 | 0.001 | -5.810 | -0.198 | -0.875 | IDS | iduronate 2-sulfatase | |
| 212221_x_at | 0.043 | 0.001 | -5.760 | -0.414 | -0.778 | IDS | iduronate 2-sulfatase | |
| 202081_at | 0.016 | 0.000 | -8.530 | 1.863 | -0.754 | IER2 | immediate early response 2 | |
| 221877_at | 0.028 | 0.000 | -6.840 | 0.137 | -2.550 | IRGQ | immunity related GTPase Q | |
| 206420_at | 0.046 | 0.001 | 5.610 | -0.681 | 0.564 | IGSF6 | immunoglobulin superfamily member 6 | |
| 201362_at | 0.027 | 0.000 | 6.910 | 0.517 | 0.593 | IVNS1ABP | influenza virus NS1A binding protein | |
| 215086_at | 0.033 | 0.001 | 6.420 | -0.464 | 3.030 | IBTK | inhibitor of Bruton tyrosine kinase | |
| 201566_x_at | 0.011 | 0.000 | -11.500 | 3.699 | -1.980 | ID2 | inhibitor of DNA binding 2, HLH protein | |
| 201565_s_at | 0.014 | 0.000 | -9.770 | 2.775 | -1.180 | ID2 | inhibitor of DNA binding 2, HLH protein | |
| 201627_s_at | 0.030 | 0.000 | -6.610 | 0.437 | -0.730 | INSIG1 | insulin induced gene 1 | |
| 209185_s_at | 0.011 | 0.000 | -11.300 | 3.699 | -1.140 | IRS2 | insulin receptor substrate 2 | |
| 209184_s_at | 0.024 | 0.000 | -7.410 | 1.129 | -0.835 | IRS2 | insulin receptor substrate 2 | |
| 214660_at | 0.038 | 0.001 | -6.090 | -0.286 | -3.790 | ITGA1 | integrin subunit alpha 1 | |
| 213620_s_at | 0.039 | 0.001 | 5.970 | 0.011 | 1.220 | ICAM2 | intercellular adhesion molecule 2 | |
| 201642_at | 0.027 | 0.000 | 6.950 | 0.473 | 0.674 | IFNGR2 | interferon gamma receptor 2 (interferon gamma transducer 1) | |
| 219209_at | 0.016 | 0.000 | 8.550 | 2.040 | 1.070 | IFIH1 | interferon induced with helicase C domain 1 | |
| 205067_at | 0.016 | 0.000 | 8.450 | 1.681 | 0.635 | IL1B | interleukin 1 beta | |
| 39402_at | 0.050 | 0.001 | 5.410 | -1.058 | 0.410 | IL1B | interleukin 1 beta | |
| 216243_s_at | 0.008 | 0.000 | 13.200 | 4.625 | 1.030 | IL1RN | interleukin 1 receptor antagonist | |
| 212659_s_at | 0.015 | 0.000 | 9.300 | 2.375 | 1.210 | IL1RN | interleukin 1 receptor antagonist | |
| 204912_at | 0.016 | 0.000 | 8.550 | 2.013 | 0.939 | IL10RA | interleukin 10 receptor subunit alpha | |
| 209827_s_at | 0.034 | 0.001 | 6.300 | 0.210 | 0.765 | IL16 | interleukin 16 | |
| 220054_at | 0.026 | 0.000 | 7.020 | 0.867 | 1.450 | IL23A | interleukin 23 subunit alpha | |
| 205483_s_at | 0.028 | 0.000 | -6.780 | 0.602 | -0.747 | ISG15 | ISG15 ubiquitin-like modifier | |
| 201751_at | 0.049 | 0.001 | -5.430 | -0.724 | -0.796 | JOSD1 | Josephin domain containing 1 | |
| 201464_x_at | 0.011 | 0.000 | -11.100 | 3.503 | -2.190 | JUN | Jun proto-oncogene, AP-1 transcription factor subunit | |
| 201465_s_at | 0.043 | 0.001 | -5.760 | -0.679 | -3.180 | JUN | Jun proto-oncogene, AP-1 transcription factor subunit | |
| 201473_at | 0.040 | 0.001 | -5.910 | -0.407 | -0.606 | JUNB | JunB proto-oncogene, AP-1 transcription factor subunit | |
| 203068_at | 0.012 | 0.000 | -10.400 | 3.178 | -1.300 | KLHL21 | kelch like family member 21 | |
| 219106_s_at | 0.046 | 0.001 | 5.580 | -0.865 | 3.860 | KLHL41 | kelch like family member 41 | |
| 218963_s_at | 0.032 | 0.000 | 6.470 | 0.245 | 0.872 | KRT23 | keratin 23 |  |
| 202393_s_at | 0.015 | 0.000 | -9.400 | 2.509 | -0.820 | KLF10 | Kruppel like factor 10 | |
| 219371_s_at | 0.028 | 0.000 | -6.800 | 0.495 | -0.818 | KLF2 | Kruppel like factor 2 | |
| 208961_s_at | 0.016 | 0.000 | -8.650 | 2.061 | -0.877 | KLF6 | Kruppel like factor 6 | |
| 208960_s_at | 0.048 | 0.001 | -5.470 | -0.648 | -0.626 | KLF6 | Kruppel like factor 6 | |
| 208949_s_at | 0.034 | 0.001 | -6.330 | -0.036 | -0.617 | LGALS3 | lectin, galactoside binding soluble 3 | |
| 205266_at | 0.013 | 0.000 | -9.950 | 2.776 | -2.350 | LIF | leukemia inhibitory factor | |
| 202193_at | 0.014 | 0.000 | 9.800 | 2.791 | 0.955 | LIMK2 | LIM domain kinase 2 | |
| 210582_s_at | 0.037 | 0.001 | 6.150 | -0.107 | 1.080 | LIMK2 | LIM domain kinase 2 | |
| 205571_at | 0.047 | 0.001 | -5.550 | -0.338 | -1.190 | LIPT1 | lipoyltransferase 1 | |
| 204559_s_at | 0.039 | 0.001 | -5.990 | -0.601 | -2.600 | LSM7 | LSM7 homolog, U6 small nuclear RNA and mRNA degradation associated | |
| 206584_at | 0.047 | 0.001 | -5.510 | -0.787 | -0.582 | LY96 | lymphocyte antigen 96 | |
| 208885_at | 0.049 | 0.001 | 5.450 | -1.032 | 0.397 | LCP1 | lymphocyte cytosolic protein 1 | |
| 207339_s_at | 0.029 | 0.000 | 6.710 | 0.431 | 0.671 | LTB | lymphotoxin beta | |
| 210754_s_at | 0.050 | 0.001 | 5.400 | -1.059 | 0.455 | LYN | LYN proto-oncogene, Src family tyrosine kinase | |
| 209192_x_at | 0.043 | 0.001 | 5.710 | -0.242 | 0.914 | KAT5 | lysine acetyltransferase 5 | |
| 212492_s_at | 0.048 | 0.001 | -5.470 | -0.578 | -0.727 | KDM4B | lysine demethylase 4B | |
| 205193_at | 0.011 | 0.000 | -10.900 | 3.476 | -1.230 | MAFF | MAF bZIP transcription factor F | |
| 36711_at | 0.016 | 0.000 | -8.370 | 1.675 | -0.756 | MAFF | MAF bZIP transcription factor F | |
| 204970_s_at | 0.016 | 0.000 | -9.030 | 2.348 | -1.240 | MAFG | MAF bZIP transcription factor G | |
| 220178_at | 0.016 | 0.000 | -8.660 | 0.987 | -3.790 | MFSD12 | major facilitator superfamily domain containing 12 | |
| 211911_x_at | 0.032 | 0.001 | 6.440 | -0.209 | 0.364 | HLA-B | major histocompatibility complex, class I, B | |
| 209728_at | 0.012 | 0.000 | 10.500 | 2.978 | 1.960 | HLA-DRB4 | major histocompatibility complex, class II, DR beta 4 | |
| 212708_at | 0.045 | 0.001 | -5.630 | -0.475 | -0.655 | MSL1 | male specific lethal 1 homolog | |
| 217652_at | 0.028 | 0.000 | -6.830 | -0.086 | -2.740 | MAU2 | MAU2 sister chromatid cohesion factor | |
| 221545_x_at | 0.048 | 0.001 | -5.490 | -0.901 | -2.340 | MED16 | mediator complex subunit 16 | |
| 207496_at | 0.049 | 0.001 | 5.460 | -0.863 | 2.750 | MS4A2 | membrane spanning 4-domains A2 | |
| 217165_x_at | 0.044 | 0.001 | -5.680 | -0.731 | -2.980 | MT1F | metallothionein 1F | |
| 204745_x_at | 0.039 | 0.001 | -5.970 | -0.063 | -0.910 | MT1G | metallothionein 1G | |
| 206461_x_at | 0.025 | 0.000 | -7.170 | 1.022 | -1.890 | MT1H | metallothionein 1H | |
| 204326_x_at | 0.008 | 0.000 | -13.400 | 3.450 | -3.130 | MT1X | metallothionein 1X | |
| 208581_x_at | 0.012 | 0.000 | -10.200 | 2.721 | -2.520 | MT1X | metallothionein 1X | |
| 212185_x_at | 0.011 | 0.000 | -11.800 | 3.955 | -1.410 | MT2A | metallothionein 2A | |
| 219807_x_at | 0.046 | 0.001 | -5.600 | -0.299 | -1.100 | MIA-RAB4B///RAB4B | MIA-RAB4B readthrough (NMD candidate)///RAB4B, member RAS oncogene family | |
| 214696_at | 0.024 | 0.000 | -7.290 | 1.000 | -0.820 | MIR22///MIR22HG | microRNA 22///MIR22 host gene | |
| 214657_s_at | 0.005 | 0.000 | -15.600 | 5.615 | -1.510 | MIR612///NEAT1 | microRNA 612///nuclear paraspeckle assembly transcript 1 (non-protein coding) | |
| 214882_s_at | 0.037 | 0.001 | -6.180 | 0.037 | -0.867 | MIR636///SRSF2 | microRNA 636///serine and arginine rich splicing factor 2 | |
| 204908_s_at | 0.011 | 0.000 | 11.700 | 3.864 | 0.998 | MIR8085///BCL3 | microRNA 8085///B-cell CLL/lymphoma 3 | |
| 204907_s_at | 0.041 | 0.001 | 5.840 | -0.208 | 0.791 | MIR8085///BCL3 | microRNA 8085///B-cell CLL/lymphoma 3 | |
| 218251_at | 0.022 | 0.000 | 7.680 | 1.347 | 0.830 | MID1IP1 | MID1 interacting protein 1 | |
| 216237_s_at | 0.037 | 0.001 | -6.180 | -0.401 | -2.790 | MCM5 | minichromosome maintenance complex component 5 | |
| 218993_at | 0.043 | 0.001 | -5.710 | -0.772 | -2.420 | MRM3 | mitochondrial rRNA methyltransferase 3 | |
| 207121_s_at | 0.011 | 0.000 | -11.000 | 3.500 | -0.895 | MAPK6 | mitogen-activated protein kinase 6 | |
| 203652_at | 0.024 | 0.000 | 7.310 | 1.084 | 1.000 | MAP3K11 | mitogen-activated protein kinase kinase kinase 11 | |
| 211081_s_at | 0.042 | 0.001 | 5.790 | -0.788 | 3.430 | MAP4K5 | mitogen-activated protein kinase kinase kinase kinase 5 | |
| 201297_s_at | 0.042 | 0.001 | 5.810 | -0.192 | 0.865 | MOB1A | MOB kinase activator 1A | |
| 212508_at | 0.046 | 0.001 | -5.590 | -0.303 | -1.430 | MOAP1 | modulator of apoptosis 1 | |
| 211026_s_at | 0.044 | 0.001 | 5.690 | -0.295 | 0.835 | MGLL | monoglyceride lipase | |
| 202520_s_at | 0.033 | 0.001 | 6.380 | 0.343 | 1.040 | MLH1 | mutL homolog 1 | |
| 201319_at | 0.037 | 0.001 | -6.200 | 0.061 | -0.922 | MYL12A | myosin light chain 12A | |
| 201668_x_at | 0.008 | 0.000 | 12.800 | 4.450 | 1.280 | MARCKS | myristoylated alanine rich protein kinase C substrate | |
| 201670_s_at | 0.016 | 0.000 | 8.810 | 2.021 | 0.691 | MARCKS | myristoylated alanine rich protein kinase C substrate | |
| 215159_s_at | 0.018 | 0.000 | 8.250 | 1.828 | 1.320 | NADK | NAD kinase |  |
| 213607_x_at | 0.029 | 0.000 | 6.680 | 0.508 | 0.785 | NADK | NAD kinase |  |
| 208917_x_at | 0.050 | 0.001 | 5.400 | -0.680 | 0.943 | NADK | NAD kinase |  |
| 202298_at | 0.039 | 0.001 | -5.990 | -0.128 | -0.860 | NDUFA1 | NADH:ubiquinone oxidoreductase subunit A1 | |
| 202001_s_at | 0.034 | 0.001 | -6.300 | 0.313 | -1.010 | NDUFA6 | NADH:ubiquinone oxidoreductase subunit A6 | |
| 206790_s_at | 0.022 | 0.000 | -7.610 | 1.317 | -1.100 | NDUFB1 | NADH:ubiquinone oxidoreductase subunit B1 | |
| 221867_at | 0.012 | 0.000 | 10.400 | 3.107 | 1.490 | N4BP1 | NEDD4 binding protein 1 | |
| 204601_at | 0.016 | 0.000 | 8.700 | 2.042 | 1.400 | N4BP1 | NEDD4 binding protein 1 | |
| 202150_s_at | 0.011 | 0.000 | 11.300 | 3.646 | 1.530 | NEDD9 | neural precursor cell expressed, developmentally down-regulated 9 | |
| 218086_at | 0.027 | 0.000 | -6.960 | 0.018 | -2.920 | NPDC1 | neural proliferation, differentiation and control 1 | |
| 208926_at | 0.018 | 0.000 | -8.190 | 1.785 | -1.120 | NEU1 | neuraminidase 1 (lysosomal sialidase) | |
| 213612_x_at | 0.040 | 0.001 | 5.940 | -0.452 | 0.565 | NBPF20///NBPF10///NBPF8///NBPF9///NBPF15///NBPF12///NBPF14 | neuroblastoma breakpoint family member 20///neuroblastoma breakpoint family member 10///neuroblastoma breakpoint family member 8///neuroblastoma breakpoint family member 9///neuroblastoma breakpoint family member 15///neuroblastoma breakpoint family member 12///neuroblastoma breakpoint family member 14 | |
| 201103_x_at | 0.043 | 0.001 | 5.740 | -0.627 | 0.590 | NBPF20///NBPF10///NBPF8///NBPF9///NBPF15///NBPF11///NBPF12///NBPF14 | neuroblastoma breakpoint family member 20///neuroblastoma breakpoint family member 10///neuroblastoma breakpoint family member 8///neuroblastoma breakpoint family member 9///neuroblastoma breakpoint family member 15///neuroblastoma breakpoint family member 11///neuroblastoma breakpoint family member 12///neuroblastoma breakpoint family member 14 | |
| 201104_x_at | 0.016 | 0.000 | 9.060 | 2.295 | 0.824 | NBPF25P///NBPF26///NBPF19///NBPF20///NBPF10///NBPF8///NBPF9///NBPF15///NBPF11///NBPF14 | neuroblastoma breakpoint family member 25, pseudogene///neuroblastoma breakpoint family member 26///neuroblastoma breakpoint family member 19///neuroblastoma breakpoint family member 20///neuroblastoma breakpoint family member 10///neuroblastoma breakpoint family member 8///neuroblastoma breakpoint family member 9///neuroblastoma breakpoint family member 15///neuroblastoma breakpoint family member 11///neuroblastoma breakpoint family member 14 | |
| 214693_x_at | 0.015 | 0.000 | 9.250 | 2.492 | 1.040 | NBPF26///NBPF10///NBPF9///NBPF14 | neuroblastoma breakpoint family member 26///neuroblastoma breakpoint family member 10///neuroblastoma breakpoint family member 9///neuroblastoma breakpoint family member 14 | |
| 204961_s_at | 0.031 | 0.000 | 6.530 | 0.227 | 0.658 | NCF1C///NCF1B///NCF1 | neutrophil cytosolic factor 1C pseudogene///neutrophil cytosolic factor 1B pseudogene///neutrophil cytosolic factor 1 | |
| 203927_at | 0.016 | 0.000 | 8.970 | 2.313 | 1.300 | NFKBIE | NFKB inhibitor epsilon | |
| 209272_at | 0.021 | 0.000 | 7.720 | 1.425 | 1.020 | NAB1 | NGFI-A binding protein 1 | |
| 211139_s_at | 0.025 | 0.000 | 7.170 | 0.983 | 0.897 | NAB1 | NGFI-A binding protein 1 | |
| 202905_x_at | 0.011 | 0.000 | 10.700 | 3.352 | 1.040 | NBN | nibrin |  |
| 202906_s_at | 0.032 | 0.001 | 6.470 | 0.031 | 0.885 | NBN | nibrin |  |
| 217299_s_at | 0.039 | 0.001 | 6.020 | -0.159 | 0.999 | NBN | nibrin |  |
| 203045_at | 0.012 | 0.000 | 10.600 | 3.250 | 0.930 | NINJ1 | ninjurin 1 |  |
| 214722_at | 0.027 | 0.000 | 6.880 | 0.394 | 1.140 | NOTCH2NL | notch 2 N-terminal like | |
| 217831_s_at | 0.047 | 0.001 | -5.510 | -0.377 | -1.570 | NSFL1C | NSFL1 cofactor | |
| 209289_at | 0.040 | 0.001 | 5.880 | -0.603 | 2.800 | NFIB | nuclear factor I B | |
| 209239_at | 0.025 | 0.000 | 7.180 | 0.888 | 0.912 | NFKB1 | nuclear factor kappa B subunit 1 | |
| 207535_s_at | 0.028 | 0.000 | 6.750 | 0.532 | 1.200 | NFKB2 | nuclear factor kappa B subunit 2 | |
| 209636_at | 0.043 | 0.001 | 5.760 | -0.265 | 0.881 | NFKB2 | nuclear factor kappa B subunit 2 | |
| 207760_s_at | 0.045 | 0.001 | 5.620 | -0.337 | 0.908 | NCOR2 | nuclear receptor corepressor 2 | |
| 219557_s_at | 0.039 | 0.001 | -6.010 | 0.022 | -0.886 | NRIP3 | nuclear receptor interacting protein 3 | |
| 202340_x_at | 0.016 | 0.000 | -8.620 | 1.662 | -2.400 | NR4A1 | nuclear receptor subfamily 4 group A member 1 | |
| 216248_s_at | 0.027 | 0.000 | -6.880 | 0.753 | -0.907 | NR4A2 | nuclear receptor subfamily 4 group A member 2 | |
| 204621_s_at | 0.036 | 0.001 | -6.240 | 0.252 | -1.180 | NR4A2 | nuclear receptor subfamily 4 group A member 2 | |
| 209959_at | 0.026 | 0.000 | -7.070 | 0.939 | -2.550 | NR4A3 | nuclear receptor subfamily 4 group A member 3 | |
| 203194_s_at | 0.046 | 0.001 | 5.600 | -0.442 | 0.721 | NUP98 | nucleoporin 98 | |
| 212768_s_at | 0.035 | 0.001 | -6.250 | -0.330 | -3.450 | OLFM4 | olfactomedin 4 | |
| 206323_x_at | 0.016 | 0.000 | -9.130 | 2.403 | -0.990 | OPHN1 | oligophrenin 1 | |
| 214637_at | 0.004 | 0.000 | -16.500 | 5.469 | -2.340 | OSM | oncostatin M | |
| 202074_s_at | 0.048 | 0.001 | -5.500 | -0.518 | -1.870 | OPTN | optineurin |  |
| 200790_at | 0.008 | 0.000 | -13.100 | 3.762 | -2.780 | ODC1 | ornithine decarboxylase 1 | |
| 201365_at | 0.022 | 0.000 | 7.590 | 1.267 | 0.794 | OAZ2 | ornithine decarboxylase antizyme 2 | |
| 205040_at | 0.012 | 0.000 | -10.000 | 2.979 | -1.430 | ORM1 | orosomucoid 1 | |
| 205041_s_at | 0.015 | 0.000 | -9.450 | 2.596 | -1.040 | ORM2///ORM1 | orosomucoid 2///orosomucoid 1 | |
| 211697_x_at | 0.038 | 0.001 | -6.090 | -0.139 | -2.090 | PNO1 | partner of NOB1 homolog | |
| 212151_at | 0.049 | 0.001 | -5.440 | -0.956 | -2.330 | PBX1 | PBX homeobox 1 | |
| 214175_x_at | 0.039 | 0.001 | -6.040 | -0.480 | -2.820 | PDLIM4 | PDZ and LIM domain 4 | |
| 203691_at | 0.032 | 0.000 | 6.490 | -0.057 | 0.513 | PI3 | peptidase inhibitor 3 | |
| 201682_at | 0.038 | 0.001 | -6.080 | -0.142 | -2.000 | PMPCB | peptidase, mitochondrial processing beta subunit | |
| 202336_s_at | 0.016 | 0.000 | -8.410 | 0.743 | -3.270 | PAM | peptidylglycine alpha-amidating monooxygenase | |
| 202174_s_at | 0.050 | 0.001 | -5.420 | -0.877 | -2.290 | PCM1 | pericentriolar material 1 | |
| 209122_at | 0.016 | 0.000 | -8.520 | 2.015 | -1.450 | PLIN2 | perilipin 2 |  |
| 214981_at | 0.026 | 0.000 | -7.020 | -0.211 | -4.410 | POSTN | periostin |  |
| 213638_at | 0.017 | 0.000 | -8.280 | 1.698 | -0.701 | PHACTR1 | phosphatase and actin regulator 1 | |
| 203708_at | 0.015 | 0.000 | 9.250 | 2.424 | 0.838 | PDE4B | phosphodiesterase 4B | |
| 211302_s_at | 0.026 | 0.000 | 6.980 | 0.700 | 1.030 | PDE4B | phosphodiesterase 4B | |
| 211840_s_at | 0.021 | 0.000 | 7.780 | 0.483 | 3.620 | PDE4D | phosphodiesterase 4D | |
| 204091_at | 0.029 | 0.000 | -6.670 | -0.148 | -2.830 | PDE6D | phosphodiesterase 6D | |
| 220566_at | 0.026 | 0.000 | 7.050 | 0.882 | 0.908 | PIK3R5 | phosphoinositide-3-kinase regulatory subunit 5 | |
| 212230_at | 0.022 | 0.000 | -7.630 | 1.344 | -0.925 | PLPP3 | phospholipid phosphatase 3 | |
| 212226_s_at | 0.024 | 0.000 | -7.330 | 1.086 | -0.867 | PLPP3 | phospholipid phosphatase 3 | |
| 209355_s_at | 0.042 | 0.001 | -5.770 | -0.147 | -1.240 | PLPP3 | phospholipid phosphatase 3 | |
| 220686_s_at | 0.039 | 0.001 | 6.010 | -0.080 | 1.730 | PIWIL2 | piwi like RNA-mediated gene silencing 2 | |
| 202925_s_at | 0.022 | 0.000 | 7.560 | 1.284 | 0.988 | PLAGL2 | PLAG1 like zinc finger 2 | |
| 201860_s_at | 0.046 | 0.001 | 5.590 | -0.932 | 2.900 | PLAT | plasminogen activator, tissue type | |
| 211668_s_at | 0.016 | 0.000 | -8.610 | 1.835 | -0.602 | PLAU | plasminogen activator, urokinase | |
| 205479_s_at | 0.038 | 0.001 | -6.080 | -0.414 | -0.487 | PLAU | plasminogen activator, urokinase | |
| 211661_x_at | 0.024 | 0.000 | 7.440 | 1.050 | 0.925 | PTAFR | platelet activating factor receptor | |
| 206278_at | 0.025 | 0.000 | 7.220 | 1.009 | 0.899 | PTAFR | platelet activating factor receptor | |
| 218640_s_at | 0.015 | 0.000 | 9.420 | 2.604 | 1.220 | PLEKHF2 | pleckstrin homology and FYVE domain containing 2 | |
| 201410_at | 0.016 | 0.000 | -8.860 | 2.208 | -0.909 | PLEKHB2 | pleckstrin homology domain containing B2 | |
| 218223_s_at | 0.026 | 0.000 | 6.990 | 0.877 | 1.060 | PLEKHO1 | pleckstrin homology domain containing O1 | |
| 209803_s_at | 0.012 | 0.000 | -10.600 | 3.286 | -1.790 | PHLDA2 | pleckstrin homology like domain family A member 2 | |
| 206471_s_at | 0.021 | 0.000 | 7.750 | 1.451 | 1.040 | PLXNC1 | plexin C1 |  |
| 213241_at | 0.025 | 0.000 | 7.120 | 0.779 | 0.665 | PLXNC1 | plexin C1 |  |
| 206470_at | 0.043 | 0.001 | 5.720 | -0.298 | 0.786 | PLXNC1 | plexin C1 |  |
| 210036_s_at | 0.024 | 0.000 | 7.310 | 1.140 | 1.070 | KCNH2 | potassium voltage-gated channel subfamily H member 2 | |
| 217192_s_at | 0.042 | 0.001 | -5.780 | -0.313 | -0.725 | PRDM1 | PR/SET domain 1 | |
| 209848_s_at | 0.050 | 0.001 | 5.400 | -0.962 | 2.430 | PMEL | premelanosome protein | |
| 207782_s_at | 0.033 | 0.001 | 6.410 | 0.246 | 0.694 | PSEN1 | presenilin 1 |  |
| 203460_s_at | 0.037 | 0.001 | 6.140 | -0.064 | 0.865 | PSEN1 | presenilin 1 |  |
| 205628_at | 0.039 | 0.001 | -5.980 | -0.604 | -3.180 | PRIM2 | primase (DNA) subunit 2 | |
| 202619_s_at | 0.043 | 0.001 | -5.710 | -0.853 | -2.600 | PLOD2 | procollagen-lysine,2-oxoglutarate 5-dioxygenase 2 | |
| 200654_at | 0.022 | 0.000 | -7.560 | 1.298 | -0.990 | P4HB | prolyl 4-hydroxylase subunit beta | |
| 212216_at | 0.037 | 0.001 | -6.150 | -0.396 | -3.090 | PREPL | prolyl endopeptidase-like | |
| 204897_at | 0.016 | 0.000 | -8.800 | 2.102 | -0.957 | PTGER4 | prostaglandin E receptor 4 | |
| 205402_x_at | 0.044 | 0.001 | 5.680 | -0.793 | 2.480 | PRSS2 | protease, serine 2 | |
| 201114_x_at | 0.017 | 0.000 | -8.300 | 1.862 | -1.270 | PSMA7 | proteasome subunit alpha 7 | |
| 202545_at | 0.016 | 0.000 | 8.600 | 2.029 | 1.020 | PRKCD | protein kinase C delta | |
| 200604_s_at | 0.039 | 0.001 | 6.010 | -0.124 | 0.679 | PRKAR1A | protein kinase cAMP-dependent type I regulatory subunit alpha | |
| 212629_s_at | 0.025 | 0.000 | -7.220 | 1.017 | -1.020 | PKN2 | protein kinase N2 | |
| 200846_s_at | 0.024 | 0.000 | -7.440 | 1.244 | -1.100 | PPP1CA | protein phosphatase 1 catalytic subunit alpha | |
| 201407_s_at | 0.021 | 0.000 | -7.710 | 1.437 | -1.040 | PPP1CB | protein phosphatase 1 catalytic subunit beta | |
| 212680_x_at | 0.040 | 0.001 | 5.880 | -0.168 | 0.788 | PPP1R14B | protein phosphatase 1 regulatory inhibitor subunit 14B | |
| 37028_at | 0.039 | 0.001 | -6.010 | -0.414 | -0.472 | PPP1R15A | protein phosphatase 1 regulatory subunit 15A | |
| 200730_s_at | 0.012 | 0.000 | -10.100 | 2.985 | -1.430 | PTP4A1 | protein tyrosine phosphatase type IVA, member 1 | |
| 200733_s_at | 0.016 | 0.000 | -9.130 | 2.417 | -1.860 | PTP4A1 | protein tyrosine phosphatase type IVA, member 1 | |
| 216988_s_at | 0.018 | 0.000 | 8.190 | 1.668 | 0.789 | PTP4A2 | protein tyrosine phosphatase type IVA, member 2 | |
| 208616_s_at | 0.028 | 0.000 | 6.820 | 0.422 | 0.536 | PTP4A2 | protein tyrosine phosphatase type IVA, member 2 | |
| 221840_at | 0.050 | 0.001 | 5.410 | -0.902 | 0.582 | PTPRE | protein tyrosine phosphatase, receptor type E | |
| 206277_at | 0.043 | 0.001 | 5.720 | -0.190 | 1.100 | P2RY2 | purinergic receptor P2Y2 | |
| 221666_s_at | 0.031 | 0.000 | -6.540 | 0.427 | -0.860 | PYCARD | PYD and CARD domain containing | |
| 215342_s_at | 0.049 | 0.001 | -5.460 | -0.468 | -0.951 | RABGAP1L | RAB GTPase activating protein 1 like | |
| 219681_s_at | 0.026 | 0.000 | -6.980 | 0.743 | -0.719 | RAB11FIP1 | RAB11 family interacting protein 1 | |
| 209089_at | 0.018 | 0.000 | 8.100 | 1.595 | 0.763 | RAB5A | RAB5A, member RAS oncogene family | |
| 206113_s_at | 0.031 | 0.000 | 6.570 | 0.493 | 1.160 | RAB5A | RAB5A, member RAS oncogene family | |
| 200607_s_at | 0.041 | 0.001 | 5.850 | -0.199 | 0.800 | RAD21 | RAD21 cohesin complex component | |
| 204828_at | 0.029 | 0.000 | -6.710 | 0.649 | -1.230 | RAD9A | RAD9 checkpoint clamp component A | |
| 202640_s_at | 0.023 | 0.000 | -7.480 | 0.307 | -3.050 | RANBP3 | RAN binding protein 3 | |
| 212099_at | 0.016 | 0.000 | -8.520 | 1.991 | -0.984 | RHOB | ras homolog family member B | |
| 203175_at | 0.026 | 0.000 | 7.090 | 0.701 | 0.725 | RHOG | ras homolog family member G | |
| 212120_at | 0.018 | 0.000 | 8.160 | 1.722 | 0.950 | RHOQ | ras homolog family member Q | |
| 218088_s_at | 0.027 | 0.000 | -6.900 | 0.583 | -0.672 | RRAGC | Ras related GTP binding C | |
| 208640_at | 0.009 | 0.000 | 12.300 | 4.196 | 1.330 | RAC1 | ras-related C3 botulinum toxin substrate 1 (rho family, small GTP binding protein Rac1) | |
| 202963_at | 0.027 | 0.000 | 6.910 | 0.822 | 1.300 | RFX5 | regulatory factor X5 | |
| 201783_s_at | 0.028 | 0.000 | 6.780 | 0.599 | 0.904 | RELA | RELA proto-oncogene, NF-kB subunit | |
| 205205_at | 0.016 | 0.000 | 8.880 | 2.258 | 1.160 | RELB | RELB proto-oncogene, NF-kB subunit | |
| 58780_s_at | 0.031 | 0.000 | -6.530 | 0.434 | -0.852 | ARHGEF40 | Rho guanine nucleotide exchange factor 40 | |
| 220326_s_at | 0.041 | 0.001 | -5.850 | -0.148 | -1.020 | ARHGEF40 | Rho guanine nucleotide exchange factor 40 | |
| 201785_at | 0.034 | 0.001 | 6.320 | -0.399 | 2.720 | RNASE1 | ribonuclease A family member 1, pancreatic | |
| 213566_at | 0.016 | 0.000 | -9.050 | 2.318 | -1.640 | RNASE6 | ribonuclease A family member k6 | |
| 201890_at | 0.032 | 0.001 | -6.450 | -0.426 | -2.990 | RRM2 | ribonucleotide reductase regulatory subunit M2 | |
| 213038_at | 0.011 | 0.000 | 10.800 | 3.410 | 1.090 | RNF19B | ring finger protein 19B | |
| 36564_at | 0.016 | 0.000 | 9.030 | 2.243 | 0.935 | RNF19B | ring finger protein 19B | |
| 201846_s_at | 0.022 | 0.000 | -7.690 | 1.236 | -0.808 | RYBP | RING1 and YY1 binding protein | |
| 201844_s_at | 0.024 | 0.000 | -7.380 | 1.041 | -0.956 | RYBP | RING1 and YY1 binding protein | |
| 218997_at | 0.016 | 0.000 | -8.680 | 0.857 | -3.470 | POLR1E | RNA polymerase I subunit E | |
| 219957_at | 0.045 | 0.001 | -5.620 | -0.276 | -1.100 | RUFY2 | RUN and FYVE domain containing 2 | |
| 214370_at | 0.043 | 0.001 | -5.720 | -0.186 | -1.180 | S100A8 | S100 calcium binding protein A8 | |
| 204923_at | 0.018 | 0.000 | 8.120 | 1.707 | 1.130 | SASH3 | SAM and SH3 domain containing 3 | |
| 200961_at | 0.019 | 0.000 | -8.030 | 1.624 | -1.490 | SEPHS2 | selenophosphate synthetase 2 | |
| 205214_at | 0.016 | 0.000 | -8.520 | 2.003 | -1.020 | STK17B | serine/threonine kinase 17b | |
| 212268_at | 0.042 | 0.001 | 5.810 | -0.393 | 0.567 | SERPINB1 | serpin family B member 1 | |
| 216257_at | 0.042 | 0.001 | 5.770 | -0.685 | 2.800 | SERPINB13 | serpin family B member 13 | |
| 209722_s_at | 0.032 | 0.000 | 6.500 | 0.477 | 1.450 | SERPINB9 | serpin family B member 9 | |
| 221806_s_at | 0.037 | 0.001 | 6.160 | 0.044 | 0.737 | SETD5 | SET domain containing 5 | |
| 218793_s_at | 0.026 | 0.000 | -7.060 | -0.021 | -3.200 | SCML1 | sex comb on midleg-like 1 (Drosophila) | |
| 204657_s_at | 0.049 | 0.001 | 5.460 | -0.944 | 2.330 | SHB | SH2 domain containing adaptor protein B | |
| 203320_at | 0.019 | 0.000 | 8.030 | 1.637 | 0.912 | SH2B3 | SH2B adaptor protein 3 | |
| 209371_s_at | 0.044 | 0.001 | -5.660 | -0.298 | -0.990 | SH3BP2 | SH3 domain binding protein 2 | |
| 210101_x_at | 0.037 | 0.001 | 6.160 | -0.043 | 0.615 | SH3GLB1 | SH3 domain containing GRB2 like endophilin B1 | |
| 218765_at | 0.032 | 0.001 | -6.450 | 0.243 | -1.850 | SIDT2 | SID1 transmembrane family member 2 | |
| 213034_at | 0.038 | 0.001 | 6.100 | 0.095 | 1.040 | SIK3 | SIK family kinase 3 | |
| 218317_x_at | 0.039 | 0.001 | -6.020 | -0.303 | -2.140 | SLX1A///SLX1B | SLX1 homolog A, structure-specific endonuclease subunit///SLX1 homolog B, structure-specific endonuclease subunit | |
| 218284_at | 0.028 | 0.000 | 6.800 | 0.739 | 1.280 | SMAD3 | SMAD family member 3 | |
| 205443_at | 0.011 | 0.000 | -10.900 | 3.237 | -2.060 | SNAPC1 | small nuclear RNA activating complex polypeptide 1 | |
| 221891_x_at | 0.015 | 0.000 | -9.230 | 2.480 | -1.430 | SNORD14D///SNORD14C///HSPA8 | small nucleolar RNA, C/D box 14D///small nucleolar RNA, C/D box 14C///heat shock protein family A (Hsp70) member 8 | |
| 208687_x_at | 0.016 | 0.000 | -8.620 | 2.077 | -1.210 | SNORD14D///SNORD14C///HSPA8 | small nucleolar RNA, C/D box 14D///small nucleolar RNA, C/D box 14C///heat shock protein family A (Hsp70) member 8 | |
| 200725_x_at | 0.038 | 0.001 | -6.080 | -0.013 | -0.702 | SNORA70///RPL10 | small nucleolar RNA, H/ACA box 70///ribosomal protein L10 | |
| 206950_at | 0.039 | 0.001 | -6.000 | -0.750 | -2.810 | SCN9A | sodium voltage-gated channel alpha subunit 9 | |
| 203124_s_at | 0.025 | 0.000 | 7.150 | 0.997 | 1.180 | SLC11A2 | solute carrier family 11 member 2 | |
| 206600_s_at | 0.042 | 0.001 | -5.770 | -0.167 | -1.000 | SLC16A5 | solute carrier family 16 member 5 | |
| 209777_s_at | 0.024 | 0.000 | -7.370 | 0.898 | -2.090 | SLC19A1 | solute carrier family 19 member 1 | |
| 209681_at | 0.021 | 0.000 | -7.750 | 1.311 | -2.120 | SLC19A2 | solute carrier family 19 member 2 | |
| 216236_s_at | 0.049 | 0.001 | -5.460 | -0.687 | -0.595 | SLC2A14///SLC2A3 | solute carrier family 2 member 14///solute carrier family 2 member 3 | |
| 202497_x_at | 0.016 | 0.000 | -8.440 | 1.898 | -1.090 | SLC2A3 | solute carrier family 2 member 3 | |
| 201920_at | 0.042 | 0.001 | -5.790 | -0.515 | -0.676 | SLC20A1 | solute carrier family 20 member 1 | |
| 203775_at | 0.027 | 0.000 | 6.910 | 0.812 | 1.310 | SLC25A13 | solute carrier family 25 member 13 | |
| 218725_at | 0.039 | 0.001 | -6.020 | -0.589 | -2.550 | SLC25A22 | solute carrier family 25 member 22 | |
| 200924_s_at | 0.020 | 0.000 | -7.920 | 1.577 | -0.992 | SLC3A2 | solute carrier family 3 member 2 | |
| 207362_at | 0.039 | 0.001 | 6.040 | -0.546 | 2.700 | SLC30A4 | solute carrier family 30 member 4 | |
| 220814_at | 0.049 | 0.001 | -5.460 | -0.884 | -2.560 | SLC30A6 | solute carrier family 30 member 6 | |
| 204204_at | 0.040 | 0.001 | -5.890 | -0.139 | -0.811 | SLC31A2 | solute carrier family 31 member 2 | |
| 214712_at | 0.024 | 0.000 | -7.330 | -0.014 | -3.480 | SNX29P2 | sorting nexin 29 pseudogene 2 | |
| 206748_s_at | 0.020 | 0.000 | 7.840 | 1.541 | 1.260 | SPAG9 | sperm associated antigen 9 | |
| 212470_at | 0.033 | 0.001 | 6.390 | 0.145 | 0.651 | SPAG9 | sperm associated antigen 9 | |
| 203455_s_at | 0.028 | 0.000 | -6.860 | 0.438 | -0.557 | SAT1 | spermidine/spermine N1-acetyltransferase 1 | |
| 205312_at | 0.048 | 0.001 | 5.490 | -0.641 | 0.711 | SPI1 | Spi-1 proto-oncogene | |
| 203761_at | 0.016 | 0.000 | 8.550 | 1.963 | 0.965 | SLA | Src-like-adaptor | |
| 218033_s_at | 0.011 | 0.000 | 11.200 | 3.605 | 1.290 | SNN | stannin |  |
| 218032_at | 0.011 | 0.000 | 11.000 | 3.404 | 0.795 | SNN | stannin |  |
| 202308_at | 0.026 | 0.000 | 7.090 | 0.864 | 1.600 | SREBF1 | sterol regulatory element binding transcription factor 1 | |
| 206664_at | 0.028 | 0.000 | -6.830 | -0.386 | -3.170 | SI | sucrase-isomaltase | |
| 217995_at | 0.029 | 0.000 | 6.660 | 0.448 | 0.998 | SQRDL | sulfide quinone reductase-like (yeast) | |
| 211989_at | 0.016 | 0.000 | -8.660 | 0.760 | -3.250 | SMARCE1 | SWI/SNF related, matrix associated, actin dependent regulator of chromatin, subfamily e, member 1 | |
| 216180_s_at | 0.031 | 0.000 | -6.570 | -0.248 | -2.810 | SYNJ2 | synaptojanin 2 | |
| 219804_at | 0.048 | 0.001 | 5.500 | -0.972 | 2.680 | SYNPO2L | synaptopodin 2 like | |
| 210294_at | 0.046 | 0.001 | 5.560 | -0.414 | 1.190 | TAPBP | TAP binding protein (tapasin) | |
| 202807_s_at | 0.029 | 0.000 | 6.710 | 0.612 | 0.865 | TOM1 | target of myb1 membrane trafficking protein | |
| 212796_s_at | 0.023 | 0.000 | 7.520 | 1.301 | 1.200 | TBC1D2B | TBC1 domain family member 2B | |
| 212665_at | 0.008 | 0.000 | -12.700 | 4.322 | -1.510 | TIPARP | TCDD inducible poly(ADP-ribose) polymerase | |
| 205486_at | 0.023 | 0.000 | 7.470 | 1.254 | 1.180 | TESK2 | testis-specific kinase 2 | |
| 203313_s_at | 0.025 | 0.000 | -7.230 | 1.056 | -0.950 | TGIF1 | TGFB induced factor homeobox 1 | |
| 215009_s_at | 0.030 | 0.000 | -6.640 | 0.469 | -0.790 | THAP9-AS1 | THAP9 antisense RNA 1 | |
| 201266_at | 0.048 | 0.001 | 5.490 | -0.640 | 0.705 | TXNRD1 | thioredoxin reductase 1 | |
| 209605_at | 0.018 | 0.000 | -8.200 | 0.578 | -3.270 | TST | thiosulfate sulfurtransferase | |
| 210785_s_at | 0.023 | 0.000 | 7.510 | 1.146 | 0.740 | THEMIS2 | thymocyte selection associated family member 2 | |
| 207571_x_at | 0.041 | 0.001 | 5.860 | -0.375 | 0.601 | THEMIS2 | thymocyte selection associated family member 2 | |
| 203673_at | 0.044 | 0.001 | 5.670 | -0.285 | 1.520 | TG | thyroglobulin | |
| 201666_at | 0.039 | 0.001 | 6.050 | -0.112 | 0.696 | TIMP1 | TIMP metallopeptidase inhibitor 1 | |
| 202509_s_at | 0.016 | 0.000 | 8.570 | 2.050 | 1.640 | TNFAIP2 | TNF alpha induced protein 2 | |
| 202510_s_at | 0.034 | 0.001 | 6.370 | -0.080 | 0.625 | TNFAIP2 | TNF alpha induced protein 2 | |
| 206025_s_at | 0.016 | 0.000 | 8.730 | 1.840 | 0.731 | TNFAIP6 | TNF alpha induced protein 6 | |
| 210260_s_at | 0.021 | 0.000 | 7.740 | 1.436 | 1.000 | TNFAIP8 | TNF alpha induced protein 8 | |
| 205599_at | 0.016 | 0.000 | 8.410 | 1.880 | 1.520 | TRAF1 | TNF receptor associated factor 1 | |
| 208315_x_at | 0.050 | 0.001 | 5.410 | -0.512 | 0.930 | TRAF3 | TNF receptor associated factor 3 | |
| 209294_x_at | 0.034 | 0.001 | 6.360 | 0.320 | 1.790 | TNFRSF10B | TNF receptor superfamily member 10b | |
| 207196_s_at | 0.008 | 0.000 | 13.300 | 4.706 | 1.360 | TNIP1 | TNFAIP3 interacting protein 1 | |
| 204924_at | 0.024 | 0.000 | 7.400 | 0.922 | 0.602 | TLR2 | toll like receptor 2 | |
| 221060_s_at | 0.018 | 0.000 | 8.090 | 1.686 | 1.140 | TLR4 | toll like receptor 4 | |
| 207616_s_at | 0.014 | 0.000 | 9.750 | 2.731 | 1.290 | TANK | TRAF family member associated NFKB activator | |
| 210458_s_at | 0.041 | 0.001 | 5.840 | -0.123 | 0.889 | TANK | TRAF family member associated NFKB activator | |
| 202371_at | 0.015 | 0.000 | -9.330 | 1.268 | -3.880 | TCEAL4 | transcription elongation factor A like 4 | |
| 217566_s_at | 0.047 | 0.001 | -5.530 | -0.912 | -2.620 | TGM4 | transglutaminase 4 | |
| 201078_at | 0.039 | 0.001 | -5.990 | -0.132 | -0.725 | TM9SF2 | transmembrane 9 superfamily member 2 | |
| 214550_s_at | 0.039 | 0.001 | -6.030 | -0.523 | -2.700 | TNPO3 | transportin 3 | |
| 202241_at | 0.021 | 0.000 | -7.730 | 1.192 | -0.588 | TRIB1 | tribbles pseudokinase 1 | |
| 217839_at | 0.032 | 0.000 | 6.490 | 0.359 | 0.784 | TFG | TRK-fused gene | |
| 217774_s_at | 0.041 | 0.001 | -5.870 | -0.256 | -0.634 | TRMT112 | tRNA methyltransferase 11-2 homolog (S. cerevisiae) | |
| 221952_x_at | 0.034 | 0.001 | -6.350 | 0.165 | -0.696 | TRMT5 | tRNA methyltransferase 5 | |
| 209118_s_at | 0.019 | 0.000 | -8.050 | 1.452 | -0.810 | TUBA1A | tubulin alpha 1a | |
| 212639_x_at | 0.016 | 0.000 | -8.940 | 2.145 | -0.798 | TUBA1B | tubulin alpha 1b | |
| 211072_x_at | 0.034 | 0.001 | -6.320 | 0.039 | -0.660 | TUBA1B | tubulin alpha 1b | |
| 209251_x_at | 0.016 | 0.000 | -8.480 | 1.855 | -0.768 | TUBA1C | tubulin alpha 1c | |
| 211750_x_at | 0.025 | 0.000 | -7.160 | 0.770 | -0.865 | TUBA1C | tubulin alpha 1c | |
| 212242_at | 0.039 | 0.001 | -6.020 | -0.088 | -0.763 | TUBA4A | tubulin alpha 4a | |
| 203690_at | 0.039 | 0.001 | -6.010 | -0.692 | -2.750 | TUBGCP3 | tubulin gamma complex associated protein 3 | |
| 203702_s_at | 0.019 | 0.000 | 7.980 | 1.608 | 1.600 | TTLL4 | tubulin tyrosine ligase like 4 | |
| 207113_s_at | 0.022 | 0.000 | 7.590 | 1.310 | 1.020 | TNF | tumor necrosis factor | |
| 202687_s_at | 0.029 | 0.000 | -6.670 | 0.622 | -1.020 | TNFSF10 | tumor necrosis factor superfamily member 10 | |
| 210996_s_at | 0.040 | 0.001 | 5.910 | -0.096 | 0.929 | YWHAE | tyrosine 3-monooxygenase/tryptophan 5-monooxygenase activation protein epsilon | |
| 203838_s_at | 0.049 | 0.001 | 5.430 | -0.949 | 2.380 | TNK2 | tyrosine kinase non receptor 2 | |
| 209066_x_at | 0.046 | 0.001 | -5.610 | -0.415 | -0.751 | UQCRB | ubiquinol-cytochrome c reductase binding protein | |
| 218190_s_at | 0.030 | 0.000 | -6.600 | 0.462 | -0.855 | UQCR10 | ubiquinol-cytochrome c reductase, complex III subunit X | |
| 202090_s_at | 0.022 | 0.000 | -7.560 | 1.259 | -0.960 | UQCR11 | ubiquinol-cytochrome c reductase, complex III subunit XI | |
| 208909_at | 0.012 | 0.000 | -10.300 | 3.152 | -1.420 | UQCRFS1 | ubiquinol-cytochrome c reductase, Rieske iron-sulfur polypeptide 1 | |
| 201377_at | 0.016 | 0.000 | -8.460 | 1.975 | -1.400 | UBAP2L | ubiquitin associated protein 2 like | |
| 221962_s_at | 0.030 | 0.000 | 6.630 | 0.554 | 0.900 | UBE2H | ubiquitin conjugating enzyme E2 H | |
| 211950_at | 0.040 | 0.001 | 5.890 | -0.187 | 0.687 | UBR4 | ubiquitin protein ligase E3 component n-recognin 4 | |
| 203965_at | 0.048 | 0.001 | -5.500 | -0.874 | -2.810 | USP20 | ubiquitin specific peptidase 20 | |
| 215078_at | 0.008 | 0.000 | -12.900 | 4.494 | -1.090 | LOC100129518///SOD2 | uncharacterized LOC100129518///superoxide dismutase 2, mitochondrial | |
| 206374_at | 0.034 | 0.001 | -6.340 | -0.318 | -2.770 | LOC101927562///DUSP8 | uncharacterized LOC101927562///dual specificity phosphatase 8 | |
| 221213_s_at | 0.047 | 0.001 | -5.550 | -0.983 | -2.690 | LOC145783///ZNF280D | uncharacterized LOC145783///zinc finger protein 280D | |
| 205931_s_at | 0.022 | 0.000 | -7.610 | 1.371 | -1.560 | LOC401317///CREB5 | uncharacterized LOC401317///cAMP responsive element binding protein 5 | |
| 206922_at | 0.050 | 0.001 | -5.400 | -0.912 | -2.610 | VCY1B///VCY | variable charge, Y-linked 1B///variable charge, Y-linked | |
| 210512_s_at | 0.004 | 0.000 | -16.700 | 6.019 | -1.550 | VEGFA | vascular endothelial growth factor A | |
| 210513_s_at | 0.008 | 0.000 | -12.900 | 4.457 | -1.470 | VEGFA | vascular endothelial growth factor A | |
| 212171_x_at | 0.008 | 0.000 | -12.700 | 4.385 | -1.190 | VEGFA | vascular endothelial growth factor A | |
| 211527_x_at | 0.011 | 0.000 | -11.400 | 3.756 | -1.850 | VEGFA | vascular endothelial growth factor A | |
| 201557_at | 0.043 | 0.001 | -5.720 | -0.297 | -0.785 | VAMP2 | vesicle associated membrane protein 2 | |
| 208091_s_at | 0.027 | 0.000 | 6.890 | 0.681 | 0.854 | VOPP1 | vesicular, overexpressed in cancer, prosurvival protein 1 | |
| 205586_x_at | 0.047 | 0.001 | -5.530 | -0.596 | -1.910 | VGF | VGF nerve growth factor inducible | |
| 209950_s_at | 0.026 | 0.000 | 7.060 | -0.038 | 3.160 | VILL | villin like |  |
| 200611_s_at | 0.040 | 0.001 | 5.900 | -0.321 | 0.652 | WDR1 | WD repeat domain 1 | |
| 211992_at | 0.042 | 0.001 | -5.770 | -0.265 | -0.828 | WNK1 | WNK lysine deficient protein kinase 1 | |
| 217681_at | 0.039 | 0.001 | 5.970 | -0.650 | 2.650 | WNT7B | Wnt family member 7B | |
| 221203_s_at | 0.028 | 0.000 | -6.810 | -0.178 | -2.960 | YEATS2 | YEATS domain containing 2 | |
| 201531_at | 0.025 | 0.000 | -7.230 | 0.632 | -0.464 | ZFP36 | ZFP36 ring finger protein | |
| 201369_s_at | 0.011 | 0.000 | 10.800 | 3.372 | 1.360 | ZFP36L2 | ZFP36 ring finger protein like 2 | |
| 201367_s_at | 0.013 | 0.000 | 9.880 | 2.884 | 1.370 | ZFP36L2 | ZFP36 ring finger protein like 2 | |
| 201368_at | 0.015 | 0.000 | 9.460 | 2.524 | 0.925 | ZFP36L2 | ZFP36 ring finger protein like 2 | |
| 222016_s_at | 0.041 | 0.001 | -5.850 | -0.677 | -2.640 | ZSCAN31 | zinc finger and SCAN domain containing 31 | |
| 217594_at | 0.033 | 0.001 | -6.390 | -0.318 | -2.760 | ZCCHC11 | zinc finger CCHC-type containing 11 | |
| 212860_at | 0.031 | 0.000 | 6.550 | 0.312 | 0.661 | ZDHHC18 | zinc finger DHHC-type containing 18 | |
| 220836_at | 0.026 | 0.000 | -7.030 | -0.297 | -3.190 | ZNF407 | zinc finger protein 407 | |
| 211064_at | 0.028 | 0.000 | -6.850 | -0.116 | -3.000 | ZNF493 | zinc finger protein 493 | |
| 207120_at | 0.035 | 0.001 | 6.270 | -0.609 | 3.270 | ZNF667 | zinc finger protein 667 | |
| 214138_at | 0.034 | 0.001 | 6.360 | -0.366 | 2.670 | ZNF79 | zinc finger protein 79 | |

Table S2: 886 annotated candidate gastric cancer human genes identified in the CGGD database

| **HumanName** | **HumanId** | **homologId** | **Study** | **PubMedId** | **Effect** | **CISAddress** | **Rank** | **Studies** |
| --- | --- | --- | --- | --- | --- | --- | --- | --- |
| AAK1 | 22848 | 128746 | Takeda 2016-01 | 27006499 | Loss | 6:86851258-86880586 | B | 8 |
| ABCD3 | 5825 | 2140 | Takeda 2016-01 | 27006499 | Gain | 3:121799530-121817178 | D | 5 |
| ABHD2 | 11057 | 23121 | Takeda 2016-01 | 27006499 | Loss | 7:79311971-79329558 | D | 8 |
| ABI1 | 10006 | 38053 | Takeda 2016-01 | 27006499 | Loss | 2:22945929-22984289 | B | 15 |
| ABL1 | 25 | 3783 | Takeda 2016-01 | 27006499 | Loss | 2:31719889-31746446 | C | 10 |
| ABL2 | 27 | 5278 | Takeda 2016-01 | 27006499 | Loss | 1:156564964-156570871 | C | 5 |
| ACBD3 | 64746 | 11227 | Takeda 2016-01 | 27006499 | Loss | 1:180734269-180771261 | C | 4 |
| ACIN1 | 22985 | 22853 | Takeda 2016-01 | 27006499 | Loss | 14:54646540-54675335 | D | 7 |
| ACP1 | 52 | 38274 | Takeda 2016-01 | 27006499 | N/A | 12:30893326-30911612 | D | 1 |
| ACRBP | 84519 | 9641 | Takeda 2016-01 | 27006499 | N/A | 6:125019915-125098237 | B | 3 |
| ACTR3 | 10096 | 68483 | Takeda 2016-01 | 27006499 | Loss | 1:125402574-125420296 | D | 7 |
| ACVR1 | 90 | 7 | Takeda 2016-01 | 27006499 | Loss | 2:58497813-58522894 | C | 4 |
| ACVR2A | 92 | 20391 | Takeda 2016-01 | 27006499 | N/A | 2:48814109-48903269 | C | 13 |
| ADAM17 | 6868 | 2395 | Takeda 2016-01 | 27006499 | Loss | 12:21329119-21349578 | C | 7 |
| ADAM9 | 8754 | 20824 | Takeda 2016-01 | 27006499 | Loss | 8:24979279-25008577 | C | 1 |
| ADD1 | 118 | 22758 | Takeda 2016-01 | 27006499 | Loss | 5:34584195-34607657 | D | 6 |
| ADIPOR2 | 79602 | 56119 | Takeda 2016-01 | 27006499 | Loss | 6:119354620-119361952 | D | 9 |
| ADNP | 23394 | 7617 | Takeda 2016-01 | 27006499 | Loss | 2:168183731-168195534 | D | 14 |
| AEBP2 | 121536 | 40690 | Takeda 2016-01 | 27006499 | N/A | 6:140622663-140678472 | D | 8 |
| AFF1 | 4299 | 4340 | Takeda 2016-01 | 27006499 | Loss | 5:103821077-103847472 | B | 15 |
| AFF4 | 27125 | 8683 | Takeda 2016-01 | 27006499 | N/A | 11:53350833-53421830 | D | 12 |
| AFTPH | 54812 | 9764 | Takeda 2016-01 | 27006499 | Loss | 11:20690532-20728560 | C | 8 |
| AGFG1 | 3267 | 37929 | Takeda 2016-01 | 27006499 | Loss | 1:82860576-82885683 | C | 3 |
| AGO2 | 27161 | 81825 | Takeda 2016-01 | 27006499 | Loss | 15:73112855-73182824 | B | 11 |
| AGPS | 8540 | 2716 | Takeda 2016-01 | 27006499 | Loss | 2:75900748-75919928 | C | 5 |
| AK3 | 50808 | 21744 | Takeda 2016-01 | 27006499 | N/A | 19:29020833-29047961 | D | 5 |
| AKAP13 | 11214 | 4903 | Takeda 2016-01 | 27006499 | Loss | 7:75508048-75519766 | D | 14 |
| AKAP8 | 10270 | 4278 | Takeda 2016-01 | 27006499 | N/A | 17:32303676-32321153 | D | 6 |
| AKAP8L | 26993 | 8658 | Takeda 2016-01 | 27006499 | Loss | 17:32315321-32338537 | D | 5 |
| AMBRA1 | 55626 | 18204 | Takeda 2016-01 | 27006499 | Loss | 2:91826623-91859081 | A | 19 |
| AMFR | 267 | 888 | Takeda 2016-01 | 27006499 | N/A | 8:93971588-94012663 | D | 7 |
| AMOT | 154796 | 15778 | Takeda 2016-01 | 27006499 | Loss | X:145447189-145464585 | D | 1 |
| ANKFY1 | 51479 | 9491 | Takeda 2016-01 | 27006499 | Loss | 11:72681734-72732925 | B | 8 |
| ANKHD1 | 54882 | 87006 | Takeda 2016-01 | 27006499 | Loss | 18:36579452-36631711 | B | 13 |
| ANKRD11 | 29123 | 69134 | Takeda 2016-01 | 27006499 | Loss | 8:122927105-122997420 | B | 21 |
| ANKRD17 | 26057 | 82403 | Takeda 2016-01 | 27006499 | Loss | 5:90274626-90362609 | A | 17 |
| ANKRD28 | 23243 | 35374 | Takeda 2016-01 | 27006499 | Loss | 14:31797350-31824706 | D | 10 |
| ANKRD40 | 91369 | 12393 | Takeda 2016-01 | 27006499 | N/A | 11:94328001-94341841 | D | 6 |
| AP3B1 | 8546 | 68125 | Takeda 2016-01 | 27006499 | Loss | 13:94370320-94390756 | D | 6 |
| APC | 324 | 30950 | Takeda 2016-01 | 27006499 | Loss | 18:34290212-34316342 | B | 12 |
| APP | 351 | 56379 | Takeda 2016-01 | 27006499 | Loss | 16:84992669-85027329 | C | 17 |
| ARF4 | 378 | 55593 | Takeda 2016-01 | 27006499 | Loss | 14:26625141-26655377 | C | 8 |
| ARFGEF1 | 10565 | 4687 | Takeda 2016-01 | 27006499 | Loss | 1:10196160-10227174 | B | 5 |
| ARFIP1 | 27236 | 8692 | Takeda 2016-01 | 27006499 | Loss | 3:84821802-84982110 | A | 15 |
| ARFIP2 | 23647 | 8234 | Takeda 2016-01 | 27006499 | N/A | 7:105634203-105640416 | D | 1 |
| ARGLU1 | 55082 | 9960 | Takeda 2016-01 | 27006499 | Loss | 8:8660368-8677946 | D | 10 |
| ARHGAP17 | 55114 | 9984 | Takeda 2016-01 | 27006499 | Loss | 7:123335389-123366148 | C | 3 |
| ARHGAP21 | 57584 | 10822 | Takeda 2016-01 | 27006499 | Loss | 2:20852679-20860056 | B | 14 |
| ARHGAP5 | 394 | 907 | Takeda 2016-01 | 27006499 | Loss | 12:52505055-52566383 | B | 16 |
| ARHGAP6 | 395 | 7630 | Takeda 2016-01 | 27006499 | Loss | X:169191287-169247825 | C | 5 |
| ARHGDIA | 396 | 908 | Takeda 2016-01 | 27006499 | N/A | 11:120578104-120581624 | D | 4 |
| ARID1A | 8289 | 21216 | Takeda 2016-01 | 27006499 | Loss | 4:133677869-133752666 | A | 14 |
| ARID1B | 57492 | 32344 | Takeda 2016-01 | 27006499 | Loss | 17:5004129-5126018 | A | 19 |
| ARID2 | 196528 | 14601 | Takeda 2016-01 | 27006499 | Loss | 15:96302708-96363876 | B | 13 |
| ARID4B | 51742 | 12847 | Takeda 2016-01 | 27006499 | Loss | 13:14075080-14158282 | B | 12 |
| ARL15 | 54622 | 56843 | Takeda 2016-01 | 27006499 | Loss | 13:114003223-114025118 | A | 2 |
| ARL8B | 55207 | 10056 | Takeda 2016-01 | 27006499 | Loss | 6:108806624-108818356 | D | 7 |
| ASAP1 | 50807 | 7684 | Takeda 2016-01 | 27006499 | Loss | 15:64213119-64233508 | D | 8 |
| ASCC3 | 10973 | 4973 | Takeda 2016-01 | 27006499 | Loss | 10:50747648-50757422 | B | 3 |
| ASH1L | 55870 | 10225 | Takeda 2016-01 | 27006499 | Loss | 3:88938335-89072170 | A | 18 |
| ASXL2 | 55252 | 10102 | Takeda 2016-01 | 27006499 | Loss | 12:3452307-3485892 | C | 17 |
| ATAD2B | 54454 | 86351 | Takeda 2016-01 | 27006499 | Loss | 12:4920310-4946594 | B | 16 |
| ATE1 | 11101 | 31420 | Takeda 2016-01 | 27006499 | Loss | 7:130413559-130439940 | C | 2 |
| ATF7 | 11016 | 4994 | Takeda 2016-01 | 27006499 | N/A | 15:102525946-102625464 | D | 10 |
| ATF7IP | 55729 | 10051 | Takeda 2016-01 | 27006499 | Loss | 6:136535326-136561722 | C | 13 |
| ATG13 | 9776 | 32229 | Takeda 2016-01 | 27006499 | Loss | 2:91674661-91704169 | D | 2 |
| ATG3 | 64422 | 6836 | Takeda 2016-01 | 27006499 | N/A | 16:45158785-45188538 | D | 4 |
| ATP11B | 23200 | 32919 | Takeda 2016-01 | 27006499 | Loss | 3:35771091-35787269 | C | 5 |
| ATP2A2 | 488 | 80167 | Takeda 2016-01 | 27006499 | Loss | 5:122471541-122499402 | C | 12 |
| ATP2B1 | 490 | 55597 | Takeda 2016-01 | 27006499 | Loss | 10:98950794-98962503 | C | 3 |
| ATP5J | 522 | 1272 | Takeda 2016-01 | 27006499 | Loss | 16:84821830-84842029 | D | 4 |
| ATP8B1 | 5205 | 21151 | Takeda 2016-01 | 27006499 | Loss | 18:64591790-64616468 | C | 12 |
| ATP9B | 374868 | 21915 | Takeda 2016-01 | 27006499 | Loss | 18:80803618-80861926 | B | 7 |
| ATRX | 546 | 416 | Takeda 2016-01 | 27006499 | Loss | X:105885796-105994766 | C | 12 |
| ATXN1 | 6310 | 281 | Takeda 2016-01 | 27006499 | Loss | 13:45885743-45895961 | D | 7 |
| ATXN2 | 6311 | 2234 | Takeda 2016-01 | 27006499 | Loss | 5:121735417-121794072 | B | 15 |
| ATXN3 | 4287 | 3658 | Takeda 2016-01 | 27006499 | Loss | 12:101917405-101951537 | C | 2 |
| AXIN1 | 8312 | 2614 | Takeda 2016-01 | 27006499 | Loss | 17:26149792-26164303 | D | 14 |
| AZIN1 | 51582 | 22933 | Takeda 2016-01 | 27006499 | Loss | 15:38492802-38510279 | D | 8 |
| B3GALT2 | 8707 | 74512 | Takeda 2016-01 | 27006499 | N/A | 1:143640664-143654614 | D | 2 |
| BACH1 | 571 | 916 | Takeda 2016-01 | 27006499 | Loss | 16:87701625-87731923 | D | 5 |
| BAIAP2 | 10458 | 9697 | Takeda 2016-01 | 27006499 | N/A | 11:119942763-120006782 | D | 7 |
| BAZ1B | 9031 | 22651 | Takeda 2016-01 | 27006499 | Loss | 5:135206144-135225207 | D | 15 |
| BAZ2B | 29994 | 8394 | Takeda 2016-01 | 27006499 | Loss | 2:60043123-60072665 | B | 4 |
| BBX | 56987 | 10634 | Takeda 2016-01 | 27006499 | Loss | 16:50275965-50329348 | A | 2 |
| BCL11A | 53335 | 11284 | Takeda 2016-01 | 27006499 | Loss | 11:24153969-24181758 | C | 2 |
| BCOR | 54880 | 9809 | Takeda 2016-01 | 27006499 | Loss | X:12115500-12139694 | D | 7 |
| BCORL1 | 63035 | 41458 | Takeda 2016-01 | 27006499 | Loss | X:48369492-48413040 | C | 3 |
| BMPR1A | 657 | 20911 | Takeda 2016-01 | 27006499 | Loss | 14:34406220-34440775 | C | 15 |
| BMPR2 | 659 | 929 | Takeda 2016-01 | 27006499 | Loss | 1:59827091-59859597 | C | 4 |
| BNIP1 | 662 | 930 | Takeda 2016-01 | 27006499 | N/A | 17:26781079-26792565 | D | 2 |
| BRE | 9577 | 3604 | Takeda 2016-01 | 27006499 | Loss | 5:31812920-32001415 | A | 4 |
| BRWD3 | 254065 | 18736 | Takeda 2016-01 | 27006499 | Loss | X:108731007-108751303 | D | 2 |
| BTAF1 | 9044 | 31978 | Takeda 2016-01 | 27006499 | Loss | 19:36929086-36939038 | C | 8 |
| BTBD1 | 53339 | 23529 | Takeda 2016-01 | 27006499 | Loss | 7:81752140-81825376 | B | 5 |
| C1GALT1 | 56913 | 10599 | Takeda 2016-01 | 27006499 | N/A | 6:7844842-7875687 | D | 5 |
| CABLES2 | 81928 | 45440 | Takeda 2016-01 | 27006499 | N/A | 2:180258540-180273496 | D | 3 |
| CAMK2D | 817 | 55561 | Takeda 2016-01 | 27006499 | Loss | 3:126768200-126777029 | C | 9 |
| CANX | 821 | 1324 | Takeda 2016-01 | 27006499 | Loss | 11:50297785-50346626 | B | 9 |
| CAPN1 | 823 | 3800 | Takeda 2016-01 | 27006499 | N/A | 19:5988546-6015825 | D | 2 |
| CAPZB | 832 | 3620 | Takeda 2016-01 | 27006499 | Loss | 4:139197845-139253610 | D | 10 |
| CASK | 8573 | 2736 | Takeda 2016-01 | 27006499 | Loss | X:13729556-13746962 | C | 9 |
| CBFB | 865 | 11173 | Takeda 2016-01 | 27006499 | Loss | 8:105177024-105241526 | C | 10 |
| CBLB | 868 | 15856 | Takeda 2016-01 | 27006499 | Loss | 16:52080887-52096757 | B | 10 |
| CCDC34 | 91057 | 12245 | Takeda 2016-01 | 27006499 | N/A | 2:110017817-110173360 | D | 3 |
| CCNT2 | 905 | 14043 | Takeda 2016-01 | 27006499 | Loss | 1:127771475-127799535 | D | 5 |
| CCNY | 219771 | 12219 | Takeda 2016-01 | 27006499 | Loss | 18:9380009-9398880 | C | 13 |
| CCSER2 | 54462 | 10367 | Takeda 2016-01 | 27006499 | Loss | 14:36878312-36898468 | C | 8 |
| CD2AP | 23607 | 7663 | Takeda 2016-01 | 27006499 | Loss | 17:42845615-42857223 | C | 16 |
| CD47 | 961 | 1346 | Takeda 2016-01 | 27006499 | Loss | 16:49870543-49886414 | D | 6 |
| CDC14A | 8556 | 75343 | Takeda 2016-01 | 27006499 | Loss | 3:116400533-116418181 | C | 6 |
| CDC37L1 | 55664 | 9912 | Takeda 2016-01 | 27006499 | Loss | 19:29005786-29022847 | D | 5 |
| CDC40 | 51362 | 5716 | Takeda 2016-01 | 27006499 | Loss | 10:40840097-40873760 | D | 3 |
| CDC73 | 79577 | 11571 | Takeda 2016-01 | 27006499 | Loss | 1:143617533-143651501 | B | 2 |
| CDK12 | 51755 | 128632 | Takeda 2016-01 | 27006499 | Loss | 11:98201609-98242562 | C | 12 |
| CDK13 | 8621 | 135707 | Takeda 2016-01 | 27006499 | Loss | 13:17689685-17723258 | C | 19 |
| CDK17 | 5128 | 55666 | Takeda 2016-01 | 27006499 | Loss | 10:93170367-93190872 | C | 8 |
| CDK8 | 1024 | 55565 | Takeda 2016-01 | 27006499 | Loss | 5:146238529-146291352 | C | 7 |
| CDYL | 9425 | 3548 | Takeda 2016-01 | 27006499 | Loss | 13:35786785-35801382 | D | 3 |
| CELF2 | 10659 | 4783 | Takeda 2016-01 | 27006499 | Loss | 2:6675060-6700141 | C | 9 |
| CEP350 | 9857 | 8879 | Takeda 2016-01 | 27006499 | Loss | 1:155873657-155909219 | A | 4 |
| CEP85L | 387119 | 52598 | Takeda 2016-01 | 27006499 | Loss | 10:53302726-53334925 | C | 8 |
| CEP95 | 90799 | 16297 | Takeda 2016-01 | 27006499 | N/A | 11:106789275-106819930 | D | 5 |
| CERS6 | 253782 | 72228 | Takeda 2016-01 | 27006499 | Loss | 2:68953282-68985740 | B | 11 |
| CGGBP1 | 8545 | 2718 | Takeda 2016-01 | 27006499 | Gain | 16:64777664-64841147 | A | 7 |
| CHCHD3 | 54927 | 9851 | Takeda 2016-01 | 27006499 | Loss | 6:32971738-33031861 | A | 9 |
| CHD1 | 1105 | 68174 | Takeda 2016-01 | 27006499 | N/A | 17:15704967-15772610 | D | 13 |
| CHD2 | 1106 | 37462 | Takeda 2016-01 | 27006499 | Loss | 7:73464157-73516919 | C | 15 |
| CHD6 | 84181 | 32772 | Takeda 2016-01 | 27006499 | Loss | 2:161072265-161079641 | D | 10 |
| CHD9 | 80205 | 11844 | Takeda 2016-01 | 27006499 | Loss | 8:90995210-91005464 | D | 12 |
| CHIC2 | 26511 | 8105 | Takeda 2016-01 | 27006499 | Loss | 5:75029382-75038181 | D | 8 |
| CHN2 | 1124 | 31213 | Takeda 2016-01 | 27006499 | Loss | 6:54145504-54160168 | D | 4 |
| CLCN3 | 1182 | 20435 | Takeda 2016-01 | 27006499 | Loss | 8:60920498-60952726 | C | 4 |
| CLINT1 | 9685 | 133740 | Takeda 2016-01 | 27006499 | Loss | 11:45845974-45895702 | C | 12 |
| CLPTM1L | 81037 | 12767 | Takeda 2016-01 | 27006499 | Loss | 13:73603892-73621408 | D | 2 |
| CLTC | 1213 | 3572 | Takeda 2016-01 | 27006499 | Loss | 11:86686116-86716831 | C | 18 |
| CMIP | 80790 | 18869 | Takeda 2016-01 | 27006499 | Loss | 8:117378062-117411755 | C | 11 |
| CMSS1 | 84319 | 11979 | Takeda 2016-01 | 27006499 | Gain | 16:57554805-57575004 | C | 5 |
| CNBP | 7555 | 2567 | Takeda 2016-01 | 27006499 | Loss | 6:87841097-87846963 | D | 7 |
| CNOT1 | 23019 | 9453 | Takeda 2016-01 | 27006499 | Loss | 8:95737091-95810336 | B | 18 |
| CNOT2 | 4848 | 40953 | Takeda 2016-01 | 27006499 | Loss | 10:116476741-116585048 | A | 18 |
| CNOT3 | 4849 | 133900 | Takeda 2016-01 | 27006499 | N/A | 7:3645268-3661109 | D | 2 |
| CNOT4 | 4850 | 40870 | Takeda 2016-01 | 27006499 | Loss | 6:35105391-35125921 | B | 7 |
| CNOT6 | 57472 | 69187 | Takeda 2016-01 | 27006499 | Loss | 11:49673595-49707235 | C | 10 |
| CNOT6L | 246175 | 100830 | Takeda 2016-01 | 27006499 | Loss | 5:96119634-96140164 | C | 15 |
| CNTF | 1270 | 8288 | Takeda 2016-01 | 27006499 | N/A | 19:12763660-12765632 | D | 6 |
| CNTNAP2 | 26047 | 69159 | Takeda 2016-01 | 27006499 | N/A | 6:45059357-47304213 | A | 5 |
| COBLL1 | 22837 | 8933 | Takeda 2016-01 | 27006499 | Loss | 2:65201477-65225095 | B | 5 |
| COG3 | 83548 | 5854 | Takeda 2016-01 | 27006499 | Loss | 14:75704856-75712055 | C | 5 |
| COG5 | 10466 | 42221 | Takeda 2016-01 | 27006499 | Loss | 12:31923398-31933620 | A | 5 |
| COL4A3BP | 10087 | 4173 | Takeda 2016-01 | 27006499 | Loss | 13:96607323-96659913 | B | 6 |
| COMMD1 | 150684 | 17604 | Takeda 2016-01 | 27006499 | N/A | 11:22896136-22982382 | D | 3 |
| COMT | 1312 | 30982 | Takeda 2016-01 | 27006499 | Gain | 16:18406351-18419336 | D | 5 |
| COPA | 1314 | 3218 | Takeda 2016-01 | 27006499 | Loss | 1:172089831-172117892 | D | 2 |
| COPB1 | 1315 | 5664 | Takeda 2016-01 | 27006499 | Loss | 7:114214590-114238026 | D | 10 |
| COX16 | 51241 | 9520 | Takeda 2016-01 | 27006499 | Loss | 12:81475564-81500388 | D | 3 |
| CPEB2 | 132864 | 17995 | Takeda 2016-01 | 27006499 | Loss | 5:43257650-43272314 | D | 5 |
| CPNE1 | 8904 | 36501 | Takeda 2016-01 | 27006499 | Loss | 2:156082601-156113583 | D | 9 |
| CPSF7 | 79869 | 11731 | Takeda 2016-01 | 27006499 | N/A | 19:10525244-10547735 | D | 10 |
| CREB1 | 1385 | 3223 | Takeda 2016-01 | 27006499 | Loss | 1:64519531-64578606 | B | 5 |
| CREB3L2 | 64764 | 18690 | Takeda 2016-01 | 27006499 | Loss | 6:37366060-37401275 | C | 6 |
| CREBBP | 1387 | 68393 | Takeda 2016-01 | 27006499 | Loss | 16:4151748-4189260 | C | 32 |
| CRIM1 | 51232 | 9510 | Takeda 2016-01 | 27006499 | Loss | 17:78271896-78276249 | D | 2 |
| CRK | 1398 | 81850 | Takeda 2016-01 | 27006499 | Loss | 11:75690152-75729224 | B | 9 |
| CSDE1 | 7812 | 5179 | Takeda 2016-01 | 27006499 | Loss | 3:103032202-103042497 | D | 14 |
| CSE1L | 1434 | 1006 | Takeda 2016-01 | 27006499 | Loss | 2:166924190-166944855 | D | 5 |
| CSMD1 | 64478 | 69536 | Takeda 2016-01 | 27006499 | N/A | 8:15892537-17535586 | C | 7 |
| CSMD3 | 114788 | 65982 | Takeda 2016-01 | 27006499 | N/A | 15:47580637-48792063 | D | 6 |
| CSNK1A1 | 1452 | 111694 | Takeda 2016-01 | 27006499 | Loss | 18:61566568-61597052 | C | 17 |
| CSNK1D | 1453 | 74841 | Takeda 2016-01 | 27006499 | Loss | 11:120955257-120988896 | C | 12 |
| CSNK1G3 | 1456 | 121650 | Takeda 2016-01 | 27006499 | Loss | 18:53877283-53917971 | B | 13 |
| CSTF3 | 1479 | 1014 | Takeda 2016-01 | 27006499 | N/A | 2:104590523-104665429 | D | 12 |
| CTBP2 | 1488 | 75187 | Takeda 2016-01 | 27006499 | Gain | 7:133083253-133087647 | D | 12 |
| CTCF | 10664 | 4786 | Takeda 2016-01 | 27006499 | Loss | 8:105644211-105661790 | D | 8 |
| CTDSPL2 | 51496 | 32311 | Takeda 2016-01 | 27006499 | Loss | 2:122000618-122020312 | B | 5 |
| CTNNA1 | 1495 | 1433 | Takeda 2016-01 | 27006499 | Loss | 18:35238134-35251199 | B | 21 |
| CTNND1 | 1500 | 1017 | Takeda 2016-01 | 27006499 | N/A | 2:84600071-84650765 | D | 14 |
| CUL1 | 8454 | 2663 | Takeda 2016-01 | 27006499 | Loss | 6:47486448-47520175 | C | 16 |
| CUL3 | 8452 | 2661 | Takeda 2016-01 | 27006499 | Loss | 1:80256853-80342511 | A | 10 |
| DAP3 | 7818 | 3404 | Takeda 2016-01 | 27006499 | N/A | 3:88920803-88951181 | D | 6 |
| DCAF8 | 50717 | 56725 | Takeda 2016-01 | 27006499 | Loss | 1:172157767-172178443 | C | 3 |
| DCP1A | 55802 | 10178 | Takeda 2016-01 | 27006499 | Loss | 14:30478517-30517391 | C | 4 |
| DCUN1D3 | 123879 | 16155 | Takeda 2016-01 | 27006499 | N/A | 7:119852796-119896298 | D | 5 |
| DENND1B | 163486 | 11739 | Takeda 2016-01 | 27006499 | Loss | 1:139054768-139088736 | A | 3 |
| DGKD | 8527 | 100054 | Takeda 2016-01 | 27006499 | Loss | 1:87850179-87882670 | C | 4 |
| DHX9 | 1660 | 1039 | Takeda 2016-01 | 27006499 | Loss | 1:153465308-153468262 | D | 4 |
| DICER1 | 23405 | 13251 | Takeda 2016-01 | 27006499 | Loss | 12:104722099-104746922 | D | 6 |
| DIP2B | 57609 | 72227 | Takeda 2016-01 | 27006499 | Loss | 15:100028301-100094074 | B | 9 |
| DLG1 | 1739 | 20869 | Takeda 2016-01 | 27006499 | Loss | 16:31664091-31740558 | A | 14 |
| DLG2 | 1740 | 1046 | Takeda 2016-01 | 27006499 | N/A | 7:91090706-92449247 | C | 3 |
| DMXL1 | 1657 | 21136 | Takeda 2016-01 | 27006499 | Loss | 18:49910240-49971271 | B | 13 |
| DNAJA2 | 10294 | 21193 | Takeda 2016-01 | 27006499 | N/A | 8:85537640-85555271 | D | 5 |
| DNAJB1 | 3337 | 55957 | Takeda 2016-01 | 27006499 | N/A | 8:83608175-83611902 | D | 1 |
| DNAJB4 | 11080 | 100610 | Takeda 2016-01 | 27006499 | N/A | 3:152178511-152210302 | D | 5 |
| DNAJC1 | 64215 | 7293 | Takeda 2016-01 | 27006499 | Loss | 2:18372601-18379977 | B | 13 |
| DNASE2B | 58511 | 1052 | Takeda 2016-01 | 27006499 | Loss | 3:146476604-146486422 | C | 5 |
| DNM1L | 10059 | 6384 | Takeda 2016-01 | 27006499 | Loss | 16:16320087-16348942 | D | 7 |
| DNMT3A | 1788 | 7294 | Takeda 2016-01 | 27006499 | Loss | 12:3818819-3858245 | C | 8 |
| DPM1 | 8813 | 2865 | Takeda 2016-01 | 27006499 | Loss | 2:168183731-168195534 | D | 10 |
| DPP10 | 57628 | 41400 | Takeda 2016-01 | 27006499 | Loss | 1:123977393-124036468 | A | 7 |
| DPP3 | 10072 | 40210 | Takeda 2016-01 | 27006499 | N/A | 19:4907229-4928287 | D | 3 |
| DYRK1A | 1859 | 55576 | Takeda 2016-01 | 27006499 | Loss | 16:94583083-94636466 | B | 28 |
| EDEM1 | 9695 | 33836 | Takeda 2016-01 | 27006499 | N/A | 6:108828641-108859356 | D | 3 |
| EHD1 | 10938 | 81678 | Takeda 2016-01 | 27006499 | N/A | 19:6276725-6300096 | D | 3 |
| EHMT1 | 79813 | 11698 | Takeda 2016-01 | 27006499 | Loss | 2:24830994-24899926 | C | 14 |
| EIF2AK3 | 9451 | 3557 | Takeda 2016-01 | 27006499 | Loss | 6:70842258-70871587 | D | 3 |
| EIF2S1 | 1965 | 3020 | Takeda 2016-01 | 27006499 | Loss | 12:78869095-78888077 | C | 8 |
| EIF3E | 3646 | 1205 | Takeda 2016-01 | 27006499 | Loss | 15:43244151-43264540 | D | 5 |
| EIF4E | 1977 | 123817 | Takeda 2016-01 | 27006499 | Loss | 3:138523282-138561520 | D | 9 |
| EIF4E2 | 9470 | 128466 | Takeda 2016-01 | 27006499 | N/A | 1:87213914-87240488 | D | 2 |
| EIF4ENIF1 | 56478 | 10522 | Takeda 2016-01 | 27006499 | N/A | 11:3202392-3244588 | C | 13 |
| EIF4G1 | 1981 | 110725 | Takeda 2016-01 | 27006499 | N/A | 16:20668313-20692884 | D | 5 |
| EIF4G3 | 8672 | 2789 | Takeda 2016-01 | 27006499 | Loss | 4:138001436-138023435 | B | 6 |
| ELAVL1 | 1994 | 20367 | Takeda 2016-01 | 27006499 | Loss | 8:4278851-4318403 | C | 18 |
| ELF2 | 1998 | 5006 | Takeda 2016-01 | 27006499 | Loss | 3:51257789-51284278 | D | 13 |
| ELL | 8178 | 4762 | Takeda 2016-01 | 27006499 | N/A | 8:70539675-70592858 | D | 10 |
| ELMO1 | 9844 | 56685 | Takeda 2016-01 | 27006499 | Loss | 13:20181837-20255049 | C | 5 |
| EP400 | 57634 | 38779 | Takeda 2016-01 | 27006499 | Loss | 5:110695576-110744525 | C | 13 |
| EPC1 | 80314 | 32627 | Takeda 2016-01 | 27006499 | Loss | 18:6454950-6475273 | D | 18 |
| EPC2 | 26122 | 32274 | Takeda 2016-01 | 27006499 | Loss | 2:49490588-49523046 | B | 17 |
| EPS8 | 2059 | 3272 | Takeda 2016-01 | 27006499 | Loss | 6:137577556-137592241 | C | 6 |
| ERBB2IP | 55914 | 41282 | Takeda 2016-01 | 27006499 | Loss | 13:103838127-103890675 | B | 22 |
| ERI3 | 79033 | 15403 | Takeda 2016-01 | 27006499 | N/A | 4:117550365-117674297 | D | 5 |
| ERP44 | 23071 | 12638 | Takeda 2016-01 | 27006499 | Loss | 4:48143825-48258290 | A | 9 |
| ESYT2 | 57488 | 32699 | Takeda 2016-01 | 27006499 | Loss | 12:116306925-116350765 | B | 7 |
| ETNK1 | 55500 | 10240 | Takeda 2016-01 | 27006499 | Loss | 6:143167983-143198778 | D | 7 |
| ETV2 | 2116 | 7308 | Takeda 2016-01 | 27006499 | N/A | 7:30633616-30635852 | D | 1 |
| ETV6 | 2120 | 37560 | Takeda 2016-01 | 27006499 | Loss | 6:134105374-134173907 | B | 15 |
| EVI2A | 2123 | 49234 | Takeda 2016-01 | 27006499 | N/A | 11:79526560-79530609 | D | 3 |
| EVI2B | 2124 | 48438 | Takeda 2016-01 | 27006499 | Loss | 11:79481355-79557410 | A | 6 |
| EVI2B | 2124 | 48438 | Takeda 2016-01 | 27006499 | Loss | 11:79481355-79557410 | A | 6 |
| EXOC2 | 55770 | 10122 | Takeda 2016-01 | 27006499 | Loss | 13:30879335-30882254 | B | 6 |
| EXOC6B | 23233 | 44781 | Takeda 2016-01 | 27006499 | Loss | 6:84808393-84914438 | A | 6 |
| EXT1 | 2131 | 30957 | Takeda 2016-01 | 27006499 | Loss | 15:53083123-53121023 | D | 8 |
| EZH1 | 2145 | 20458 | Takeda 2016-01 | 27006499 | N/A | 11:101191115-101226463 | D | 2 |
| FAF1 | 11124 | 5120 | Takeda 2016-01 | 27006499 | Loss | 4:109753698-109829962 | A | 12 |
| FAM103A1 | 83640 | 12088 | Takeda 2016-01 | 27006499 | Loss | 7:81752140-81825376 | B | 2 |
| FAM120A | 23196 | 8752 | Takeda 2016-01 | 27006499 | Loss | 13:48937937-48968591 | C | 17 |
| FAM135A | 57579 | 32665 | Takeda 2016-01 | 27006499 | Loss | 1:24032451-24047220 | D | 2 |
| FAM13B | 51306 | 9585 | Takeda 2016-01 | 27006499 | Loss | 18:34448943-34475099 | D | 14 |
| FAM168A | 23201 | 18702 | Takeda 2016-01 | 27006499 | Loss | 7:100680237-100768336 | B | 4 |
| FAM168B | 130074 | 18390 | Takeda 2016-01 | 27006499 | Loss | 1:34808930-34820750 | C | 5 |
| FAM178A | 55719 | 23077 | Takeda 2016-01 | 27006499 | Loss | 19:44960437-44977498 | C | 10 |
| FAM193A | 8603 | 2746 | Takeda 2016-01 | 27006499 | Loss | 5:34389166-34436090 | B | 15 |
| FAM210A | 125228 | 34981 | Takeda 2016-01 | 27006499 | Loss | 18:68262993-68294929 | C | 5 |
| FAM49B | 51571 | 9599 | Takeda 2016-01 | 27006499 | Loss | 15:63934138-63972038 | C | 6 |
| FAM53B | 9679 | 8776 | Takeda 2016-01 | 27006499 | Loss | 7:132740509-132758085 | D | 4 |
| FAR1 | 84188 | 41718 | Takeda 2016-01 | 27006499 | Loss | 7:113529647-113553096 | D | 5 |
| FARP1 | 10160 | 38098 | Takeda 2016-01 | 27006499 | Loss | 14:121269963-121281481 | D | 5 |
| FARP2 | 9855 | 8877 | Takeda 2016-01 | 27006499 | Loss | 1:93591044-93626606 | A | 2 |
| FARSB | 10056 | 4160 | Takeda 2016-01 | 27006499 | Loss | 1:78395361-78430823 | C | 2 |
| FBRS | 64319 | 79554 | Takeda 2016-01 | 27006499 | N/A | 7:127479592-127491711 | D | 2 |
| FBXL17 | 64839 | 79859 | Takeda 2016-01 | 27006499 | Loss | 17:63309889-63318604 | A | 5 |
| FBXO11 | 80204 | 11843 | Takeda 2016-01 | 27006499 | Loss | 17:88010392-88036537 | D | 15 |
| FBXO8 | 26269 | 8137 | Takeda 2016-01 | 27006499 | Loss | 8:56572674-56574139 | D | 3 |
| FBXW11 | 23291 | 76444 | Takeda 2016-01 | 27006499 | Loss | 11:32686769-32727722 | C | 12 |
| FBXW7 | 55294 | 117451 | Takeda 2016-01 | 27006499 | Loss | 3:84821802-84982110 | A | 18 |
| FCHO2 | 115548 | 9030 | Takeda 2016-01 | 27006499 | Loss | 13:98788777-98815051 | C | 11 |
| FCHSD2 | 9873 | 8887 | Takeda 2016-01 | 27006499 | Loss | 7:101122592-101231048 | B | 12 |
| FEM1C | 56929 | 10606 | Takeda 2016-01 | 27006499 | Loss | 18:46497071-46505781 | D | 7 |
| FGFBP3 | 143282 | 49899 | Takeda 2016-01 | 27006499 | N/A | 19:36917550-36919615 | D | 3 |
| FGFR1OP2 | 26127 | 9222 | Takeda 2016-01 | 27006499 | Loss | 6:146574494-146606756 | C | 7 |
| FGFR2 | 2263 | 22566 | Takeda 2016-01 | 27006499 | Loss | 7:132740509-132758085 | D | 6 |
| FHIT | 2272 | 21661 | Takeda 2016-01 | 27006499 | N/A | 14:9550092-11162035 | B | 6 |
| FILIP1L | 11259 | 37121 | Takeda 2016-01 | 27006499 | Loss | 16:57554805-57575004 | C | 5 |
| FMNL2 | 114793 | 70871 | Takeda 2016-01 | 27006499 | Loss | 2:52908088-52937611 | C | 8 |
| FMR1 | 2332 | 1531 | Takeda 2016-01 | 27006499 | Loss | X:68671681-68702125 | D | 5 |
| FNDC3A | 22862 | 8952 | Takeda 2016-01 | 27006499 | Loss | 14:72636283-72676627 | B | 7 |
| FNDC3B | 64778 | 11244 | Takeda 2016-01 | 27006499 | Loss | 3:27660094-27688038 | A | 12 |
| FNIP1 | 96459 | 28173 | Takeda 2016-01 | 27006499 | Loss | 11:54435816-54472381 | B | 8 |
| FNTA | 2339 | 1534 | Takeda 2016-01 | 27006499 | N/A | 8:25998729-26015601 | D | 1 |
| FRS2 | 10818 | 4846 | Takeda 2016-01 | 27006499 | Loss | 10:117119266-117130974 | D | 14 |
| FRYL | 285527 | 103956 | Takeda 2016-01 | 27006499 | Loss | 5:73124542-73177364 | B | 11 |
| FUBP1 | 8880 | 48253 | Takeda 2016-01 | 27006499 | Loss | 3:152202408-152242117 | C | 12 |
| FUBP3 | 8939 | 45954 | Takeda 2016-01 | 27006499 | Loss | 2:31581205-31607762 | D | 6 |
| FUT8 | 2530 | 9650 | Takeda 2016-01 | 27006499 | Loss | 12:77243884-77281850 | A | 5 |
| FXR1 | 8087 | 3573 | Takeda 2016-01 | 27006499 | Loss | 3:34023881-34057707 | C | 12 |
| GABPA | 2551 | 1543 | Takeda 2016-01 | 27006499 | N/A | 16:84834925-84863779 | D | 4 |
| GALE | 2582 | 347 | Takeda 2016-01 | 27006499 | N/A | 4:135963727-135968178 | D | 3 |
| GALNT2 | 2590 | 3297 | Takeda 2016-01 | 27006499 | Loss | 8:122927105-122997420 | B | 5 |
| GALNT7 | 51809 | 9685 | Takeda 2016-01 | 27006499 | Loss | 8:57555622-57587850 | B | 7 |
| GALR2 | 8811 | 2863 | Takeda 2016-01 | 27006499 | N/A | 11:116280939-116283938 | D | 3 |
| GARS | 2617 | 1547 | Takeda 2016-01 | 27006499 | Loss | 6:55042958-55078153 | D | 1 |
| GATAD2B | 57459 | 32484 | Takeda 2016-01 | 27006499 | Loss | 3:90303159-90360517 | C | 13 |
| GBF1 | 8729 | 37897 | Takeda 2016-01 | 27006499 | Loss | 19:46201607-46208716 | C | 6 |
| GCC2 | 9648 | 45639 | Takeda 2016-01 | 27006499 | Loss | 10:58278458-58290167 | C | 1 |
| GDI2 | 2665 | 37488 | Takeda 2016-01 | 27006499 | Loss | 13:3548916-3569352 | D | 13 |
| GDPGP1 | 390637 | 33436 | Takeda 2016-01 | 27006499 | N/A | 7:80232865-80242061 | D | 2 |
| GGACT | 87769 | 15522 | Takeda 2016-01 | 27006499 | Loss | 14:122869761-122908223 | C | 6 |
| GIGYF2 | 26058 | 41048 | Takeda 2016-01 | 27006499 | Loss | 1:87411548-87451424 | B | 8 |
| GLTSCR1 | 29998 | 9250 | Takeda 2016-01 | 27006499 | Gain | 7:16004414-16024920 | D | 4 |
| GLTSCR2 | 29997 | 44130 | Takeda 2016-01 | 27006499 | N/A | 7:15936183-15946074 | D | 1 |
| GLUD1 | 2746 | 55885 | Takeda 2016-01 | 27006499 | Loss | 14:34309755-34329912 | D | 6 |
| GLYR1 | 84656 | 12525 | Takeda 2016-01 | 27006499 | N/A | 16:5013906-5049910 | D | 8 |
| GMDS | 2762 | 75968 | Takeda 2016-01 | 27006499 | Loss | 13:31870459-31889435 | A | 13 |
| GMFG | 9535 | 37978 | Takeda 2016-01 | 27006499 | N/A | 7:28437447-28448233 | D | 2 |
| GMFG | 9535 | 37978 | Takeda 2016-01 | 27006499 | N/A | 7:28437447-28448233 | D | 2 |
| GNAQ | 2776 | 1566 | Takeda 2016-01 | 27006499 | Loss | 19:16187033-16243990 | A | 11 |
| GNB1 | 2782 | 55532 | Takeda 2016-01 | 27006499 | Loss | 4:155502439-155546438 | D | 12 |
| GOLGA3 | 2802 | 4308 | Takeda 2016-01 | 27006499 | Loss | 5:110186646-110217440 | C | 2 |
| GOLIM4 | 27333 | 8716 | Takeda 2016-01 | 27006499 | N/A | 3:75875084-75956949 | D | 6 |
| GOLPH3 | 64083 | 56942 | Takeda 2016-01 | 27006499 | Loss | 15:12328905-12360945 | C | 11 |
| GON4L | 54856 | 13002 | Takeda 2016-01 | 27006499 | Loss | 3:88938335-89072170 | A | 3 |
| GPATCH2L | 55668 | 9942 | Takeda 2016-01 | 27006499 | N/A | 12:86241890-86291414 | D | 2 |
| GPATCH8 | 23131 | 46117 | Takeda 2016-01 | 27006499 | Loss | 11:102498724-102551378 | B | 12 |
| GPBP1 | 65056 | 11292 | Takeda 2016-01 | 27006499 | Loss | 13:111461164-111469929 | D | 16 |
| GPC5 | 2262 | 3285 | Takeda 2016-01 | 27006499 | N/A | 14:115092215-116525179 | B | 5 |
| GPLD1 | 2822 | 1152 | Takeda 2016-01 | 27006499 | N/A | 13:24943152-24990753 | D | 4 |
| GRAMD4 | 23151 | 18199 | Takeda 2016-01 | 27006499 | Loss | 15:86121247-86135823 | D | 6 |
| GRID2 | 2895 | 74399 | Takeda 2016-01 | 27006499 | N/A | 6:63255876-64668285 | C | 4 |
| GRM7 | 2917 | 20233 | Takeda 2016-01 | 27006499 | N/A | 6:110645581-111567230 | D | 6 |
| GSK3B | 2932 | 55629 | Takeda 2016-01 | 27006499 | Loss | 16:38168157-38198455 | B | 28 |
| GTF2B | 2959 | 1158 | Takeda 2016-01 | 27006499 | Loss | 3:142779533-142788357 | D | 3 |
| GTF2F2 | 2963 | 37884 | Takeda 2016-01 | 27006499 | Loss | 14:75979853-75994251 | D | 7 |
| GTF2IRD1 | 9569 | 4158 | Takeda 2016-01 | 27006499 | Loss | 5:134287039-134428411 | A | 7 |
| GTPBP1 | 9567 | 3165 | Takeda 2016-01 | 27006499 | N/A | 15:79690896-79721479 | D | 2 |
| GUCY1A3 | 2982 | 37360 | Takeda 2016-01 | 27006499 | Gain | 3:82094785-82102156 | C | 3 |
| GXYLT1 | 283464 | 28259 | Takeda 2016-01 | 27006499 | Loss | 15:93255162-93275569 | C | 4 |
| H2AFY | 9555 | 3598 | Takeda 2016-01 | 27006499 | Loss | 13:56107022-56118708 | D | 7 |
| H3F3A | 3020 | 134170 | Takeda 2016-01 | 27006499 | N/A | 1:180800832-180813943 | D | 4 |
| H3F3B | 3021 | 134170 | Takeda 2016-01 | 27006499 | N/A | 1:180800832-180813943 | D | 4 |
| HADHB | 3032 | 153 | Takeda 2016-01 | 27006499 | Loss | 5:30154231-30183558 | D | 7 |
| HARBI1 | 283254 | 24535 | Takeda 2016-01 | 27006499 | N/A | 2:91710852-91721545 | D | 2 |
| HBP1 | 26959 | 8171 | Takeda 2016-01 | 27006499 | Loss | 12:31923398-31933620 | D | 4 |
| HDAC2 | 3066 | 68187 | Takeda 2016-01 | 27006499 | Loss | 10:36987879-36992270 | D | 2 |
| HEATR5A | 25938 | 19635 | Takeda 2016-01 | 27006499 | Loss | 12:51912499-51932957 | D | 3 |
| HECTD1 | 25831 | 9115 | Takeda 2016-01 | 27006499 | Loss | 12:51757429-51826058 | A | 14 |
| HERC2 | 8924 | 3430 | Takeda 2016-01 | 27006499 | Loss | 7:56083842-56113208 | C | 4 |
| HERC4 | 26091 | 56715 | Takeda 2016-01 | 27006499 | Loss | 10:63289854-63326444 | C | 8 |
| HIPK2 | 28996 | 68766 | Takeda 2016-01 | 27006499 | Loss | 6:38837453-38865315 | C | 9 |
| HIPK3 | 10114 | 55923 | Takeda 2016-01 | 27006499 | N/A | 2:104426481-104494446 | D | 8 |
| HIRA | 7290 | 48172 | Takeda 2016-01 | 27006499 | Loss | 16:18932967-18956051 | C | 6 |
| HMBOX1 | 79618 | 11589 | Takeda 2016-01 | 27006499 | Loss | 14:64803076-64882503 | B | 9 |
| HMGCL | 3155 | 159 | Takeda 2016-01 | 27006499 | Loss | 4:135949648-135954048 | D | 3 |
| HMGCR | 3156 | 30994 | Takeda 2016-01 | 27006499 | N/A | 13:96648967-96670936 | D | 6 |
| HNRNPC | 3183 | 74524 | Takeda 2016-01 | 27006499 | Loss | 14:52077145-52098742 | D | 14 |
| HNRNPD | 3184 | 22410 | Takeda 2016-01 | 27006499 | Loss | 5:99949826-99968889 | D | 11 |
| HNRNPM | 4670 | 4354 | Takeda 2016-01 | 27006499 | Loss | 17:33650290-33680762 | C | 15 |
| HNRNPU | 3192 | 22991 | Takeda 2016-01 | 27006499 | Loss | 1:178314842-178329611 | D | 3 |
| HOMER2 | 9455 | 3560 | Takeda 2016-01 | 27006499 | Loss | 7:81652540-81656934 | D | 2 |
| HSPD1 | 3329 | 1626 | Takeda 2016-01 | 27006499 | Loss | 1:55070579-55083871 | D | 1 |
| HSPE1 | 3336 | 20500 | Takeda 2016-01 | 27006499 | N/A | 1:55088132-55091307 | D | 2 |
| HTT | 3064 | 1593 | Takeda 2016-01 | 27006499 | Loss | 5:34792421-34795354 | B | 3 |
| IER2 | 9592 | 3607 | Takeda 2016-01 | 27006499 | N/A | 8:84661331-84662852 | D | 3 |
| IFFO1 | 25900 | 18706 | Takeda 2016-01 | 27006499 | N/A | 6:125145241-125161782 | D | 2 |
| IGF1R | 3480 | 30997 | Takeda 2016-01 | 27006499 | Loss | 7:68073616-68097066 | B | 5 |
| IL1RAPL1 | 11141 | 8609 | Takeda 2016-01 | 27006499 | N/A | X:86740870-88115645 | B | 5 |
| IL1RAPL2 | 26280 | 9681 | Takeda 2016-01 | 27006499 | N/A | X:137570608-138846946 | C | 7 |
| IL6ST | 3572 | 1645 | Takeda 2016-01 | 27006499 | N/A | 13:112464070-112510086 | D | 3 |
| INADL | 10207 | 72199 | Takeda 2016-01 | 27006499 | Loss | 4:98459116-98576726 | A | 4 |
| INO80 | 54617 | 75070 | Takeda 2016-01 | 27006499 | Loss | 2:119426727-119530210 | B | 10 |
| INO80D | 54891 | 9819 | Takeda 2016-01 | 27006499 | Loss | 1:63045614-63085489 | B | 4 |
| INPP5A | 3632 | 4045 | Takeda 2016-01 | 27006499 | Loss | 7:139425758-139449208 | C | 4 |
| IPO7 | 10527 | 4659 | Takeda 2016-01 | 27006499 | Loss | 7:110020959-110044408 | B | 13 |
| IRF2 | 3660 | 1659 | Takeda 2016-01 | 27006499 | Loss | 8:46769966-46790490 | D | 15 |
| IST1 | 9798 | 8849 | Takeda 2016-01 | 27006499 | Loss | 8:109678547-109685871 | D | 9 |
| ITCH | 83737 | 88442 | Takeda 2016-01 | 27006499 | Loss | 2:155151945-155201182 | C | 12 |
| ITGA6 | 3655 | 20091 | Takeda 2016-01 | 27006499 | N/A | 2:71745616-71858416 | D | 7 |
| ITSN2 | 50618 | 22627 | Takeda 2016-01 | 27006499 | Loss | 12:4603445-4615127 | C | 9 |
| JARID2 | 3720 | 31279 | Takeda 2016-01 | 27006499 | Loss | 13:44844990-44869804 | C | 15 |
| JMJD1C | 221037 | 3129 | Takeda 2016-01 | 27006499 | Loss | 10:67118655-67207935 | B | 15 |
| KANSL1 | 284058 | 9140 | Takeda 2016-01 | 27006499 | Loss | 11:104329898-104432280 | A | 20 |
| KANSL1L | 151050 | 27376 | Takeda 2016-01 | 27006499 | Loss | 1:66737598-66781925 | C | 3 |
| KAT6A | 7994 | 4924 | Takeda 2016-01 | 27006499 | Loss | 8:22856638-22931348 | A | 19 |
| KBTBD4 | 55709 | 32320 | Takeda 2016-01 | 27006499 | N/A | 2:90904740-90911626 | D | 2 |
| KCNQ1 | 3784 | 85014 | Takeda 2016-01 | 27006499 | Loss | 7:143268601-143286189 | D | 11 |
| KDM1A | 23028 | 32240 | Takeda 2016-01 | 27006499 | Loss | 4:136484961-136589090 | A | 12 |
| KDM2A | 22992 | 56564 | Takeda 2016-01 | 27006499 | Loss | 19:4326540-4381988 | C | 13 |
| KDM3A | 55818 | 10196 | Takeda 2016-01 | 27006499 | Loss | 6:71584271-71606268 | C | 3 |
| KDM5A | 5927 | 3419 | Takeda 2016-01 | 27006499 | N/A | 6:120364124-120444574 | D | 10 |
| KDM6A | 7403 | 7586 | Takeda 2016-01 | 27006499 | Loss | X:18155452-18287374 | A | 27 |
| KHDRBS1 | 10657 | 4781 | Takeda 2016-01 | 27006499 | Loss | 4:129716955-129730155 | D | 10 |
| KIFC2 | 90990 | 7800 | Takeda 2016-01 | 27006499 | N/A | 15:76660641-76668196 | D | 5 |
| KLF13 | 51621 | 32288 | Takeda 2016-01 | 27006499 | N/A | 7:63886350-63887675 | D | 7 |
| KLF3 | 51274 | 7396 | Takeda 2016-01 | 27006499 | N/A | 5:64803388-64832901 | D | 10 |
| KLHL11 | 55175 | 10030 | Takeda 2016-01 | 27006499 | N/A | 11:100462614-100472741 | D | 3 |
| KLHL2 | 11275 | 21416 | Takeda 2016-01 | 27006499 | N/A | 8:64739863-64849818 | D | 4 |
| KLHL28 | 54813 | 23036 | Takeda 2016-01 | 27006499 | Loss | 12:64950400-64970843 | D | 2 |
| KMT2C | 58508 | 46480 | Takeda 2016-01 | 27006499 | Loss | 5:25292719-25408563 | A | 19 |
| KMT2E | 55904 | 18822 | Takeda 2016-01 | 27006499 | Loss | 5:23424547-23506664 | A | 18 |
| KPNA1 | 3836 | 55642 | Takeda 2016-01 | 27006499 | Loss | 16:35999654-36005425 | D | 5 |
| KPNA3 | 3839 | 20520 | Takeda 2016-01 | 27006499 | Loss | 14:61390440-61416356 | C | 8 |
| KPNA4 | 3840 | 20521 | Takeda 2016-01 | 27006499 | Loss | 3:69049848-69102793 | B | 14 |
| KPNB1 | 3837 | 1707 | Takeda 2016-01 | 27006499 | N/A | 11:97159714-97187881 | D | 9 |
| KRAS | 3845 | 37990 | Takeda 2016-01 | 27006499 | N/A | 6:145216699-145250239 | D | 8 |
| LARP1 | 23367 | 9089 | Takeda 2016-01 | 27006499 | N/A | 11:58009064-58062031 | D | 12 |
| LARP4B | 23185 | 18195 | Takeda 2016-01 | 27006499 | Loss | 13:9097167-9133659 | B | 19 |
| LAT2 | 7462 | 11297 | Takeda 2016-01 | 27006499 | N/A | 5:134600022-134615025 | D | 4 |
| LATS1 | 9113 | 55843 | Takeda 2016-01 | 27006499 | Loss | 10:7668878-7717177 | B | 13 |
| LCOR | 84458 | 18153 | Takeda 2016-01 | 27006499 | Loss | 19:41504212-41521273 | D | 12 |
| LCORL | 254251 | 82325 | Takeda 2016-01 | 27006499 | Loss | 5:45753857-45794941 | B | 8 |
| LGR4 | 55366 | 10226 | Takeda 2016-01 | 27006499 | Loss | 2:109961736-110022226 | B | 11 |
| LIMA1 | 51474 | 9484 | Takeda 2016-01 | 27006499 | Loss | 15:99796568-99812588 | D | 8 |
| LIN7B | 64130 | 22648 | Takeda 2016-01 | 27006499 | N/A | 7:45367892-45370564 | D | 5 |
| LMO7 | 4008 | 83924 | Takeda 2016-01 | 27006499 | Loss | 14:101840423-101864462 | B | 4 |
| LNPEP | 4012 | 21148 | Takeda 2016-01 | 27006499 | Loss | 17:17590006-17627734 | B | 9 |
| LNX2 | 222484 | 17737 | Takeda 2016-01 | 27006499 | N/A | 5:147016655-147076586 | D | 4 |
| LONP2 | 83752 | 12050 | Takeda 2016-01 | 27006499 | Loss | 8:86660711-86704730 | C | 5 |
| LPCAT3 | 10162 | 14678 | Takeda 2016-01 | 27006499 | Loss | 6:124670423-124692420 | D | 6 |
| LPHN2 | 23266 | 22712 | Takeda 2016-01 | 27006499 | Loss | 3:148882049-148955686 | B | 9 |
| LPP | 4026 | 4075 | Takeda 2016-01 | 27006499 | Loss | 16:24430697-24502905 | A | 14 |
| LRCH1 | 23143 | 32244 | Takeda 2016-01 | 27006499 | Loss | 14:74791824-74832168 | B | 9 |
| LRIG1 | 26018 | 7380 | Takeda 2016-01 | 27006499 | Loss | 6:94633593-94636526 | D | 14 |
| LRP1B | 53353 | 56810 | Takeda 2016-01 | 27006499 | N/A | 2:40595246-42653624 | A | 5 |
| LRP6 | 4040 | 1747 | Takeda 2016-01 | 27006499 | Loss | 6:134504292-134555617 | C | 11 |
| LRRC4 | 64101 | 36403 | Takeda 2016-01 | 27006499 | Gain | 6:28788018-28832010 | D | 4 |
| LRRC41 | 10489 | 4645 | Takeda 2016-01 | 27006499 | N/A | 4:116075269-116097043 | D | 9 |
| LSM14A | 26065 | 40537 | Takeda 2016-01 | 27006499 | Loss | 7:34374438-34394944 | D | 13 |
| LSR | 51599 | 9306 | Takeda 2016-01 | 27006499 | N/A | 7:30957770-30973464 | D | 5 |
| LUC7L | 55692 | 100558 | Takeda 2016-01 | 27006499 | Loss | 17:26257170-26271681 | D | 11 |
| LUC7L2 | 51631 | 56737 | Takeda 2016-01 | 27006499 | Loss | 6:38564696-38580827 | D | 15 |
| LUC7L3 | 51747 | 75056 | Takeda 2016-01 | 27006499 | Loss | 11:94305246-94325723 | D | 11 |
| LUZP1 | 7798 | 11545 | Takeda 2016-01 | 27006499 | Loss | 4:136484961-136589090 | A | 11 |
| LYN | 4067 | 55649 | Takeda 2016-01 | 27006499 | Loss | 4:3702308-3718441 | D | 2 |
| MACROD2 | 140733 | 85987 | Takeda 2016-01 | 27006499 | N/A | 2:140395309-142392966 | A | 4 |
| MAEA | 10296 | 4298 | Takeda 2016-01 | 27006499 | Loss | 5:33346567-33377361 | C | 13 |
| MAGI1 | 9223 | 31257 | Takeda 2016-01 | 27006499 | Loss | 6:94141125-94224343 | C | 13 |
| MAGI2 | 9863 | 8189 | Takeda 2016-01 | 27006499 | N/A | 5:19227036-20704792 | B | 3 |
| MAGI3 | 260425 | 26431 | Takeda 2016-01 | 27006499 | Loss | 3:104083262-104142171 | C | 13 |
| MAN1A2 | 10905 | 55982 | Takeda 2016-01 | 27006499 | Loss | 3:100582050-100629141 | C | 10 |
| MAN2A1 | 4124 | 1777 | Takeda 2016-01 | 27006499 | Loss | 17:64674253-64737277 | B | 4 |
| MAP3K2 | 10746 | 74576 | Takeda 2016-01 | 27006499 | Loss | 18:32172266-32204202 | C | 8 |
| MAP7 | 9053 | 20851 | Takeda 2016-01 | 27006499 | Loss | 10:20078750-20290571 | A | 11 |
| MAPK1 | 5594 | 37670 | Takeda 2016-01 | 27006499 | Loss | 16:16985210-17002523 | D | 14 |
| MAPK8 | 5599 | 56760 | Takeda 2016-01 | 27006499 | Loss | 14:33396939-33444451 | C | 14 |
| MAPKAP1 | 79109 | 11473 | Takeda 2016-01 | 27006499 | Loss | 2:34432225-34501157 | B | 6 |
| MAPKAPK2 | 9261 | 56412 | Takeda 2016-01 | 27006499 | N/A | 1:131053700-131097826 | D | 2 |
| MAST2 | 23139 | 7428 | Takeda 2016-01 | 27006499 | Loss | 4:116089411-116468227 | A | 7 |
| MAST4 | 375449 | 42094 | Takeda 2016-01 | 27006499 | Loss | 13:102772559-102788615 | C | 3 |
| MBD3 | 53615 | 2917 | Takeda 2016-01 | 27006499 | N/A | 10:80392539-80399550 | D | 2 |
| MBNL1 | 4154 | 23186 | Takeda 2016-01 | 27006499 | Loss | 3:60581467-60603528 | B | 18 |
| MBNL2 | 10150 | 76766 | Takeda 2016-01 | 27006499 | Loss | 14:120372871-120407452 | B | 11 |
| MBTPS2 | 51360 | 9287 | Takeda 2016-01 | 27006499 | Loss | X:157557468-157570516 | D | 2 |
| MCMBP | 79892 | 11749 | Takeda 2016-01 | 27006499 | Loss | 7:128707974-128713833 | D | 12 |
| MCU | 90550 | 9916 | Takeda 2016-01 | 27006499 | Loss | 10:59535698-59575215 | B | 10 |
| MECOM | 2122 | 21086 | Takeda 2016-01 | 27006499 | Loss | 3:29972059-29998532 | A | 16 |
| MED13 | 9969 | 21067 | Takeda 2016-01 | 27006499 | Loss | 11:86269084-86298388 | B | 12 |
| MED13L | 23389 | 25256 | Takeda 2016-01 | 27006499 | Loss | 5:118627825-118654236 | B | 14 |
| MED20 | 9477 | 3158 | Takeda 2016-01 | 27006499 | Loss | 17:47626838-47658761 | C | 2 |
| MEF2A | 4205 | 4080 | Takeda 2016-01 | 27006499 | Loss | 7:67283657-67312952 | B | 15 |
| MEIS2 | 4212 | 7846 | Takeda 2016-01 | 27006499 | Loss | 2:115917428-115952836 | A | 4 |
| METAP1 | 23173 | 6488 | Takeda 2016-01 | 27006499 | N/A | 3:138458956-138489515 | D | 4 |
| MGA | 23269 | 49351 | Takeda 2016-01 | 27006499 | Loss | 2:119896471-119940732 | B | 9 |
| MIB1 | 57534 | 10810 | Takeda 2016-01 | 27006499 | Loss | 18:10779392-10804069 | B | 7 |
| MID1 | 4281 | 7837 | Takeda 2016-01 | 27006499 | Loss | X:169892940-169910337 | D | 7 |
| MINK1 | 50488 | 56762 | Takeda 2016-01 | 27006499 | Loss | 11:70582905-70594606 | D | 6 |
| MKL1 | 57591 | 32487 | Takeda 2016-01 | 27006499 | N/A | 15:81012281-81190757 | C | 13 |
| MKL2 | 57496 | 40917 | Takeda 2016-01 | 27006499 | Loss | 16:13358053-13372481 | D | 10 |
| MKLN1 | 4289 | 8305 | Takeda 2016-01 | 27006499 | Loss | 6:31470115-31502377 | A | 17 |
| MLLT10 | 8028 | 20973 | Takeda 2016-01 | 27006499 | N/A | 2:18064394-18212388 | A | 19 |
| MLLT3 | 4300 | 37933 | Takeda 2016-01 | 27006499 | Loss | 4:87916243-87955407 | B | 7 |
| MOAP1 | 64112 | 11154 | Takeda 2016-01 | 27006499 | Loss | 12:102687828-102746341 | B | 2 |
| MOB4 | 25843 | 9116 | Takeda 2016-01 | 27006499 | Loss | 1:55153284-55156237 | D | 2 |
| MOCS3 | 27304 | 6108 | Takeda 2016-01 | 27006499 | N/A | 2:168230622-168232594 | D | 4 |
| MON2 | 23041 | 44309 | Takeda 2016-01 | 27006499 | Loss | 10:123046881-123051272 | D | 6 |
| MPP5 | 64398 | 9512 | Takeda 2016-01 | 27006499 | Loss | 12:78766880-78822368 | B | 8 |
| MRPL30 | 51263 | 32675 | Takeda 2016-01 | 27006499 | N/A | 1:37890477-37898535 | D | 1 |
| MRPL40 | 64976 | 2800 | Takeda 2016-01 | 27006499 | N/A | 16:18872018-18876767 | D | 3 |
| MSI2 | 124540 | 62199 | Takeda 2016-01 | 27006499 | Loss | 11:88409177-88417960 | A | 8 |
| MTM1 | 4534 | 37279 | Takeda 2016-01 | 27006499 | Loss | X:71240059-71269091 | D | 2 |
| MTMR12 | 54545 | 10403 | Takeda 2016-01 | 27006499 | N/A | 15:12205028-12272240 | D | 5 |
| MTMR3 | 8897 | 23662 | Takeda 2016-01 | 27006499 | Loss | 11:4515658-4562497 | B | 12 |
| MTPN | 136319 | 40607 | Takeda 2016-01 | 27006499 | Loss | 6:35501327-35536521 | C | 10 |
| MTUS1 | 57509 | 100292 | Takeda 2016-01 | 27006499 | Loss | 8:41058239-41084607 | C | 9 |
| MYH9 | 4627 | 129835 | Takeda 2016-01 | 27006499 | Loss | 15:77770252-77821225 | D | 9 |
| MYO10 | 4651 | 36328 | Takeda 2016-01 | 27006499 | Loss | 15:25744695-25803074 | B | 10 |
| MYO1B | 4430 | 7856 | Takeda 2016-01 | 27006499 | Loss | 1:51863006-51901423 | C | 3 |
| NAP1L1 | 4673 | 129218 | Takeda 2016-01 | 27006499 | N/A | 10:111473192-111498142 | D | 3 |
| NAV1 | 89796 | 10719 | Takeda 2016-01 | 27006499 | Gain | 1:135418310-135513492 | A | 2 |
| NCOA2 | 10499 | 4768 | Takeda 2016-01 | 27006499 | Gain | 1:13300543-13338941 | B | 8 |
| NCOA3 | 8202 | 4764 | Takeda 2016-01 | 27006499 | Loss | 2:166035569-166050330 | D | 9 |
| NCOA5 | 57727 | 32496 | Takeda 2016-01 | 27006499 | Loss | 2:165017611-165036790 | D | 8 |
| NDUFA7 | 4701 | 37994 | Takeda 2016-01 | 27006499 | N/A | 17:33824591-33838316 | D | 2 |
| NDUFB9 | 4715 | 3669 | Takeda 2016-01 | 27006499 | N/A | 15:58933810-58939489 | D | 3 |
| NDUFS3 | 4722 | 3346 | Takeda 2016-01 | 27006499 | N/A | 2:90894634-90904827 | D | 4 |
| NDUFS4 | 4724 | 1866 | Takeda 2016-01 | 27006499 | Loss | 13:114287861-114302457 | C | 9 |
| NF1 | 4763 | 226 | Takeda 2016-01 | 27006499 | Loss | 11:79481355-79557410 | A | 25 |
| NFAT5 | 10725 | 4811 | Takeda 2016-01 | 27006499 | Loss | 8:107349355-107368399 | D | 9 |
| NFATC3 | 4775 | 27827 | Takeda 2016-01 | 27006499 | Loss | 8:106085908-106115227 | C | 13 |
| NFIA | 4774 | 4086 | Takeda 2016-01 | 27006499 | Loss | 4:97842973-97867450 | C | 16 |
| NFS1 | 9054 | 5463 | Takeda 2016-01 | 27006499 | Loss | 2:156082601-156113583 | D | 8 |
| NGLY1 | 55768 | 10117 | Takeda 2016-01 | 27006499 | Loss | 14:16280553-16289198 | D | 4 |
| NHLRC2 | 374354 | 12048 | Takeda 2016-01 | 27006499 | Loss | 19:56561752-56580234 | D | 7 |
| NIP7 | 51388 | 56743 | Takeda 2016-01 | 27006499 | N/A | 8:107056877-107060926 | D | 2 |
| NIPBL | 25836 | 15850 | Takeda 2016-01 | 27006499 | Loss | 15:8373516-8402644 | B | 16 |
| NLRC4 | 58484 | 10924 | Takeda 2016-01 | 27006499 | Loss | 17:74270588-74482638 | A | 2 |
| NMD3 | 51068 | 6127 | Takeda 2016-01 | 27006499 | Loss | 3:69726377-69754320 | D | 4 |
| NOP2 | 4839 | 135865 | Takeda 2016-01 | 27006499 | N/A | 6:125131909-125144753 | D | 5 |
| NOP58 | 51602 | 7024 | Takeda 2016-01 | 27006499 | N/A | 1:59684971-59719044 | D | 3 |
| NR2C2 | 7182 | 2475 | Takeda 2016-01 | 27006499 | Loss | 6:92121601-92209587 | B | 11 |
| NR3C1 | 2908 | 30960 | Takeda 2016-01 | 27006499 | Loss | 18:39436283-39463865 | C | 18 |
| NRF1 | 4899 | 3674 | Takeda 2016-01 | 27006499 | Loss | 6:30087273-30100471 | C | 5 |
| NRIP1 | 8204 | 2606 | Takeda 2016-01 | 27006499 | Loss | 16:76316624-76377221 | B | 13 |
| NSD1 | 64324 | 32543 | Takeda 2016-01 | 27006499 | Loss | 13:55227607-55245137 | B | 18 |
| NUMA1 | 4926 | 38150 | Takeda 2016-01 | 27006499 | Loss | 7:101981444-102010756 | C | 8 |
| OGDH | 4967 | 55662 | Takeda 2016-01 | 27006499 | N/A | 11:6291633-6356642 | D | 6 |
| OMG | 4974 | 36099 | Takeda 2016-01 | 27006499 | N/A | 11:79500982-79504084 | D | 2 |
| ORC4 | 5000 | 8059 | Takeda 2016-01 | 27006499 | Loss | 2:48881265-48931427 | C | 5 |
| ORMDL2 | 29095 | 39087 | Takeda 2016-01 | 27006499 | N/A | 10:128817457-128821631 | D | 3 |
| P4HB | 5034 | 55495 | Takeda 2016-01 | 27006499 | N/A | 11:120560298-120573253 | D | 5 |
| PACS1 | 55690 | 9970 | Takeda 2016-01 | 27006499 | Loss | 19:5235766-5257226 | B | 3 |
| PACSIN2 | 11252 | 21414 | Takeda 2016-01 | 27006499 | Loss | 15:83396254-83448684 | B | 7 |
| PAFAH1B1 | 5048 | 371 | Takeda 2016-01 | 27006499 | Loss | 11:74664803-74708715 | C | 16 |
| PAG1 | 55824 | 10198 | Takeda 2016-01 | 27006499 | Loss | 3:9720517-9755959 | B | 5 |
| PAIP2 | 51247 | 22978 | Takeda 2016-01 | 27006499 | Gain | 18:35587980-35602497 | D | 8 |
| PAK2 | 5062 | 99711 | Takeda 2016-01 | 27006499 | Loss | 16:32040657-32075284 | C | 9 |
| PAN3 | 255967 | 6372 | Takeda 2016-01 | 27006499 | Loss | 5:147446138-147468134 | C | 15 |
| PANK3 | 79646 | 56999 | Takeda 2016-01 | 27006499 | N/A | 11:35769484-35791285 | D | 5 |
| PAPOLA | 10914 | 23389 | Takeda 2016-01 | 27006499 | Loss | 12:105791526-105835366 | D | 11 |
| PARD3 | 56288 | 10489 | Takeda 2016-01 | 27006499 | Loss | 8:127222304-127432619 | A | 18 |
| PARG | 8505 | 50532 | Takeda 2016-01 | 27006499 | N/A | 14:32201949-32297550 | C | 6 |
| PARN | 5073 | 31098 | Takeda 2016-01 | 27006499 | Loss | 16:13561972-13573525 | C | 2 |
| PARVA | 55742 | 10077 | Takeda 2016-01 | 27006499 | Loss | 7:112497724-112747335 | A | 5 |
| PATL1 | 219988 | 82269 | Takeda 2016-01 | 27006499 | N/A | 19:11912399-11945096 | D | 3 |
| PBRM1 | 55193 | 10044 | Takeda 2016-01 | 27006499 | Loss | 14:31073144-31083222 | B | 9 |
| PBX1 | 5087 | 20574 | Takeda 2016-01 | 27006499 | Loss | 1:168167262-168210091 | A | 2 |
| PCBP2 | 5094 | 74536 | Takeda 2016-01 | 27006499 | Loss | 15:102476310-102506894 | C | 11 |
| PCCA | 5095 | 236 | Takeda 2016-01 | 27006499 | Loss | 14:122869761-122908223 | C | 6 |
| PCED1A | 64773 | 11241 | Takeda 2016-01 | 27006499 | N/A | 2:130417247-130424701 | D | 2 |
| PCGF3 | 10336 | 4605 | Takeda 2016-01 | 27006499 | Loss | 5:108460713-108488574 | D | 9 |
| PCM1 | 5108 | 4518 | Takeda 2016-01 | 27006499 | Loss | 8:41239886-41260395 | D | 9 |
| PCNX | 22990 | 40997 | Takeda 2016-01 | 27006499 | Loss | 12:81887091-81921224 | C | 10 |
| PCYT2 | 5833 | 2143 | Takeda 2016-01 | 27006499 | N/A | 11:120610087-120617936 | D | 2 |
| PDCD10 | 11235 | 10505 | Takeda 2016-01 | 27006499 | Loss | 3:75513643-75559235 | B | 7 |
| PDE3B | 5140 | 709 | Takeda 2016-01 | 27006499 | Loss | 7:114456269-114476775 | D | 5 |
| PDE8A | 5151 | 1957 | Takeda 2016-01 | 27006499 | Loss | 7:81291810-81311368 | D | 2 |
| PDF | 64146 | 69354 | Takeda 2016-01 | 27006499 | N/A | 8:107044698-107048614 | D | 2 |
| PDS5A | 23244 | 22877 | Takeda 2016-01 | 27006499 | Loss | 5:65618523-65672780 | C | 16 |
| PEX5 | 5830 | 270 | Takeda 2016-01 | 27006499 | N/A | 6:124396817-124415067 | D | 2 |
| PHACTR4 | 65979 | 41537 | Takeda 2016-01 | 27006499 | Loss | 4:132385786-132388719 | D | 5 |
| PHB2 | 11331 | 5263 | Takeda 2016-01 | 27006499 | N/A | 6:124712336-124716950 | D | 3 |
| PHC3 | 80012 | 69390 | Takeda 2016-01 | 27006499 | Loss | 3:30923612-30954497 | D | 5 |
| PHF14 | 9678 | 8775 | Takeda 2016-01 | 27006499 | Loss | 6:12013543-12079532 | A | 6 |
| PHF3 | 23469 | 9040 | Takeda 2016-01 | 27006499 | Loss | 1:30818881-30875002 | B | 3 |
| PHKA1 | 5255 | 1981 | Takeda 2016-01 | 27006499 | Loss | X:102540801-102550949 | D | 2 |
| PHTF1 | 10745 | 4817 | Takeda 2016-01 | 27006499 | N/A | 3:103968110-104024598 | D | 3 |
| PIBF1 | 10464 | 4628 | Takeda 2016-01 | 27006499 | Loss | 14:99085549-99148050 | B | 3 |
| PICALM | 8301 | 111783 | Takeda 2016-01 | 27006499 | Loss | 7:90175038-90213121 | C | 24 |
| PIK3CA | 5290 | 21249 | Takeda 2016-01 | 27006499 | Loss | 3:32423742-32441391 | D | 6 |
| PIK3R1 | 5295 | 7889 | Takeda 2016-01 | 27006499 | Loss | 13:101700842-101747552 | C | 17 |
| PIP5K1B | 8395 | 100644 | Takeda 2016-01 | 27006499 | Loss | 19:24189226-24396674 | A | 7 |
| PJA2 | 9867 | 32233 | Takeda 2016-01 | 27006499 | Loss | 17:64297593-64312118 | C | 8 |
| PLCD4 | 84812 | 88782 | Takeda 2016-01 | 27006499 | Loss | 1:74471394-74599528 | B | 2 |
| PLD1 | 5337 | 116234 | Takeda 2016-01 | 27006499 | Loss | 3:27964876-28056114 | B | 4 |
| PLEKHA5 | 54477 | 10377 | Takeda 2016-01 | 27006499 | Loss | 6:140543446-140572848 | B | 9 |
| PLEKHG3 | 26030 | 77478 | Takeda 2016-01 | 27006499 | Loss | 12:76559047-76576570 | D | 8 |
| PLXDC2 | 84898 | 41666 | Takeda 2016-01 | 27006499 | Loss | 2:16467169-16585619 | A | 3 |
| POGZ | 23126 | 9022 | Takeda 2016-01 | 27006499 | Loss | 3:94641182-95115939 | A | 8 |
| POLR1D | 51082 | 22239 | Takeda 2016-01 | 27006499 | Loss | 5:147069011-147077815 | D | 3 |
| POMK | 84197 | 12580 | Takeda 2016-01 | 27006499 | N/A | 8:25980604-25994121 | D | 1 |
| PPIH | 10465 | 38172 | Takeda 2016-01 | 27006499 | N/A | 4:119293733-119313315 | C | 3 |
| PPIL4 | 85313 | 12126 | Takeda 2016-01 | 27006499 | N/A | 10:7792891-7823135 | D | 4 |
| PPM1A | 5494 | 56428 | Takeda 2016-01 | 27006499 | Loss | 12:72765429-72796093 | C | 11 |
| PPM1B | 5495 | 2027 | Takeda 2016-01 | 27006499 | Loss | 17:84971412-85003335 | C | 15 |
| PPP1R12A | 4659 | 1855 | Takeda 2016-01 | 27006499 | Loss | 10:108166371-108201498 | B | 23 |
| PPP1R13B | 23368 | 9090 | Takeda 2016-01 | 27006499 | Loss | 12:111846448-111859590 | D | 10 |
| PPP2CA | 5515 | 37660 | Takeda 2016-01 | 27006499 | Loss | 11:52108822-52121985 | D | 10 |
| PPP2R2A | 5520 | 2035 | Takeda 2016-01 | 27006499 | Loss | 14:67008438-67070348 | B | 17 |
| PPP2R5A | 5525 | 55961 | Takeda 2016-01 | 27006499 | Loss | 1:191376206-191385067 | D | 3 |
| PPP2R5E | 5529 | 55962 | Takeda 2016-01 | 27006499 | Loss | 12:75501857-75529601 | B | 17 |
| PPP3CA | 5530 | 55497 | Takeda 2016-01 | 27006499 | Loss | 3:136820459-136928459 | A | 10 |
| PPP3CB | 5532 | 56429 | Takeda 2016-01 | 27006499 | Loss | 14:20510686-20542361 | D | 5 |
| PPP4R1 | 9989 | 81737 | Takeda 2016-01 | 27006499 | Loss | 17:65782042-65805282 | C | 11 |
| PPP4R2 | 151987 | 17953 | Takeda 2016-01 | 27006499 | Loss | 6:100827787-100842451 | D | 9 |
| PPP6R3 | 55291 | 115911 | Takeda 2016-01 | 27006499 | Loss | 19:3457863-3578710 | A | 22 |
| PPRC1 | 23082 | 9006 | Takeda 2016-01 | 27006499 | N/A | 19:46044886-46072915 | D | 2 |
| PRADC1 | 84279 | 13008 | Takeda 2016-01 | 27006499 | N/A | 6:85446810-85451970 | D | 4 |
| PRDM16 | 63976 | 11139 | Takeda 2016-01 | 27006499 | Loss | 4:154380351-154395026 | D | 12 |
| PRELID1 | 27166 | 40859 | Takeda 2016-01 | 27006499 | N/A | 13:55322055-55325271 | D | 4 |
| PRKCA | 5578 | 55679 | Takeda 2016-01 | 27006499 | Loss | 11:108204546-108224082 | D | 12 |
| PROSER2 | 254427 | 51648 | Takeda 2016-01 | 27006499 | N/A | 2:6097607-6130211 | D | 3 |
| PRRC2B | 84726 | 106649 | Takeda 2016-01 | 27006499 | N/A | 2:32151082-32234537 | D | 8 |
| PSAP | 5660 | 37680 | Takeda 2016-01 | 27006499 | Loss | 10:60276284-60296774 | D | 4 |
| PSD3 | 23362 | 87257 | Takeda 2016-01 | 27006499 | Loss | 8:67823418-67836602 | C | 8 |
| PSMC6 | 5706 | 100630 | Takeda 2016-01 | 27006499 | N/A | 14:45310780-45336716 | C | 5 |
| PSMD1 | 5707 | 2100 | Takeda 2016-01 | 27006499 | Loss | 1:86082364-86089749 | C | 3 |
| PSPC1 | 55269 | 10108 | Takeda 2016-01 | 27006499 | Loss | 14:56677435-56754360 | B | 11 |
| PTBP2 | 58155 | 23162 | Takeda 2016-01 | 27006499 | Loss | 3:119761118-119772884 | D | 8 |
| PTBP3 | 9991 | 55872 | Takeda 2016-01 | 27006499 | Loss | 4:59478293-59545757 | B | 12 |
| PTEN | 5728 | 265 | Takeda 2016-01 | 27006499 | Loss | 19:32764840-32828817 | A | 45 |
| PTPN12 | 5782 | 37691 | Takeda 2016-01 | 27006499 | Loss | 5:21005015-21053406 | B | 12 |
| PTPRA | 5786 | 20621 | Takeda 2016-01 | 27006499 | Loss | 2:130444777-130484167 | C | 6 |
| PTPRC | 5788 | 2126 | Takeda 2016-01 | 27006499 | Loss | 1:138121714-138127624 | C | 3 |
| PTPRK | 5796 | 55693 | Takeda 2016-01 | 27006499 | Loss | 10:28176955-28232572 | A | 13 |
| PUM1 | 9698 | 22830 | Takeda 2016-01 | 27006499 | Loss | 4:130705051-130746116 | B | 20 |
| PUM2 | 23369 | 69183 | Takeda 2016-01 | 27006499 | Loss | 12:8678883-8753354 | A | 18 |
| PVRL3 | 25945 | 9162 | Takeda 2016-01 | 27006499 | N/A | 16:46387706-46498525 | C | 5 |
| PXK | 54899 | 9828 | Takeda 2016-01 | 27006499 | Loss | 14:8116779-8129737 | D | 4 |
| R3HCC1L | 27291 | 8707 | Takeda 2016-01 | 27006499 | Loss | 19:42523592-42550605 | C | 8 |
| RAB14 | 51552 | 48679 | Takeda 2016-01 | 27006499 | Loss | 2:35185507-35191409 | D | 11 |
| RAB21 | 23011 | 8991 | Takeda 2016-01 | 27006499 | Loss | 10:115286827-115307318 | D | 1 |
| RAB2A | 5862 | 20628 | Takeda 2016-01 | 27006499 | Loss | 4:8537722-8616919 | B | 19 |
| RAB40C | 57799 | 10907 | Takeda 2016-01 | 27006499 | N/A | 17:25882114-25919727 | D | 8 |
| RAB5A | 5868 | 68142 | Takeda 2016-01 | 27006499 | Loss | 17:53475111-53499779 | D | 4 |
| RAB5B | 5869 | 104027 | Takeda 2016-01 | 27006499 | Loss | 10:128684701-128692020 | D | 1 |
| RABGAP1 | 23637 | 49301 | Takeda 2016-01 | 27006499 | Loss | 2:37423536-37519628 | B | 4 |
| RABGAP1L | 9910 | 8143 | Takeda 2016-01 | 27006499 | Loss | 1:160248280-160297017 | B | 8 |
| RAF1 | 5894 | 48145 | Takeda 2016-01 | 27006499 | N/A | 6:115618067-115676635 | D | 24 |
| RAI1 | 10743 | 7508 | Takeda 2016-01 | 27006499 | Loss | 11:60122400-60147264 | D | 12 |
| RALGAPA1 | 253959 | 84805 | Takeda 2016-01 | 27006499 | Loss | 12:55748175-55770078 | A | 5 |
| RALGAPA2 | 57186 | 28131 | Takeda 2016-01 | 27006499 | Loss | 2:146369293-146494951 | A | 10 |
| RALGAPB | 57148 | 10666 | Takeda 2016-01 | 27006499 | Loss | 2:158412192-158432847 | D | 6 |
| RALY | 22913 | 7216 | Takeda 2016-01 | 27006499 | Loss | 2:154805058-154861150 | C | 17 |
| RANBP9 | 10048 | 38057 | Takeda 2016-01 | 27006499 | Loss | 13:43430558-43472889 | C | 15 |
| RAPGEF6 | 51735 | 22968 | Takeda 2016-01 | 27006499 | Loss | 11:54660124-54718808 | B | 9 |
| RARA | 5914 | 20262 | Takeda 2016-01 | 27006499 | N/A | 11:98927818-98974942 | D | 3 |
| RASA1 | 5921 | 2168 | Takeda 2016-01 | 27006499 | Loss | 13:85204746-85283568 | A | 16 |
| RB1CC1 | 9821 | 7659 | Takeda 2016-01 | 27006499 | Loss | 1:6217470-6244053 | C | 3 |
| RBFOX1 | 54715 | 69339 | Takeda 2016-01 | 27006499 | N/A | 16:6809222-7412479 | C | 4 |
| RBFOX2 | 23543 | 49375 | Takeda 2016-01 | 27006499 | Loss | 15:77231391-77275082 | A | 13 |
| RBM12 | 10137 | 34993 | Takeda 2016-01 | 27006499 | Gain | 2:156082601-156113583 | D | 7 |
| RBM26 | 64062 | 41468 | Takeda 2016-01 | 27006499 | Loss | 14:105140878-105249188 | B | 11 |
| RBM47 | 54502 | 36932 | Takeda 2016-01 | 27006499 | Loss | 5:66022896-66034634 | C | 10 |
| RBM4B | 83759 | 57033 | Takeda 2016-01 | 27006499 | Loss | 19:4751637-4785759 | C | 6 |
| RC3H1 | 149041 | 19036 | Takeda 2016-01 | 27006499 | Loss | 1:160912872-160940932 | C | 8 |
| RCBTB1 | 55213 | 10061 | Takeda 2016-01 | 27006499 | Loss | 14:59203424-59217822 | D | 3 |
| RERE | 473 | 8101 | Takeda 2016-01 | 27006499 | Loss | 4:150540898-150579030 | A | 17 |
| REV1 | 51455 | 32309 | Takeda 2016-01 | 27006499 | Loss | 1:38006985-38125962 | A | 5 |
| REV3L | 5980 | 48147 | Takeda 2016-01 | 27006499 | Loss | 10:39780445-39795081 | B | 4 |
| RFWD2 | 64326 | 115565 | Takeda 2016-01 | 27006499 | Loss | 1:159264684-159306036 | B | 9 |
| RGMB | 285704 | 65355 | Takeda 2016-01 | 27006499 | Loss | 17:15805210-15821171 | D | 6 |
| RHEB | 6009 | 123916 | Takeda 2016-01 | 27006499 | Loss | 5:24817854-24829592 | D | 7 |
| RHOT1 | 55288 | 56803 | Takeda 2016-01 | 27006499 | Loss | 11:80252145-80255070 | D | 8 |
| RICTOR | 253260 | 34317 | Takeda 2016-01 | 27006499 | Loss | 15:6738000-6749651 | C | 5 |
| RLF | 6018 | 8243 | Takeda 2016-01 | 27006499 | Loss | 4:121141925-121160991 | D | 13 |
| RMDN1 | 51115 | 41093 | Takeda 2016-01 | 27006499 | Loss | 4:19578716-19617880 | C | 7 |
| RNF11 | 26994 | 32195 | Takeda 2016-01 | 27006499 | N/A | 4:109451098-109484728 | D | 6 |
| RNF130 | 55819 | 41267 | Takeda 2016-01 | 27006499 | Loss | 11:50024618-50048020 | C | 5 |
| RNF185 | 91445 | 34298 | Takeda 2016-01 | 27006499 | N/A | 11:3415982-3452363 | D | 4 |
| RNF38 | 152006 | 32550 | Takeda 2016-01 | 27006499 | Loss | 4:44122886-44150752 | D | 8 |
| RNF43 | 54894 | 37742 | Takeda 2016-01 | 27006499 | Loss | 11:87654357-87739187 | A | 10 |
| ROCK1 | 6093 | 55899 | Takeda 2016-01 | 27006499 | Loss | 18:10062337-10129181 | A | 14 |
| ROCK2 | 9475 | 21010 | Takeda 2016-01 | 27006499 | Loss | 12:16964099-16988922 | C | 15 |
| ROR2 | 4920 | 55831 | Takeda 2016-01 | 27006499 | Loss | 13:53167879-53187377 | B | 2 |
| RPL27 | 6155 | 105144 | Takeda 2016-01 | 27006499 | N/A | 11:101442298-101445529 | D | 1 |
| RPS16 | 6217 | 794 | Takeda 2016-01 | 27006499 | N/A | 7:28350652-28353155 | D | 2 |
| RRAGC | 64121 | 39141 | Takeda 2016-01 | 27006499 | N/A | 4:123917446-123936997 | D | 3 |
| RREB1 | 6239 | 2218 | Takeda 2016-01 | 27006499 | Loss | 13:37865372-37898945 | B | 18 |
| RSBN1 | 54665 | 10154 | Takeda 2016-01 | 27006499 | Loss | 3:103941104-103964636 | C | 5 |
| RSF1 | 51773 | 41142 | Takeda 2016-01 | 27006499 | Loss | 7:97593835-97670000 | A | 16 |
| RSPRY1 | 89970 | 12164 | Takeda 2016-01 | 27006499 | Loss | 8:94614658-94644038 | B | 3 |
| RTN3 | 10313 | 24934 | Takeda 2016-01 | 27006499 | Loss | 19:7429861-7492513 | B | 12 |
| RUNX1 | 861 | 1331 | Takeda 2016-01 | 27006499 | Loss | 16:92608530-92670569 | B | 9 |
| RXRA | 6256 | 2220 | Takeda 2016-01 | 27006499 | Loss | 2:27693634-27727568 | D | 5 |
| RYBP | 23429 | 8159 | Takeda 2016-01 | 27006499 | Loss | 6:100257346-100291074 | C | 12 |
| RYBP | 23429 | 8159 | Takeda 2016-01 | 27006499 | Loss | 6:100257346-100291074 | C | 12 |
| SAMD8 | 142891 | 41670 | Takeda 2016-01 | 27006499 | Loss | 14:21766267-21783557 | D | 5 |
| SARNP | 84324 | 41628 | Takeda 2016-01 | 27006499 | Loss | 10:128854480-128877898 | C | 12 |
| SASH1 | 23328 | 69182 | Takeda 2016-01 | 27006499 | Loss | 10:8841229-8874892 | C | 3 |
| SBNO1 | 55206 | 10055 | Takeda 2016-01 | 27006499 | Loss | 5:124374906-124386637 | D | 10 |
| SCAF4 | 57466 | 16227 | Takeda 2016-01 | 27006499 | Loss | 16:90226495-90264007 | C | 7 |
| SCAMP1 | 9522 | 37975 | Takeda 2016-01 | 27006499 | Loss | 13:94233442-94277312 | D | 6 |
| SCARB2 | 950 | 48353 | Takeda 2016-01 | 27006499 | Loss | 5:92471267-92484464 | D | 2 |
| SDC3 | 9672 | 7965 | Takeda 2016-01 | 27006499 | N/A | 4:130792537-130826319 | D | 3 |
| SDCBP | 6386 | 4110 | Takeda 2016-01 | 27006499 | Loss | 4:6364206-6390605 | C | 6 |
| SDHA | 6389 | 3073 | Takeda 2016-01 | 27006499 | Loss | 13:74329354-74343951 | D | 3 |
| SDHAF2 | 54949 | 32370 | Takeda 2016-01 | 27006499 | N/A | 19:10500513-10525209 | D | 4 |
| SEC16A | 9919 | 10533 | Takeda 2016-01 | 27006499 | N/A | 2:26409431-26445214 | D | 10 |
| SEC24D | 9871 | 40986 | Takeda 2016-01 | 27006499 | Loss | 3:123286452-123321770 | D | 5 |
| SEC31A | 22872 | 42056 | Takeda 2016-01 | 27006499 | Loss | 5:100377180-100416339 | C | 6 |
| SEC62 | 7095 | 2449 | Takeda 2016-01 | 27006499 | Loss | 3:30788306-30805955 | D | 5 |
| SEC63 | 11231 | 5220 | Takeda 2016-01 | 27006499 | Loss | 10:42781361-42839947 | B | 14 |
| SEL1L | 6400 | 31286 | Takeda 2016-01 | 27006499 | Loss | 12:91823859-91825319 | D | 4 |
| SETBP1 | 26040 | 9192 | Takeda 2016-01 | 27006499 | Loss | 18:78888219-78901284 | D | 3 |
| SETD3 | 84193 | 41748 | Takeda 2016-01 | 27006499 | Loss | 12:108141329-108179324 | C | 9 |
| SETD5 | 55209 | 12485 | Takeda 2016-01 | 27006499 | Loss | 6:113057800-113158984 | A | 25 |
| SF3B1 | 23451 | 6696 | Takeda 2016-01 | 27006499 | Loss | 1:54984920-55011504 | D | 3 |
| SFI1 | 9814 | 12707 | Takeda 2016-01 | 27006499 | Loss | 11:3127648-3216866 | A | 23 |
| SGPP2 | 130367 | 51848 | Takeda 2016-01 | 27006499 | Loss | 1:78395361-78430823 | C | 3 |
| SGSM3 | 27352 | 9249 | Takeda 2016-01 | 27006499 | Loss | 15:80985943-81028178 | D | 5 |
| SH2D4A | 63898 | 11117 | Takeda 2016-01 | 27006499 | Loss | 8:68303905-68334668 | C | 3 |
| SH3KBP1 | 30011 | 10971 | Takeda 2016-01 | 27006499 | Loss | X:159668268-159692461 | D | 3 |
| SHANK2 | 22941 | 105965 | Takeda 2016-01 | 27006499 | Loss | 7:144182484-144537213 | A | 4 |
| SHB | 6461 | 74458 | Takeda 2016-01 | 27006499 | Loss | 4:45458316-45481796 | C | 7 |
| SHOC2 | 8036 | 7219 | Takeda 2016-01 | 27006499 | Loss | 19:53912649-53961062 | B | 8 |
| SHROOM3 | 57619 | 9263 | Takeda 2016-01 | 27006499 | Loss | 5:92952240-92961038 | D | 8 |
| SIPA1L1 | 26037 | 9189 | Takeda 2016-01 | 27006499 | Loss | 12:82284519-82303502 | A | 13 |
| SIPA1L3 | 23094 | 77938 | Takeda 2016-01 | 27006499 | Loss | 7:29465410-29482998 | D | 4 |
| SIRT7 | 51547 | 56152 | Takeda 2016-01 | 27006499 | N/A | 11:120618372-120625240 | D | 1 |
| SLAIN2 | 57606 | 18952 | Takeda 2016-01 | 27006499 | Loss | 5:72912312-72944573 | C | 3 |
| SLC35A3 | 23443 | 40826 | Takeda 2016-01 | 27006499 | Loss | 3:116654966-116699088 | C | 7 |
| SLC35F5 | 80255 | 5745 | Takeda 2016-01 | 27006499 | Loss | 1:125588659-125646257 | A | 3 |
| SLC44A1 | 23446 | 11137 | Takeda 2016-01 | 27006499 | Loss | 4:53496656-53540715 | C | 4 |
| SLC4A7 | 9497 | 2680 | Takeda 2016-01 | 27006499 | Loss | 14:14732003-14756042 | C | 4 |
| SLC5A3 | 6526 | 31412 | Takeda 2016-01 | 27006499 | N/A | 16:92058322-92087473 | D | 1 |
| SLMAP | 7871 | 31428 | Takeda 2016-01 | 27006499 | Loss | 14:26459567-26525797 | A | 14 |
| SMAD2 | 4087 | 21197 | Takeda 2016-01 | 27006499 | Loss | 18:76245573-76305090 | C | 8 |
| SMAD4 | 4089 | 31310 | Takeda 2016-01 | 27006499 | Loss | 18:73610843-73715361 | A | 17 |
| SMAP1 | 60682 | 134676 | Takeda 2016-01 | 27006499 | Loss | 1:23851063-23892435 | D | 3 |
| SMARCA5 | 8467 | 55764 | Takeda 2016-01 | 27006499 | Loss | 8:80696961-80713075 | D | 8 |
| SMARCE1 | 6605 | 37727 | Takeda 2016-01 | 27006499 | N/A | 11:99209047-99231017 | D | 2 |
| SMCHD1 | 23347 | 23665 | Takeda 2016-01 | 27006499 | Loss | 17:71374182-71422662 | C | 1 |
| SMEK1 | 55671 | 69510 | Takeda 2016-01 | 27006499 | Loss | 12:101048217-101073041 | C | 16 |
| SMEK2 | 57223 | 68886 | Takeda 2016-01 | 27006499 | Loss | 11:29202859-29208710 | C | 9 |
| SMG1 | 23049 | 56697 | Takeda 2016-01 | 27006499 | Loss | 7:118183976-118220594 | D | 13 |
| SMG6 | 23293 | 23024 | Takeda 2016-01 | 27006499 | Loss | 11:75004387-75062936 | A | 10 |
| SMURF1 | 57154 | 10712 | Takeda 2016-01 | 27006499 | N/A | 5:144876495-144965847 | D | 4 |
| SMURF2 | 64750 | 41490 | Takeda 2016-01 | 27006499 | Loss | 11:106873357-106898221 | C | 8 |
| SNAPC2 | 6618 | 2318 | Takeda 2016-01 | 27006499 | N/A | 8:4253080-4256220 | D | 2 |
| SND1 | 27044 | 8665 | Takeda 2016-01 | 27006499 | Loss | 6:28788018-28832010 | A | 17 |
| SNTB2 | 6645 | 4911 | Takeda 2016-01 | 27006499 | Loss | 8:106939183-106964087 | D | 6 |
| SNX13 | 23161 | 41011 | Takeda 2016-01 | 27006499 | Loss | 12:35045514-35083479 | C | 6 |
| SOCS5 | 9655 | 10495 | Takeda 2016-01 | 27006499 | N/A | 17:87107679-87137583 | D | 4 |
| SON | 6651 | 10551 | Takeda 2016-01 | 27006499 | N/A | 16:91647506-91679221 | D | 13 |
| SORBS2 | 8470 | 83295 | Takeda 2016-01 | 27006499 | Loss | 8:45655096-45688788 | C | 8 |
| SOX13 | 9580 | 4159 | Takeda 2016-01 | 27006499 | Loss | 1:133276715-133514670 | A | 1 |
| SPATA1 | 100505741 | 23356 | Takeda 2016-01 | 27006499 | Loss | 3:146476604-146486422 | C | 2 |
| SPATA5 | 166378 | 56920 | Takeda 2016-01 | 27006499 | Loss | 3:37494770-37503594 | D | 7 |
| SPEN | 23013 | 124461 | Takeda 2016-01 | 27006499 | N/A | 4:141467890-141538597 | D | 8 |
| SPOP | 8405 | 68354 | Takeda 2016-01 | 27006499 | Loss | 11:95424134-95468012 | C | 6 |
| SPPL3 | 121665 | 15563 | Takeda 2016-01 | 27006499 | Loss | 5:115012051-115061908 | C | 15 |
| SPRED1 | 161742 | 24919 | Takeda 2016-01 | 27006499 | Loss | 2:117116895-117171483 | B | 16 |
| SPRED2 | 200734 | 24918 | Takeda 2016-01 | 27006499 | Loss | 11:19978247-20014812 | C | 9 |
| SPTBN1 | 6711 | 2354 | Takeda 2016-01 | 27006499 | Loss | 11:30171100-30184263 | C | 17 |
| SPTLC2 | 9517 | 21610 | Takeda 2016-01 | 27006499 | Loss | 12:87353869-87378693 | D | 6 |
| SRCAP | 10847 | 38213 | Takeda 2016-01 | 27006499 | Loss | 7:127524482-127564029 | D | 4 |
| SRR | 63826 | 22775 | Takeda 2016-01 | 27006499 | N/A | 11:74906509-74925948 | D | 6 |
| ST13 | 6767 | 2921 | Takeda 2016-01 | 27006499 | Loss | 15:81368971-81374797 | D | 10 |
| ST3GAL2 | 6483 | 5047 | Takeda 2016-01 | 27006499 | Gain | 8:110886150-110949734 | B | 3 |
| ST5 | 6764 | 3951 | Takeda 2016-01 | 27006499 | Loss | 7:109594870-109619770 | C | 8 |
| ST7L | 54879 | 14910 | Takeda 2016-01 | 27006499 | Loss | 3:104845173-104928627 | C | 7 |
| STARD13 | 90627 | 64844 | Takeda 2016-01 | 27006499 | Loss | 5:151074890-151092497 | D | 8 |
| STARD7 | 56910 | 32463 | Takeda 2016-01 | 27006499 | N/A | 2:127270218-127298932 | D | 4 |
| STIM1 | 6786 | 20681 | Takeda 2016-01 | 27006499 | Loss | 7:102304363-102319010 | C | 7 |
| STK24 | 8428 | 20793 | Takeda 2016-01 | 27006499 | Loss | 14:121311716-121341952 | C | 15 |
| STK3 | 6788 | 48420 | Takeda 2016-01 | 27006499 | Loss | 15:34879835-35079157 | A | 2 |
| STK38L | 23012 | 56299 | Takeda 2016-01 | 27006499 | Loss | 6:146734335-146751932 | D | 4 |
| STRADA | 92335 | 12448 | Takeda 2016-01 | 27006499 | Loss | 11:106129627-106217654 | B | 3 |
| STXBP5 | 134957 | 16402 | Takeda 2016-01 | 27006499 | Loss | 10:9864291-9876000 | D | 5 |
| SUV420H1 | 51111 | 32351 | Takeda 2016-01 | 27006499 | Loss | 19:3777753-3817561 | C | 7 |
| SVIL | 6840 | 25090 | Takeda 2016-01 | 27006499 | Loss | 18:4924919-4949597 | D | 4 |
| SYMPK | 8189 | 37969 | Takeda 2016-01 | 27006499 | N/A | 7:19024377-19054618 | D | 4 |
| SYNJ2BP | 55333 | 10161 | Takeda 2016-01 | 27006499 | Loss | 12:81475564-81500388 | D | 4 |
| TAB2 | 23118 | 9019 | Takeda 2016-01 | 27006499 | Loss | 10:7933791-7952818 | D | 14 |
| TAF5L | 27097 | 8676 | Takeda 2016-01 | 27006499 | N/A | 8:123996318-124021309 | D | 2 |
| TANC1 | 85461 | 18946 | Takeda 2016-01 | 27006499 | Loss | 2:59777243-59816633 | B | 5 |
| TAOK1 | 57551 | 27041 | Takeda 2016-01 | 27006499 | Loss | 11:77563889-77603379 | B | 21 |
| TAOK3 | 51347 | 83279 | Takeda 2016-01 | 27006499 | Loss | 5:117154437-117201361 | C | 9 |
| TATDN1 | 83940 | 57158 | Takeda 2016-01 | 27006499 | Loss | 15:58891507-58917722 | D | 4 |
| TBC1D15 | 64786 | 11249 | Takeda 2016-01 | 27006499 | Loss | 10:115219501-115251701 | C | 5 |
| TBC1D20 | 128637 | 32574 | Takeda 2016-01 | 27006499 | Loss | 2:152293879-152296829 | D | 5 |
| TBC1D5 | 9779 | 8834 | Takeda 2016-01 | 27006499 | Loss | 17:51018475-51088605 | A | 3 |
| TBL1X | 6907 | 4128 | Takeda 2016-01 | 27006499 | Loss | X:77609651-77654592 | C | 12 |
| TBL1XR1 | 79718 | 69382 | Takeda 2016-01 | 27006499 | Loss | 3:22114026-22121379 | C | 14 |
| TCF4 | 6925 | 2407 | Takeda 2016-01 | 27006499 | Loss | 18:69652214-69673989 | A | 7 |
| TCF7L2 | 6934 | 7564 | Takeda 2016-01 | 27006499 | Loss | 19:55749944-55778379 | A | 13 |
| TENM3 | 55714 | 22673 | Takeda 2016-01 | 27006499 | Loss | 8:48590228-48660889 | A | 6 |
| TET2 | 54790 | 49498 | Takeda 2016-01 | 27006499 | Loss | 3:133471447-133494992 | D | 4 |
| TET3 | 200424 | 35360 | Takeda 2016-01 | 27006499 | Loss | 6:83421282-83450610 | D | 7 |
| TFB2M | 64216 | 7362 | Takeda 2016-01 | 27006499 | N/A | 1:179528055-179546267 | D | 2 |
| THADA | 63892 | 75175 | Takeda 2016-01 | 27006499 | Loss | 17:84292293-84397488 | A | 5 |
| TICAM1 | 148022 | 8605 | Takeda 2016-01 | 27006499 | N/A | 17:56269319-56276786 | D | 1 |
| TIMM23 | 100287932 | 9651 | Takeda 2016-01 | 27006499 | Loss | 14:32178262-32204197 | B | 8 |
| TIPRL | 261726 | 7140 | Takeda 2016-01 | 27006499 | N/A | 1:165212286-165236996 | D | 2 |
| TJP2 | 9414 | 3541 | Takeda 2016-01 | 27006499 | N/A | 19:24094523-24225026 | C | 10 |
| TLE1 | 7088 | 21058 | Takeda 2016-01 | 27006499 | Loss | 4:72114457-72168721 | B | 8 |
| TLK1 | 9874 | 130657 | Takeda 2016-01 | 27006499 | Loss | 2:70757112-70776807 | C | 14 |
| TLK2 | 11011 | 4993 | Takeda 2016-01 | 27006499 | Loss | 11:105219158-105239634 | C | 9 |
| TM2D2 | 83877 | 12328 | Takeda 2016-01 | 27006499 | N/A | 8:25017211-25023259 | D | 1 |
| TM9SF2 | 9375 | 21004 | Takeda 2016-01 | 27006499 | N/A | 14:122107038-122159604 | D | 8 |
| TM9SF3 | 56889 | 10588 | Takeda 2016-01 | 27006499 | Loss | 19:41225553-41248301 | C | 10 |
| TMC5 | 79838 | 11713 | Takeda 2016-01 | 27006499 | Loss | 7:118659308-118697414 | D | 1 |
| TMED10 | 10972 | 4972 | Takeda 2016-01 | 27006499 | N/A | 12:85340614-85374717 | D | 6 |
| TMED10 | 10972 | 4972 | Takeda 2016-01 | 27006499 | N/A | 12:85340614-85374717 | D | 6 |
| TMEM126A | 84233 | 11939 | Takeda 2016-01 | 27006499 | Loss | 7:90448941-90451870 | D | 5 |
| TMEM131 | 23505 | 32428 | Takeda 2016-01 | 27006499 | Loss | 1:36884389-36902112 | C | 6 |
| TMEM135 | 65084 | 11295 | Takeda 2016-01 | 27006499 | Loss | 7:89204159-89282390 | C | 5 |
| TMEM164 | 84187 | 12985 | Takeda 2016-01 | 27006499 | Loss | X:142729007-142762350 | D | 4 |
| TMEM184B | 25829 | 6396 | Takeda 2016-01 | 27006499 | Loss | 15:79389652-79401313 | D | 3 |
| TMEM230 | 29058 | 8561 | Takeda 2016-01 | 27006499 | N/A | 2:132239492-132247807 | D | 3 |
| TMEM245 | 23731 | 41841 | Takeda 2016-01 | 27006499 | Loss | 4:56877993-56894126 | D | 4 |
| TMEM248 | 55069 | 9951 | Takeda 2016-01 | 27006499 | N/A | 5:130217081-130243666 | D | 5 |
| TMEM39A | 55254 | 9985 | Takeda 2016-01 | 27006499 | Loss | 16:38576437-38592308 | D | 3 |
| TMEM57 | 55219 | 14449 | Takeda 2016-01 | 27006499 | Loss | 4:134814492-134827691 | D | 3 |
| TMEM63A | 9725 | 101673 | Takeda 2016-01 | 27006499 | Loss | 1:180943672-180956964 | D | 2 |
| TNKS | 8658 | 18405 | Takeda 2016-01 | 27006499 | Loss | 8:34882546-34904519 | B | 15 |
| TNKS2 | 80351 | 11890 | Takeda 2016-01 | 27006499 | Loss | 19:36839517-36877904 | C | 14 |
| TNPO1 | 3842 | 5358 | Takeda 2016-01 | 27006499 | Loss | 13:98848624-98869059 | C | 19 |
| TNPO3 | 23534 | 40848 | Takeda 2016-01 | 27006499 | Loss | 6:29591620-29601885 | D | 8 |
| TNRC6B | 23112 | 66194 | Takeda 2016-01 | 27006499 | Loss | 15:80799526-80867976 | A | 17 |
| TNS3 | 64759 | 49713 | Takeda 2016-01 | 27006499 | Loss | 11:8599449-8614087 | B | 8 |
| TOP1 | 7150 | 2467 | Takeda 2016-01 | 27006499 | Loss | 2:160644411-160710802 | A | 15 |
| TOP2B | 7155 | 134711 | Takeda 2016-01 | 27006499 | Loss | 14:16384296-16404468 | D | 7 |
| TOX | 9760 | 8822 | Takeda 2016-01 | 27006499 | Loss | 4:6773390-6790989 | A | 5 |
| TPP2 | 7174 | 2471 | Takeda 2016-01 | 27006499 | Loss | 1:43976323-44002907 | C | 4 |
| TRA2B | 6434 | 20965 | Takeda 2016-01 | 27006499 | N/A | 16:22244549-22266005 | D | 11 |
| TRAF3 | 7187 | 7981 | Takeda 2016-01 | 27006499 | Loss | 12:111215639-111243383 | C | 6 |
| TRAPPC8 | 22878 | 40993 | Takeda 2016-01 | 27006499 | Loss | 18:20842992-20860430 | C | 7 |
| TRIAP1 | 51499 | 41131 | Takeda 2016-01 | 27006499 | N/A | 5:115341225-115343569 | D | 1 |
| TRIM10 | 10107 | 4932 | Takeda 2016-01 | 27006499 | N/A | 17:36869574-36877833 | D | 2 |
| TRIM44 | 54765 | 9731 | Takeda 2016-01 | 27006499 | Loss | 2:102323643-102338404 | C | 9 |
| TRIM72 | 493829 | 66924 | Takeda 2016-01 | 27006499 | N/A | 7:128003949-128011033 | D | 2 |
| TRIO | 7204 | 20847 | Takeda 2016-01 | 27006499 | Loss | 15:27878389-27936854 | B | 9 |
| TRIP12 | 9320 | 44226 | Takeda 2016-01 | 27006499 | Loss | 1:84767216-84829245 | B | 9 |
| TRPC4AP | 26133 | 9224 | Takeda 2016-01 | 27006499 | Loss | 2:155664011-155673858 | C | 9 |
| TRPM7 | 54822 | 9774 | Takeda 2016-01 | 27006499 | Loss | 2:126811081-126889860 | A | 6 |
| TSPAN14 | 81619 | 23717 | Takeda 2016-01 | 27006499 | N/A | 14:40906445-40966807 | D | 7 |
| TTC17 | 55761 | 10100 | Takeda 2016-01 | 27006499 | Loss | 2:94339575-94398659 | C | 3 |
| TTC3 | 7267 | 2487 | Takeda 2016-01 | 27006499 | Loss | 16:94398051-94412493 | D | 6 |
| TULP4 | 56995 | 32467 | Takeda 2016-01 | 27006499 | Loss | 17:6101126-6131598 | C | 4 |
| TXLNG | 55787 | 23097 | Takeda 2016-01 | 27006499 | Loss | X:162772029-162802472 | D | 6 |
| TXNDC11 | 51061 | 9301 | Takeda 2016-01 | 27006499 | N/A | 16:11074911-11134650 | D | 9 |
| TXNRD2 | 10587 | 4701 | Takeda 2016-01 | 27006499 | N/A | 16:18426384-18479073 | D | 4 |
| UBAC2 | 337867 | 18642 | Takeda 2016-01 | 27006499 | Loss | 14:121975452-122002808 | C | 7 |
| UBALD1 | 124402 | 17055 | Takeda 2016-01 | 27006499 | N/A | 16:4874778-4880315 | D | 4 |
| UBAP2 | 55833 | 73649 | Takeda 2016-01 | 27006499 | Loss | 4:41186845-41235799 | B | 12 |
| UBE2D3 | 7323 | 123914 | Takeda 2016-01 | 27006499 | Loss | 3:135437720-135471547 | C | 15 |
| UBE2E3 | 10477 | 4636 | Takeda 2016-01 | 27006499 | N/A | 2:78868124-78921293 | D | 7 |
| UBE2F | 140739 | 12212 | Takeda 2016-01 | 27006499 | Loss | 1:91233688-91269133 | C | 3 |
| UBE2H | 7328 | 103894 | Takeda 2016-01 | 27006499 | Loss | 6:30273510-30283775 | D | 11 |
| UBE2K | 3093 | 3903 | Takeda 2016-01 | 27006499 | N/A | 5:65537233-65598988 | D | 9 |
| UBE2L3 | 7332 | 43226 | Takeda 2016-01 | 27006499 | Loss | 16:17149687-17197299 | C | 12 |
| UBE2N | 7334 | 128406 | Takeda 2016-01 | 27006499 | N/A | 10:95515162-95545658 | D | 11 |
| UBE3A | 7337 | 7988 | Takeda 2016-01 | 27006499 | Loss | 7:59240261-59262232 | C | 13 |
| UBL3 | 5412 | 5153 | Takeda 2016-01 | 27006499 | Loss | 5:148522463-148547392 | C | 6 |
| UBN1 | 29855 | 9656 | Takeda 2016-01 | 27006499 | Loss | 16:5027009-5076110 | C | 6 |
| UBQLN1 | 29979 | 137258 | Takeda 2016-01 | 27006499 | Loss | 13:58174650-58214062 | D | 12 |
| UBR3 | 130507 | 52092 | Takeda 2016-01 | 27006499 | Loss | 2:69919913-69964262 | B | 6 |
| UBR4 | 23352 | 10804 | Takeda 2016-01 | 27006499 | N/A | 4:139352609-139489588 | D | 8 |
| UBR5 | 51366 | 9295 | Takeda 2016-01 | 27006499 | Loss | 15:38035498-38070452 | C | 16 |
| UBR7 | 55148 | 11998 | Takeda 2016-01 | 27006499 | N/A | 12:102757975-102777701 | D | 3 |
| UBXN7 | 26043 | 45585 | Takeda 2016-01 | 27006499 | Loss | 16:32347970-32358070 | D | 6 |
| UGGT1 | 56886 | 10586 | Takeda 2016-01 | 27006499 | Loss | 1:36188977-36206708 | C | 3 |
| UGP2 | 7360 | 121676 | Takeda 2016-01 | 27006499 | N/A | 11:21321138-21371201 | D | 5 |
| UPF2 | 26019 | 6101 | Takeda 2016-01 | 27006499 | Loss | 2:6025834-6060300 | B | 1 |
| UQCR11 | 10975 | 4974 | Takeda 2016-01 | 27006499 | N/A | 10:80402997-80406830 | D | 3 |
| URB2 | 9816 | 8859 | Takeda 2016-01 | 27006499 | N/A | 8:124021508-124048505 | D | 1 |
| USO1 | 8615 | 2754 | Takeda 2016-01 | 27006499 | Loss | 5:92144263-92172124 | C | 6 |
| USP10 | 9100 | 31294 | Takeda 2016-01 | 27006499 | Loss | 8:119921129-119946033 | D | 8 |
| USP31 | 57478 | 44504 | Takeda 2016-01 | 27006499 | Loss | 7:121689046-121703693 | D | 5 |
| USP34 | 9736 | 40978 | Takeda 2016-01 | 27006499 | Loss | 11:23309737-23363462 | A | 14 |
| USP47 | 55031 | 9929 | Takeda 2016-01 | 27006499 | Loss | 7:112036555-112067314 | C | 13 |
| USP49 | 25862 | 10235 | Takeda 2016-01 | 27006499 | Loss | 17:47626838-47658761 | C | 3 |
| USP6NL | 9712 | 6879 | Takeda 2016-01 | 27006499 | Loss | 2:6348487-6386866 | C | 11 |
| USP7 | 7874 | 2592 | Takeda 2016-01 | 27006499 | Loss | 16:8689389-8703830 | D | 12 |
| USP9X | 8239 | 3418 | Takeda 2016-01 | 27006499 | Loss | X:13078613-13153997 | B | 11 |
| UTP18 | 51096 | 41087 | Takeda 2016-01 | 27006499 | N/A | 11:93859243-93885766 | D | 1 |
| UTRN | 7402 | 21398 | Takeda 2016-01 | 27006499 | Loss | 10:12468051-12478296 | A | 11 |
| UVRAG | 7405 | 31150 | Takeda 2016-01 | 27006499 | Loss | 7:99017540-99035117 | B | 9 |
| VCL | 7414 | 7594 | Takeda 2016-01 | 27006499 | Loss | 14:20934147-21049943 | B | 8 |
| VCP | 7415 | 5168 | Takeda 2016-01 | 27006499 | N/A | 4:42979963-43000507 | D | 5 |
| VGLL4 | 9686 | 18603 | Takeda 2016-01 | 27006499 | Loss | 6:114851218-114896678 | B | 13 |
| VPS13B | 157680 | 49516 | Takeda 2016-01 | 27006499 | Loss | 15:35760630-35792671 | A | 3 |
| VPS13D | 55187 | 15583 | Takeda 2016-01 | 27006499 | Loss | 4:145124505-145143570 | B | 11 |
| VPS16 | 64601 | 7116 | Takeda 2016-01 | 27006499 | N/A | 2:130424339-130444269 | D | 2 |
| VPS53 | 55275 | 6264 | Takeda 2016-01 | 27006499 | Loss | 11:76105678-76124691 | D | 3 |
| VPS54 | 51542 | 5605 | Takeda 2016-01 | 27006499 | Loss | 11:21236081-21322375 | A | 7 |
| VTA1 | 51534 | 6473 | Takeda 2016-01 | 27006499 | Loss | 10:14660134-14686498 | C | 1 |
| WAC | 51322 | 41148 | Takeda 2016-01 | 27006499 | Loss | 18:7884817-7912398 | C | 26 |
| WAPAL | 23063 | 41002 | Takeda 2016-01 | 27006499 | Loss | 14:34672578-34754645 | A | 15 |
| WASF2 | 10163 | 86743 | Takeda 2016-01 | 27006499 | Loss | 4:133098918-133157826 | A | 11 |
| WBP1L | 54838 | 9839 | Takeda 2016-01 | 27006499 | N/A | 19:46599084-46657389 | D | 8 |
| WDR20 | 91833 | 32684 | Takeda 2016-01 | 27006499 | Loss | 12:110762644-110806529 | D | 9 |
| WDR33 | 55339 | 56807 | Takeda 2016-01 | 27006499 | Loss | 18:31789033-31842744 | B | 17 |
| WDR37 | 22884 | 40914 | Takeda 2016-01 | 27006499 | N/A | 13:8802995-8871909 | D | 3 |
| WHSC1L1 | 54904 | 56960 | Takeda 2016-01 | 27006499 | Loss | 8:25629424-25693926 | B | 12 |
| WLS | 79971 | 11779 | Takeda 2016-01 | 27006499 | Loss | 3:159839695-159935175 | C | 4 |
| WNK1 | 65125 | 14253 | Takeda 2016-01 | 27006499 | Loss | 6:119993983-120021845 | B | 18 |
| XPO4 | 64328 | 10733 | Takeda 2016-01 | 27006499 | Loss | 14:57595726-57653419 | C | 5 |
| XPO6 | 23214 | 12544 | Takeda 2016-01 | 27006499 | Loss | 7:126097847-126163759 | B | 8 |
| XPO7 | 23039 | 22857 | Takeda 2016-01 | 27006499 | Loss | 14:70733317-70766432 | B | 13 |
| XRCC3 | 7517 | 36178 | Takeda 2016-01 | 27006499 | N/A | 12:111803192-111813873 | D | 2 |
| XRN2 | 22803 | 6927 | Takeda 2016-01 | 27006499 | Loss | 2:147034224-147059305 | D | 9 |
| YLPM1 | 56252 | 87707 | Takeda 2016-01 | 27006499 | Loss | 12:85011697-85021919 | D | 6 |
| YTHDF3 | 253943 | 34991 | Takeda 2016-01 | 27006499 | Loss | 3:16193317-16218319 | D | 14 |
| YY1 | 7528 | 2556 | Takeda 2016-01 | 27006499 | Loss | 12:108803378-108816519 | D | 13 |
| ZBTB2 | 57621 | 10837 | Takeda 2016-01 | 27006499 | Loss | 10:4382464-4394181 | D | 4 |
| ZBTB8OS | 339487 | 12083 | Takeda 2016-01 | 27006499 | Loss | 4:129332372-129349982 | D | 5 |
| ZC3H14 | 79882 | 32605 | Takeda 2016-01 | 27006499 | Loss | 12:98745039-98756729 | D | 4 |
| ZC3H3 | 23144 | 9029 | Takeda 2016-01 | 27006499 | N/A | 15:75754432-75841915 | D | 4 |
| ZC3H7A | 29066 | 8563 | Takeda 2016-01 | 27006499 | Loss | 16:11148235-11191684 | B | 7 |
| ZCCHC7 | 84186 | 22217 | Takeda 2016-01 | 27006499 | Loss | 4:44780335-44850774 | A | 13 |
| ZEB2 | 9839 | 8868 | Takeda 2016-01 | 27006499 | Loss | 2:45008488-45058676 | B | 10 |
| ZFAND3 | 60685 | 11077 | Takeda 2016-01 | 27006499 | Loss | 17:30045872-30083600 | B | 16 |
| ZFAND6 | 54469 | 10372 | Takeda 2016-01 | 27006499 | Loss | 7:84647193-84663305 | D | 9 |
| ZFP91 | 80829 | 43144 | Takeda 2016-01 | 27006499 | Loss | 19:12775320-12788115 | D | 10 |
| ZFR | 51663 | 8009 | Takeda 2016-01 | 27006499 | Loss | 15:12165790-12196374 | C | 14 |
| ZFYVE20 | 64145 | 41477 | Takeda 2016-01 | 27006499 | Loss | 6:92121601-92209587 | B | 2 |
| ZFYVE21 | 79038 | 11448 | Takeda 2016-01 | 27006499 | N/A | 12:111814170-111828388 | D | 3 |
| ZMIZ1 | 57178 | 10667 | Takeda 2016-01 | 27006499 | Loss | 14:25460364-25484841 | B | 26 |
| ZMYM2 | 7750 | 12631 | Takeda 2016-01 | 27006499 | Loss | 14:56941539-56958817 | C | 16 |
| ZMYND8 | 23613 | 32679 | Takeda 2016-01 | 27006499 | Loss | 2:165799391-165825961 | D | 5 |
| ZNF512B | 57473 | 69314 | Takeda 2016-01 | 27006499 | Loss | 2:181570044-181609434 | C | 2 |
| ZNRF1 | 84937 | 41858 | Takeda 2016-01 | 27006499 | Loss | 8:111546803-111617464 | A | 7 |
| ZNRF3 | 84133 | 46592 | Takeda 2016-01 | 27006499 | Loss | 11:5325933-5342022 | B | 11 |
| ZRANB1 | 54764 | 9728 | Takeda 2016-01 | 27006499 | Loss | 7:132973399-132976328 | D | 8 |

Table S3: Functional enrichment analysis results from ‘G:profiler’

| **Source** | **term_name** | **term_id** | **Adjusted P value** |
| --- | --- | --- | --- |
| GO:MF | enzyme binding | GO:0019899 | 0.028 |
| GO:BP | protein folding | GO:0006457 | 0.044 |
| GO:CC | cytoplasm | GO:0005737 | 0.009 |
| GO:CC | intracellular membrane-bounded organelle | GO:0043231 | 0.029 |
| GO:CC | membrane-bounded organelle | GO:0043227 | 0.029 |
| TF | Factor: ER71:SREBP-2; motif: NTSACGTGACGGAARY; match class: 1 | TF:M08457_1 | 0.002 |
| TF | Factor: Sp1; motif: NGGGGCGGGGN | TF:M07395 | 0.040 |
| MIRNA | hsa-miR-580-3p | MIRNA:hsa-miR-580-3p | 0.005 |
| MIRNA | hsa-miR-155-5p | MIRNA:hsa-miR-155-5p | 0.039 |
| HPA | urinary bladder; urothelial cells[High] | HPA:0610833 | 0.003 |
| HPA | heart muscle; cardiomyocytes[â‰¥Low] | HPA:0241101 | 0.012 |
| HPA | heart muscle | HPA:0240000 | 0.012 |
| HPA | cervix; squamous epithelial cells[â‰¥Medium] | HPA:0630222 | 0.020 |
| HPA | prostate; glandular cells[High] | HPA:0390053 | 0.028 |
| HPA | esophagus; squamous epithelial cells[â‰¥Medium] | HPA:0190222 | 0.029 |
| HPA | bone marrow; hematopoietic cells[â‰¥Medium] | HPA:0040082 | 0.034 |
| HPA | tonsil; non-germinal center cells[â‰¥Low] | HPA:0600441 | 0.038 |
| HPA | vagina; squamous epithelial cells[High] | HPA:0620223 | 0.038 |
| HPA | gallbladder; glandular cells[High] | HPA:0220053 | 0.047 |
